# Characterization and Relative Quantitation of Wheat, Rye, and Barley Gluten Protein Types by Liquid Chromatography–Tandem Mass Spectrometry

**DOI:** 10.3389/fpls.2019.01530

**Published:** 2019-12-13

**Authors:** Barbara Lexhaller, Michelle L. Colgrave, Katharina A. Scherf

**Affiliations:** ^1^Leibniz-Institute for Food Systems Biology at the Technical University of Munich, Freising, Germany; ^2^CSIRO Agriculture and Food, St Lucia, QLD, Australia; ^3^School of Science, Edith Cowan University, Joondalup, WA, Australia; ^4^Department of Bioactive and Functional Food Chemistry, Institute of Applied Biosciences, Karlsruhe Institute of Technology (KIT), Karlsruhe, Germany

**Keywords:** allergy, amylase/trypsin-inhibitor, celiac disease, gliadin, gluten, mass spectrometry, non-celiac gluten sensitivity, proteomics

## Abstract

The consumption of wheat, rye, and barley may cause adverse reactions to wheat such as celiac disease, non-celiac gluten/wheat sensitivity, or wheat allergy. The storage proteins (gluten) are known as major triggers, but also other functional protein groups such as α-amylase/trypsin-inhibitors or enzymes are possibly harmful for people suffering of adverse reactions to wheat. Gluten is widely used as a collective term for the complex protein mixture of wheat, rye or barley and can be subdivided into the following gluten protein types (GPTs): α-gliadins, γ-gliadins, ω5-gliadins, ω1,2-gliadins, high- and low-molecular-weight glutenin subunits of wheat, ω-secalins, high-molecular-weight secalins, γ-75k-secalins and γ-40k-secalins of rye, and C-hordeins, γ-hordeins, B-hordeins, and D-hordeins of barley. GPTs isolated from the flours are useful as reference materials for clinical studies, diagnostics or in food analyses and to elucidate disease mechanisms. A combined strategy of protein separation according to solubility followed by preparative reversed-phase high-performance liquid chromatography was employed to purify the GPTs according to hydrophobicity. Due to the heterogeneity of gluten proteins and their partly polymeric nature, it is a challenge to obtain highly purified GPTs with only one protein group. Therefore, it is essential to characterize and identify the proteins and their proportions in each GPT. In this study, the complexity of gluten from wheat, rye, and barley was demonstrated by identification of the individual proteins employing an undirected proteomics strategy involving liquid chromatography–tandem mass spectrometry of tryptic and chymotryptic hydrolysates of the GPTs. Different protein groups were obtained and the relative composition of the GPTs was revealed. Multiple reaction monitoring liquid chromatography–tandem mass spectrometry was used for the relative quantitation of the most abundant gluten proteins. These analyses also allowed the identification of known wheat allergens and celiac disease-active peptides. Combined with functional assays, these findings may shed light on the mechanisms of gluten/wheat-related disorders and may be useful to characterize reference materials for analytical or diagnostic assays more precisely.

## Introduction

Cereals including wheat, rice, and maize are the most important staple foods for mankind worldwide. However, the consumption of wheat and the closely related cereals rye and barley may cause adverse reactions to wheat such as celiac disease (CD), non-celiac gluten sensitivity (NCGS), or wheat allergy (Sapone et al., 2012; Ludvigsson et al., 2013; Catassi et al., 2017, for review). The triggers are mainly the storage proteins (gluten), but non-gluten proteins like α-amylase/trypsin-inhibitors (ATIs), lipid transfer proteins, puroindolines, or β-amylases are also immunoreactive (Tatham and Shewry, 2008; Scherf, 2019, for review). Gluten is widely used as a collective term for the complex protein mixture of wheat, rye, or barley, which is not soluble in water or salt solution (Codex Alimentarius Commission, 2015). Traditionally, cereal proteins are classified into the so-called Osborne fractions that can be obtained with salt solution (albumins/globulins), 60% aqueous ethanol (prolamins), and a reducing solution of 50% propanol and Tris-hydrochloride buffer (Tris-HCl) (glutelins).

Albumins/globulins are mainly protective or metabolic proteins whereas prolamins and glutelins constitute the storage proteins called gluten. Gluten is composed of gliadins (prolamins) and glutenins (glutelins) in wheat, secalins in rye and hordeins in barley (Scherf et al., 2016). Each gluten fraction can be further subdivided into the respective gluten protein types (GPTs) by preparative reversed-phase high-performance liquid chromatography (RP-HPLC) according to their characteristic retention times. The GPTs of wheat prolamins are α-gliadins, γ-gliadins, ω1,2-gliadins, and ω5-gliadins, and wheat glutelins are divided into high- (HMW-GS) and low-molecular-weight glutenin subunits (LMW-GS). The GPTs of rye are called ω-secalins, HMW-secalins, γ-75k-secalins, and γ-40k-secalins and the barley GPTs are B-hordeins, C-hordeins, D-hordeins, and γ-hordeins (Scherf et al., 2016). These GPTs can be classified into three different groups according to their homologous amino acid sequences and similar molecular weights: LMW group, medium-molecular-weight group and HMW group (**Table 1**). Each GPT contains numerous different proteins, which differ partly only by exchange, deletion or insertion of single amino acids in their sequences. Proteins of the HMW group occur in the glutelin fraction as polymers linked by interchain disulfide bonds. Previous studies revealed similar molecular weights (70–90 kDa) and homologous amino acid sequences of D-hordeins, HMW-secalins and HMW-GS (Field et al., 1982; Shewry et al., 1988; Gellrich et al., 2003). The amino acid sequences contain repetitive units such as QQPGQG, YYPTSP, or QQP and QPG. Differences between the proteins result from modifications of single amino acids or the arrangement and number of the repetitive units. The medium-molecular-weight group proteins mainly occur as monomers in the prolamin fraction and have molecular weights around 40–50 kDa, with the exception of ω5-gliadins (60–68 kDa) that are unique for wheat. The typical repetitive unit for ω5-gliadins is QQQPF, and QPQQPFP is characteristic for ω1,2-gliadins, ω-secalins and C-hordeins. The LMW group consists of monomeric (α-gliadins, γ-gliadins, γ-40k-secalins, and γ-hordeins) and polymeric proteins (LMW-GS, γ-75k-secalins, and B-hordeins). Their molecular weights range from 28 to 35 kDa, except for γ-75k-secalins with a molecular weight around 50 kDa. The proteins of the LMW group comprise unique repetitive units such as QPQPFPPQQPY (α-gliadins), QQPQQPFP (γ-gliadins, γ-75k-secalins, and B-hordeins), and QQPPFS (LMW-GS).

**Table 1:** Gluten protein types and their classification according to molecular weight (Scherf et al., 2016).

Group	Wheat	Rye	Barley
HMW	HMW-GS	HMW-secalins	D-hordeins
MMW	ω1,2-gliadins	ω-secalins	C-hordeins
	ω5-gliadins	-	-
LMW	LMW-GS	γ-75k-secalins	B-hordeins
	γ-gliadins	γ-40k-secalins	γ-hordeins
	α-gliadins	-	-

GS, glutenin subunits; HMW, high-molecular-weight; MMW, medium-molecular-weight; LMW, low-molecular-weight.

These characteristic features of the GPTs are known to contribute to the CD-immunoreactivity of wheat, rye, and barley, because most CD-active peptides are derived from these repetitive units. For example, the T-cell epitopes QG**YYPTSP**Q (DQ8.5-glut-H1), **QQPQQPFP**Q (DQ2.5-glia-γ4c), or **QQPQQPFP**Q (DQ8-glia-γ1a) contain typical repetitive units highlighted in bold (Sollid et al., 2012). Beside CD, a wide range of wheat, rye, and barley proteins are potential allergens or triggers of innate immunity in NCGS. The recently published reference sequence RefSeq v1.0 of the hexaploid common wheat genome (International Wheat Genome Sequencing Consortium (IWGSC), 2018) provides further insights as the first reference to which known immunoreactive gluten and non-gluten proteins can be annotated (Juhasz et al., 2018).

Numerous studies have demonstrated the complexity of gluten as a mixture of closely related, but distinct proteins (Arentz-Hansen et al., 2000; Dupont et al., 2011; Colgrave et al., 2013; Schalk et al., 2017). Their similarity poses major difficulties in clearly separating gluten into well-defined gluten protein fractions, GPTs and especially individual gluten proteins (Mamone et al., 2009; Ellis et al., 2011; Lagrain et al., 2013). One strategy is to combine separation according to solubility (Osborne fractionation) with subsequent fractionation according to polarity by preparative RP-HPLC. However, the ultraviolet signal at a specific retention time during preparative RP-HPLC does not provide any further information on the identity of the proteins being collected. Considering the highly variable immunoreactivities of wheat, rye and barley proteins it is essential to know the exact composition of the GPT isolates, especially when trying to gain further insights into pathogenic cascades of CD, NCGS, and wheat allergies (Vader et al., 2002; Matsuo et al., 2005; Scherf et al., 2019). For example, wheat ATIs were only identified as triggers of innate immunity *via* the toll-like receptor 4 in NCGS, because they were co-purified within the ω-gliadin fraction (Junker et al., 2012). Therefore, it is crucial to identify the individual proteins within each GPT isolate and undertake relative quantitation of the highly abundant proteins by liquid chromatography–mass spectrometry (LC-MS/MS).

In the current fundamental study, LC-MS/MS analysis was applied to all isolated GPTs of wheat, rye, and barley to precisely determine the identities of the proteins in each isolate as well as their relative abundances to provide a detailed assessment of the molecular composition. A special focus was placed on the identification of known CD-immunoreactive and allergenic peptides and proteins.

## Material and Methods

### Material

All chemicals and solvents were at least HPLC or LC-MS grade. Formic acid (FA), ammonium bicarbonate (Ambic), dithiothreitol (DTT), and iodoacetamide (IAM), were purchased from Sigma-Aldrich (Sydney, NSW, Australia). Trypsin (sequencing grade, V511A; specific activity: 15,282 units/mg) and chymotrypsin (sequencing grade, V106A; specific activity: at least 70 units/mg by N-benzoyl-L-tyrosine ethyl ester assay) were purchased from Promega (Sydney, NSW, Australia).

### Grain Samples

Grains of wheat [cultivar (cv.) Akteur, harvest year 2011, I.G. Pflanzenzucht, Munich, Germany], rye (cv. Visello, harvest year 2013, KWS Lochow, Bergen, Germany), and barley (cv. Marthe, harvest year 2009, Nordsaat Saatzucht, Langenstein, Germany) grown in Germany were milled into white flour using a Quadrumat Junior mill (Brabender, Duisburg, Germany). Subsequently, the flours were sieved to a particle size of 200 µm and allowed to rest for 2 weeks. The choice of these cultivars was based on production shares in Germany for conventional farming to ensure that these cultivars were of economic relevance and, therefore, deemed to be representative for each grain.

### Analysis of Moisture and Crude Protein Contents

The determination of moisture and crude protein (CP) contents (conversion factor N × 5.7) was carried out according to International Association for Cereal Science and Technology Standards 110/1 and 167.

### Preparation of Gluten Protein Types

The α-gliadins, γ-gliadins, ω1,2-gliadins, ω5-gliadins, HMW-GS and LMW-GS of wheat, ω-secalins, HMW-secalins, γ-75k-secalins, and γ-40k-secalins of rye, and B-hordeins, C-hordeins, D-hordeins, and γ-hordeins were isolated by modified Osborne fractionation and preparative RP-HPLC (Schalk et al., 2017) from the flours after a maximum of 6 weeks storage after milling in the respective year. The flours of wheat, rye, and barley (4 × 50 g) were extracted step-wise three times each with 200 ml salt solution (0.4 mol/l NaCl with 0.067 mol/l Na_2_HPO_4_/KH_2_PO_4_, pH 7.6) for 10 min at 22°C, centrifuged and the supernatant containing albumins/globulins was discarded. The sediments were extracted with ethanol/water (60/40, v/v) (3 × 200 ml) for 10 min at 22°C to obtain the prolamin fractions. For the glutelins, the resulting sediments were extracted three times each with 200 ml 2-propanol/water (50/50, v/v)/0.1 mol/l Tris-HCl, pH 7.5, containing 2 mol/l (w/v) urea and 0.06 mol/l (w/v) DTT for 30 min at 60°C under nitrogen. The supernatants of each prolamin and glutelin fraction were combined, concentrated, lyophilized and stored at -20°C until use. This whole extraction procedure was performed on four independent batches to give enough material for further analyses.

For preparative RP-HPLC, the wheat, rye, and barley prolamin fractions (200 mg) were dissolved in 10 ml ethanol/water and the glutelin fractions (1,000 mg) in 10 ml of the glutelin extraction solution. The solutions were filtered (0.45 µm) and separated on a Jasco HPLC (Jasco, Gross-Umstadt, Germany) according to their retention times, collected from several runs, pooled and lyophilized as described previously (Schalk et al., 2017). The isolated GPTs were again stored at -20°C until use. Long-term experience with storage of the Prolamin Working Group-gliadin reference material (Van Eckert et al., 2006) in our laboratory since its isolation in the early 2000s indicates that protein isolates are stable for several years or even decades when kept frozen at -20°C or, ideally, at -80°C.

### Enzymatic Cleavage of GPTs

The GPT hydrolysates were prepared as reported in Colgrave et al. (2016a; 2016b). Briefly, each GPT (n = 3) was dissolved in 50 mmol/l Ambic buffer with a concentration of 2 mg/ml and applied to a 10 kDa molecular weight cutoff filter (Millipore, Australia). The GPT solutions were washed with washing solution (2 × 100 µl; 8 mol/l urea; 100 mmol/l Tris-HCl; pH 8.5) and the filters were centrifuged. For reduction, DTT solution (10 mmol/l) was added; the filters were incubated for 40 min at room temperature and then centrifuged. For cysteine alkylation, 100 µl of IAM solution (25 mmol/l; in 8 mol/l urea; 100 mmol/l Tris-HCl) was added and the solution was incubated at room temperature in the dark for 20 min. The filters were centrifuged and washing solution was added (2 × 100 µl). To exchange the buffer, two times 200 µl of Ambic buffer was added and centrifuged. The 10 kDa filters were transferred to fresh centrifuge tubes, the digestion enzyme (trypsin or chymotrypsin: 200 µl; 250 µg/ml in 50 mmol/l Ambic; 1 mmol/l CaCl_2_; enzyme/substrate ratio of 1/4 (w/w); respectively) was added, and the mixture was incubated overnight at 37°C. The filtrates with the enzymatically cleaved peptides were collected by centrifugation, the filters were washed again with 200 µL of Ambic, and the filtrates and the washing solution were combined separately for each replicate and lyophilized. For LC-MS/MS analysis the peptides were resuspended in 100 µl 1% FA.

### Undirected LC-MS/MS Analysis

Aliquots (5 µl) of each GPT replicate were pooled for analysis. The LC-MS/MS analysis was performed on an Ekspert nanoLC415 (Eksigent, Dublin, CA, United States) directly coupled to a TripleTOF 6600 MS (SCIEX, Redwood City, CA, United States) with the following parameters: Trap column: ChromXP C18 (3 µm, 12 nm, 10 × 0.3 mm); flow rate: 10 µl/min solvent A; 5 min; column: ChromXP C18 (3 µm, 12 nm, 150 mm × 0.3 mm); flow rate: 5 µl/min; solvents: (A) 5% DMSO, 0.1% FA, 94.9% water; (B) 5% DMSO, 0.1% FA, 90% acetonitrile, 4.9% water; linear gradient from 3 to 25% solvent B over 68 min, followed by a second linear step from 25–35% solvent B over 5 min, followed by a third linear step from 35–80% B over 2 min; a 3 min hold at 80% B; return to 3% B over 1 min; 8 min of re-equilibration; injection volume: 2 µl. DMSO was added as it enhances ionization and increases the signal-to-noise ratio (Hahne et al., 2013). The eluent from the HPLC was directly coupled to the DuoSpray source of the TripleTOF 6600 MS. The MS settings were as follows: Ion spray voltage: 5,500 V; curtain gas: 138 kPa (20 psi); ion source gas 1 and 2 (GS1 and GS2): 103 and 138 kPa (15 and 20 psi); heated interface temperature: 100°C. The MS was operated in the information-dependent acquisition (IDA) mode. The IDA method consisted of a high-resolution time-of-flight-MS survey scan followed by 30 MS/MS scans, each with an accumulation time of 40 ms. The mass-to-charge (*m/z*) range of the acquisition of the MS1 spectra in positive ion mode was 400–1,250 with a 0.25 s accumulation time. MS2 spectra were acquired on precursor ions that exceeded 150 counts/s with charge states 2+ to 5+ and over the mass range of *m/z* 100–1,500 using the manufacturer’s rolling collision energy based on the size and charge of the precursor ion and a collision energy spread of 5 V for optimum peptide fragmentation. Analysis was carried out with dynamic ion exclusion of precursor ions with a 15 s interval after one occurrence and a mass tolerance of 100 ppm, and peaks within 6 Da of the precursor mass were excluded.

### Data Analysis for Protein Identification

For protein identification, the SCIEX.wiff raw files were directly used as input in the ProteinPilot 5.0 software (SCIEX) with the Paragon algorithm (Shilov et al., 2007). The raw data were searched against a database comprising UniProtKB-*Poaceae* proteins (https://www.uniprot.org; version 2018/02) appended with cRAP (http://www.thegpm.org/crap/), the common repository of adventitious proteins (1,601,923 sequences). The settings used were: IAM as the alkylating agent; trypsin, chymotrypsin, or no enzyme as the cleavage enzyme. ProteinPilot automatically considers enzyme cleavage specificity rules and all UniMod modifications, including e.g., oxidation of methionine and deamidation of asparagine and glutamine, and uses a probability-based approach that considers sample treatment conditions. A 1% global false discovery rate (FDR) was applied for the protein identifications. The detected proteins were classified according to Dupont et al. (2011) into the following groups: gluten proteins, ATIs, globulins, β-amylase, other enzymes, farinins, serpins, grain softness proteins and puroindolines (GSPs+PINs), avenin-like proteins, other inhibitors, uncharacterized proteins (name of entries in the database UniProtKB) and others. The group “others” contains all identified proteins, which could not be assigned to any of the aforementioned groups. All proteins identified as “uncharacterized” and “predicted” were manually reviewed using the basic local alignment search tool (BLAST) (Altschul et al., 1990) on the UniProtKB webpage with the target database UniProtKB reference proteomes plus SwissProt (parameters: identity >70%, except for hits with names of a group or from the subfamily *Pooideae*). Due to the challenge of having different terms and often uncurated and incomplete protein sequences in the UniProtKB *Poaceae* database, the protein names for gluten proteins were summarized in the group “gluten proteins”, which comprise gliadins, glutelins, glutenins and prolamins for wheat, secalins, glutelins, glutenins and prolamins for rye and hordeins, glutelins, glutenins and prolamins for barley. By means of the rank for the specified protein given by the Paragon algorithm in ProteinPilot, the detected proteins are sorted relative to all other ones. The proportion in each different group was calculated as the number of identified proteins per group multiplied by the number of distinct peptides with a >95% confidence level by which these proteins were identified to have a weighting factor for the rank of the specific protein relative to all other proteins

### Preparation of the Multiple Reaction Monitoring Methods Using Skyline

Within each GPT, the identified proteins were selected according to the following parameters: belonging to the family *Poaceae*, the subfamily *Pooideae* and to gluten; 1% global FDR; confidence score > 99% and unused score > 2.0. The manually curated FASTA files list and the results of the undirected LC-MS/MS experiments were imported into Skyline (version 4.2.0.19072). Multiple reaction monitoring (MRM) transitions were determined for each peptide predicted with precursor ion (Q1) with *m/z* (50–1,500) and charge (2+; 3+) and fragment ion (Q3) *m/z* values using the data collected in the undirected LC-MS/MS experiments (Colgrave et al., 2012). Up to six transitions were used in the preliminary analyses and the MRM transitions were refined and the top four MRM transitions were selected per peptide for use in the final method. In the subsequent experiments scheduled MRM transitions were used for analysis in triplicate.

### Multiple Reaction Monitoring Mass Spectrometry for Relative Protein Quantitation

Scheduled MRM experiments were used for quantitation of the reduced and alkylated tryptic and chymotryptic peptides of each GPT in triplicate, respectively. The LC-MS/MS analysis was performed on an UHPLC system (Shimadzu Nexera, Sydney, Australia) directly coupled to a QTRAP 6500 mass spectrometer (SCIEX). The cycle time was set to 0.3 s, and the MRM transitions were scheduled to be monitored within 60 s of their expected retention time (± 30 s) (Colgrave et al., 2017a).

### Relative Protein Quantitation

The peaks were integrated using Skyline. The relative quantitation of the proteins within each GPT was performed by using the “best flyer methodology” (Ludwig et al., 2012), in which the peak areas of four transitions of one peptide (average of three replicates) were summarized. One peptide is used to represent one protein and the values of the peak area of each peptide were assigned to the respective protein. The datasets from the tryptic and chymotryptic digests were combined by removing the duplicate protein with the lower value. Then, the areas of all proteins from the same category according to their UniProtKB accession were summarized. The calculations were done in Microsoft Excel and the graphical images were done in Origin (version 2018b (9.55), OriginLab Northampton, MA, USA).

## Results

### General Characterization of Gluten Protein Types

The moisture contents of the flours were 14.59 ± 0.01% for wheat, 11.42 ± 0.01% for rye and 12.09 ± 0.06% for barley. The contents of CP, albumin/globulin, prolamin, and glutenin fractions in the flours are given in **Table S1**. **Table S2** lists the CP contents of the GPTs isolated from wheat, rye and barley flours and the proportions of each GPT within total gluten. The Osborne fraction values are based on flour weight; the proportions of GPTs are based on total gluten content (Lexhaller et al., 2016; Lexhaller et al., 2017). The results corresponded well to those reported previously (Gellrich et al., 2003; Kerpes et al., 2016; Schalk et al., 2017)

### Identification of Protein Groups in the Gluten Protein Types

**Figure 1:**
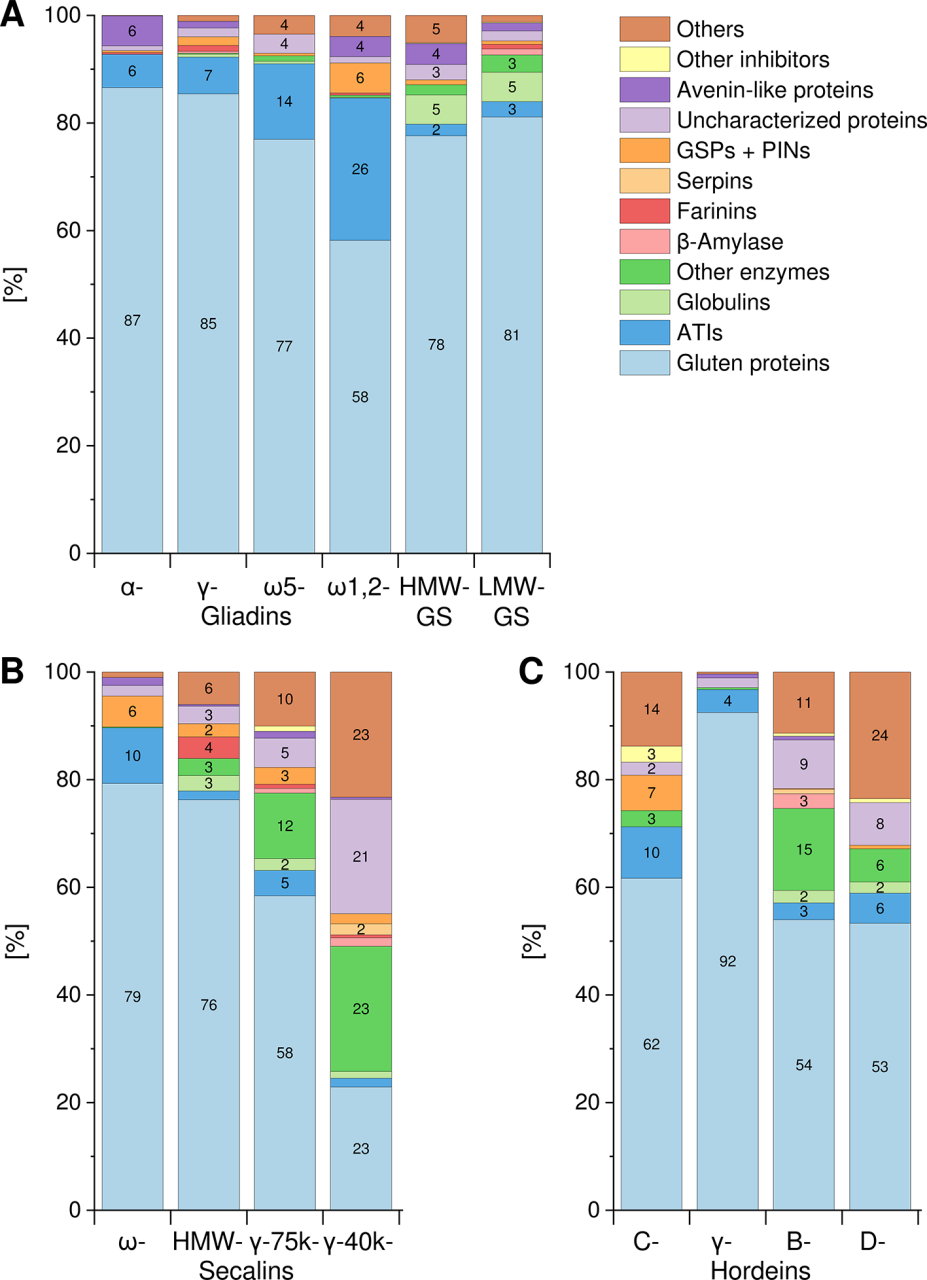
Composition and proportions of proteins in each GPT. Classification of identified proteins into the following groups for wheat **(A)**, rye **(B)**, and barley **(C)** gluten protein types: gluten proteins, α-amylase/trypsin-inhibitors (ATIs), globulins, other enzymes, β-amylase, farinins, serpins, grain softness proteins, and puroindolines (GSPs+PINs), uncharacterized proteins, avenin-like proteins, other inhibitors, and others. When a group is missing in individual GPT, no proteins were identified. Groups without number represent less than 2%. GS, glutenin subunits; HMW, high-molecular-weight; LMW, low-molecular-weight.

The Osborne fractions (prolamins and glutelins) extracted from the flours were separated into the GPTs by preparative RP-HPLC. These purified GPTs were reduced, alkylated and subjected to tryptic (T) and chymotryptic (C) hydrolysis, respectively. The GPT hydrolysates were analyzed by LC-MS/MS to identify the complete suite of proteins present in each GPT. Proteins with identical sequences were used once. For each GPT, the suite of proteins identified after tryptic digest (**Table S3**) and after chymotryptic digest (**Table S4**) were recorded. All proteins originally identified as “uncharacterized” or “predicted” were manually searched again using the BLAST tool available from the UniProtKB webpage. According to the data of the undirected LC-MS/MS experiments, **Figure 1** shows the qualitative composition and proportion of the proteins in each GPT.

#### Wheat

A similar composition with mainly gluten proteins (87% and 85%, respectively) and 6–7% ATIs was detected in the α- and γ-gliadin-GPTs. The ω5-gliadin-GPT was composed of 77% gluten proteins and 14% ATIs, whereas the ω1,2-gliadin-GPT contained about 58% gluten proteins, 26% ATIs and 6% GSPs+PINs. HMW- and LMW-GS-GPTs showed a comparable composition with about 78% or 81% gluten proteins, respectively (**Figure 1A**).

#### Rye

The ω-secalin-GPT consisted of 79% gluten proteins, 10% ATIs, and 6% GSPs+PINs. In the HMW-secalin-GPT, 4% farinins, 3% other enzymes, and 3% globulins were identified besides 76% gluten proteins. The γ-75k-secalin-GPT was composed of 58% gluten proteins, 5% ATIs and more than 10% other enzymes. The composition of the γ-40k-secalin-GPT included only 23% gluten proteins, 23% other enzymes and about 23% others. It should be noted that 21% of the identified proteins were uncharacterized ones (**Figure 1B**).

#### Barley

The C-hordein-GPT consisted mainly of 62% gluten proteins, 10% ATIs and 7% GSPs+PINs. The γ-hordein-GPT was composed of over 92% gluten proteins and 4% ATIs and the residual groups amounted only to 4% altogether. The compositions of B- and D-hordein-GPTs were similar, but the B-hordein-GPT had a greater diversity of enzymes (15% in total) and contained 11% uncharacterized proteins. In the D-hordein-GPT (**Figure 1C**) high proportions of other proteins (24%) were present.

### Identification of Single Proteins in the Gluten Protein Types

**Tables S3** and **S4** list all identified proteins with their UniProtKB accession number, name, organism, rank, score, sequence coverage and number of identified peptides. As an overview of the qualitative data, the three proteins with the highest ranks identified in the tryptic (**Table 2**) and in the chymotryptic (**Table 3**) hydrolysates, respectively, of each GPT according to the rank are summarized. The rank of each specified protein is relative to all identified proteins in the fraction and contaminant proteins, such as the proteases used and/or keratins from sample preparation were excluded.

**Table 2:** High-scoring proteins (top 3) identified in each gluten protein type (GPT) after tryptic cleavage.

GPT	Rank^a^	UniProtKB accession	UniProtKB name	Score^b^	Peptides
**α-gliadins**	1	R9XUM8	Alpha-gliadin	30.03	96
	2	B2Y2Q4	Low molecular weight GS	17.12	55
	3	P17314	Alpha amylase inhibitor CM3	13.52	18
**γ-gliadins**	1	Q41553	HMW-GS Ax2	27.66	31
	3	I3XHQ1	LMW-9	24.08	38
	4	W6AX70	HMW-GS	20.79	25
**ω5-gliadins**	1	Q41553	HMW-GS Ax2	52.89	56
	4	V9TRL3	HMW-GS 1Dy	21.91	24
	6	P10388	HMW-GS Dx5	16.73	17
**ω1,2-gliadins**	5	G9I1R7	Alpha-gliadin Gli-M2	24.59	29
	7	C8CAI4	Dimeric alpha-amylase inhibitor	24.10	28
	8	B9VRI3	Alpha-amylase inhibitor CM16	20.01	28
**HMW-GS**	1	W6AX70	HMW subunit	71.28	113
	2	A0A060MZP1	HMW subunit	54.59	123
	3	P10388	HMW subunit	41.05	97
**LMW-GS**	1	M7ZK46	Seed storage globulin 1	26.70	32
	2	A0A060MZP1	HMW glutenin subunit	18.51	31
	4	D6RVY4	LMW glutenin subunit	16.56	73
**ω-secalins**	2	A0A159KI56	Omega-secalin	23.75	79
	5	Q7M220	Trypsin inhibitor	16.31	22
	6	W6AW98	HMW-GS x	14.17	19
**HMW-secalins**	1	W6AW92	HMW-GS y	39.73	221
	2	Q93WF0	HMW-GS x	30.31	109
	4	W8NKZ9	B-type farinin protein	10.70	27
**γ-75k-secalins**	1	E5KZQ2	75k gamma secalin	53.31	165
	2	B9A8E2	Protein disulfide-isomerase	11.52	30
	3	Q9ZSR6	Heat shock protein HSP26^c^	2.60	12
**γ-40k-secalins**	1	A0A1D5U769	Sucrose synthase	28.53	25
	2	M8ASF1	Actin-2^d^	27.76	23
	4	H8Y0K4	Gamma prolamin	24.32	83
**C-hordeins**	2	Q84LE9	D-hordein	17.38	32
	3	Q5IUH1	Hordoindoline-B 1	10.09	6
	4	Q41518	RNA-binding protein^e^	8.14	7
**γ-hordeins**	2	I6TMW4	B3-hordein	33.90	60
	3	P06470	B1-hordein	16.30	64
	4	P80198	Gamma-hordein-3	13.20	16
**B-hordeins**	1	F2D284	Protein disulfide-isomerase	31.05	22
	2	M7ZK46	12S seed storage globulin 1^f^	22.68	17
	3	I6TMW4	B3-hordein	21.90	102
**D-hordeins**	1	I6TRS8	D-hordein	35.20	209
	3	Q41350	Osmotin-like protein^g^	13.57	8
	4	Q41518	RNA-binding protein^h^	11.63	10

^a^The rank of the specified protein is relative to all other proteins in the list of detected proteins, ^b^Unused ProtScore, defined as a measure of the protein confidence for a detected protein, calculated from the peptide confidence for peptides from spectra that are not already completely “used” by higher scoring winning proteins, thus reflecting the amount of total, unique peptide evidence related to a given protein, ^c^after BLAST search (identified as uncharacterized protein: R7W8L3), ^d^after BLAST search (identified as uncharacterized protein: W5AHI2), ^e^after BLAST search (identified as predicted protein: F2CR90), ^f^after BLAST search (identified as predicted protein: F2E9N0), ^g^after BLAST search (identified as predicted protein: F2DZW3), ^h^after BLAST search (identified as predicted protein: F2CR90).

**Table 3:** High-scoring proteins (top 3) identified in each gluten protein type (GPT) after chymotryptic cleavage.

GPT	Rank^a^	UniProtKB accession	UniProtKB name	Score^b^	Peptides
**α-gliadins**	1	J7I026	Alpha-gliadin	19.29	34
	2	A0A0U2P410	Low molecular weight GS	14.37	19
	3	I3XHQ1	Low molecular weight GS	8.51	7
**γ-gliadins**	2	Q9XGF0	Low molecular weight GS	4.97	4
	3	B6UKM7	Gamma gliadin	2.97	1
	4	P94021	LMM glutenin 2 (Fragment)	2.92	1
**ω5-gliadins**	1	D6RVY4	LMW-GS (Fragment)	6.66	10
	2	P10387	HMW-GS Dy10	6.45	9
	4	Q41553	HMW-GS Ax2	2.49	4
**ω1,2-gliadins**	1	A0A060N0S6	Omega-gliadin	17.11	89
	2	P10388	HMW-GS Dx5	4.84	6
	3	P10385	Low molecular glutenin subunit^c^	4.53	3
**HMW-GS**	1	C0SUC3	HMW glutenin subunit x5	27.39	39
	2	P10387	Glutenin, HMW subunit Dy10	13.58	44
	3	Q03872	HMW subunit 1Ax1	12.04	30
**LMW-GS**	1	D6RVY4	Low molecular glutenin subunit	13.32	41
	2	I3XHQ1	LMW glutenin subunit LMW-9	12.30	19
	3	A0A0S2GJT4	LMW glutenin subunit	9.11	24
**ω-secalins**	1	A0A159KI90	Omega-secalin	10.57	68
	2	W6W98	HMW-GS x	4.45	8
**HMW-secalins**	1	W6AW92	HMW-GS y	17.33	38
	2	Q93WF0	HMW-GS x	14.61	58
	3	Q43639	Sec1	3.96	12
**γ-75k-secalins**	1	P52589	Protein disulfide-isomerase	8.38	4
	2	Q94IL2	HMW-GS x	5.47	3
	3	E5KZQ1	75k gamma-secalin	4.68	49
**γ-40k-secalins**	1	W5IA32	Formate dehydrogenase	7.41	3
	2	K3ZAI0	Uncharacterized protein	7.31	8
	3	W4ZSH7	Uncharacterized protein	5.98	5
**C-hordeins**	1	P06472	C-hordein^d^	7.44	19
**γ-hordeins**	2	P06470	B1-hordein^e^	3.95	9
	4	A0A287Q402	Uncharacterized protein	2.00	1
**B-hordeins**	1	Q84LE9	D-hordein	13.59	21
	2	P06470	B1-hordein	11.49	38
	3	F2D284	Protein disulfide-isomerase	9.79	7
**D-hordeins**	1	I6SW34	D-hordein	33.73	99
	2	P07597	Non-specific lipid-transfer protein	5.07	4
	3	P02864	C-hordein^f^	4.30	2

^a^The rank of the specified protein is relative to all other proteins in the list of detected proteins, ^b^Unused ProtScore, defined as a measure of the protein confidence for a detected protein, calculated from the peptide confidence for peptides from spectra that are not already completely “used” by higher scoring winning proteins, thus reflecting the amount of total, unique peptide evidence related to a given protein, ^c^after BLAST search (identified as uncharacterized protein: T1LG74), ^d^after BLAST search (identified as uncharacterized protein: A0A287EIM7), ^e^after BLAST search (identified as uncharacterized protein: A0A287EFG2), ^f^after BLAST search (identified as uncharacterized protein: A0A287EEX5).

#### Wheat

The high-scoring proteins detected in the tryptic hydrolysates of the α-gliadin-GPT and the γ-gliadin-GPT represented gluten proteins, except one α-amylase-inhibitor (**Table 2**). The top-ranked proteins often did not match those of the corresponding protein type, whereas the matching proteins appeared at lower ranks, e.g., γ-gliadins (D0ES80; H8Y0P9) at ranks five and seven in the γ-gliadin-GPT with similar scores and peptide numbers. The chymotryptic hydrolysates (**Table 3**) showed similar compositions. The tryptic hydrolysate of the ω5-gliadin-GPT contained mainly HMW-GS proteins, but an ω-gliadin (A0A0B5J8A9) was identified based on eight peptides at rank 12. Surprisingly, no ω-gliadin was identified in the chymotryptic hydrolysate of the ω5-gliadin-GPT. The tryptic hydrolysate of the ω1,2-gliadin-GPT was composed of different types of proteins representing the two main groups of this GPT (**Figure 1A**). The chymotryptic hydrolysate contained an ω-gliadin protein (A0A060N0S6) at rank 1 with by far the highest score and the most identified peptides (89). In the tryptic and chymotryptic hydrolysates of the HMW-GS-GPT the highest ranked proteins were HMW-GS. The high-scoring proteins in the tryptic LMW-GS-GPT were the 12S seed storage globulin (M7ZK46), which belongs to the cupin super-family with nutrient reservoir activity (Dunwell, 1998) and one LMW-GS, which was identified with the highest number of peptides. These proteins represent the main group, gluten proteins, and the second main group in this GPT, the globulins (**Figure 1A**). Globulins are known to polymerize *via* interchain disulfide bonds and may thus appear in the high-molecular-weight group (Vensel et al., 2014).

#### Rye

The three proteins with the highest scores in the tryptic ω-secalin-GPT hydrolysate (**Table 2**) were an ω-secalin, a trypsin inhibitor and a HMW-GS, which represent the two main groups of the ω-secalin-GPT in **Figure 1B**. Only two proteins passing the 1% FDR threshold were identified in the chymotryptic hydrolysate of the ω-secalin-GPT (**Table 3**). In the tryptic and chymotryptic hydrolysates of the HMW-secalin-GPT, the highest ranked proteins were a HMW-secalin (Q93WF0; rank 2) and a wheat HMW-GS protein (W6AW92; rank 1), which is, however, very similar to the HMW-secalin protein D3XQB8 (95.8% identity). The tryptic hydrolysate of the γ-75k-secalin-GPT consisted mainly of the 75k gamma secalin protein E5KZQ2. The high scoring proteins represent the three main groups in the γ-75k-secalin-GPT (**Figure 1B**). Another 75k γ-secalin protein (E5KZQ6) was also identified with a high number of peptides, but a lower score. In the chymotryptic hydrolysate, the protein identified with the most peptides (49) was the 75k γ-secalin E5KZQ1 at rank 3. In case of the γ-40k-secalin-GPT, only one γ-prolamin protein was identified in the tryptic hydrolysate at rank 3. A sucrose synthase and an uncharacterized protein (W5AHI2) ranked first and second, respectively. The BLAST search identified an actin-2 protein (M8ASF1) with 100% identity to this uncharacterized protein. Uncharacterized proteins represented one of the largest groups in the γ-40k-secalin-GPT (**Figure 1B**), probably due to missing reference protein sequences. The chymotryptic hydrolysate showed a similar proportion with a formate dehydrogenase and two uncharacterized proteins as the three high-scoring proteins.

#### Barley

The high-scoring proteins detected in the tryptic hydrolysate of the C-hordein-GPT (**Table 2**) corresponded to the three main groups of this GPT, the gluten proteins, the group of others and the group of GSPs+PINs (**Figure 1C**). A C-hordein (Q40055) was identified at rank 23. An uncharacterized protein of *Hordeum vulgare* subsp. *vulgare* (A0A287EIM7) sharing 99.0% homology with the C-hordein (P06472) was present in the chymotryptic hydrolysate of the C-hordein-GPT (**Table 3**). Two B-hordeins and the previously reported γ3-hordein (P80198) (Colgrave et al., 2012) were detected with a high number of peptides in the tryptic hydrolysate of the γ-hordein-GPT. Only two uncharacterized proteins from *Hordeum vulgare* subsp. *vulgare* were identified in the chymotryptic γ-hordein-GPT hydrolysate. The highest ranked protein was identified as a B1-hordein (P06470) with an identity of 94.6% after the BLAST search. The tryptic and chymotryptic hydrolysates of the B-hordein-GPT contained the B3-hordein I6TMW4 with 102 peptides and the two other B-hordeins with a high peptide number, B1-hordein (P06470) and B hordein (Q40026). D-hordein (I6TRS8, 209 peptides detected) was the highest ranking protein in the tryptic hydrolysate of the D-hordein-GPT. The D-hordein (I6SW34, 99 peptides) and an uncharacterized protein (A0A287EEX5, 2 peptides), which was identified as a C-hordein (P02864) with 50% identity were identified in the chymotryptic hydrolysate. Moreover, D-hordeins were detected in all other hordein GPTs with high sequence coverage.

The best three protein hits of each GPT are summarized in **Tables 2** and **3**, according to their ranking of identification. The total numbers of gluten proteins identified using either trypsin or chymotrypsin are presented in **Table 4**. The numbers of identified proteins were between 2- to 10-fold higher in all GPT hydrolysates using the so-called gold standard proteolytic enzyme trypsin as compared to chymotrypsin. The numbers of identified gluten proteins were 2- to 8-fold higher in the tryptic hydrolysates, except for HMW-GS and LMW-GS. Chymotrypsin revealed as many gluten proteins as trypsin for HMW-GS and more gluten proteins were identified in the chymotryptic hydrolysate of LMW-GS than with trypsin. The total numbers of identified proteins differed from 24 for the γ-hordeins up to 317 for the γ-40k-secalins in the tryptic hydrolysates and from 4 (ω5-gliadins) to 58 (γ-40k-secalins) in the chymotryptic hydrolysates. The ratio of the numbers of all identified proteins to the numbers of identified gluten proteins ranged from 2 for α-gliadins up to 29 for γ-40k-secalins in the tryptic hydrolysates and from 1 for α-gliadins, ω5-gliadins and ω1,2-gliadins to 19 for γ-40k-secalins in the chymotryptic hydrolysates. It should be noted that 18 gluten proteins, but no GPT-specific proteins were identified (73 proteins in total) in the tryptic digest of the ω1,2-gliadin-GPT. In contrast, only seven gluten proteins were identified in the chymotryptic hydrolysate, but among which three of them were ω-gliadin proteins. The same findings were observed for the LMW-GS, for which 22 LMW-GS proteins of 27 gluten proteins were identified in the chymotryptic hydrolysate, but only 2 LMW-GS-proteins within 20 gluten proteins in the tryptic hydrolysate. For the hordeins, the data shows that the enrichment is more specific and that the trypsin data for these GPTs is misleading, because in the chymotryptic hydrolysates less gluten proteins were identified, but more of them corresponded to their appropriate GPT. When looking at the other GPTs, more GPT-specific proteins were identified in the tryptic than in the chymotryptic hydrolysates.

**Table 4:** Total numbers of identified proteins, gluten proteins, and gluten protein type (GPT)-specific proteins in each GPT digested with trypsin or chymotrypsin, respectively.

GPT	Tryptic			Chymotryptic			Total gluten proteins^d^
	Proteins^a^	Gluten proteins^b^	GPT-specific proteins^c^	Proteins^a^	Gluten proteins^b^	GPT-specific proteins^c^	
α-gliadins	48	20	7	11	11	2	31
γ-gliadins	61	21	4	19	6	2	27
ω5-gliadins	37	8	1	4	3	0	11
ω1,2-gliadins	73	19	0	8	7	3	26
HMW-GS	117	19	10	37	16	6	35
LMW-GS	78	20	2	52	27	22	47
ω-secalins	56	10	6	10	2	1	12
HMW-secalins	96	10	3	18	6	1	16
γ-75k-secalins	244	13	3	43	3	1	16
γ-40k-secalins	317	11	2	58	3	1	14
C-hordeins	37	7	1	11	1	1	8
γ-hordeins	24	8	1	7	1	0	9
B-hordeins	152	7	3	15	3	2	10
D-hordeins	130	8	1	24	3	1	11

^a^Global FDR = 1%; ^b^proteins from all Poaceae included; ^c^only proteins from appropriate GPTs included; ^d^numbers of gluten proteins identified in tryptic and chymotryptic hydrolysates summed without duplicates.

### Identification of Immunoreactive Proteins

Various gluten and non-gluten proteins of wheat, rye and barley have been identified as triggers of adverse reactions. The proteomic characterization of the GPTs also provided an insight into the presence of immunoreactive proteins. All identified proteins of the GPTs were searched for the UniProtKB accession based on the allergen code of the World Health Organization/International Union of Immunological Societies and for the name of the immunoreactive proteins. The identified allergens with their allergen code, molecular weight and identification parameters are shown in **Table 5**. Some of the allergens were identified only in one GPT with a small number of peptides (profilin in the LMW-GS-GPT or serpin in the γ-40k-secalin-GPT), but especially ATIs and gluten proteins were very abundant and present in more than one GPT. However, it should be noted that most of the allergens were enriched in one GPT. The WDEIA allergen tri a 19 “ω5-gliadin” was identified only in the appropriate GPT.

**Table 5:** Identified allergens of wheat (Tri), rye (Sec), and barley (Hor), their allergen code according to the World Health Organization/International Union of Immunological Societies allergen nomenclature, their UniProtKB accession number and name, the gluten protein type (GPT), in which they were identified and their identification parameters.

Allergen name	UniProtKB accession	Name	MW^a^ [kDa]	GPT	T/ C^b^	Score^c^	Peptides (>95%)
Tri a 12	D0PRB5	Profilin	14	LMW-GS	T	2.00	1
Tri a 15	P01083	α-Amylase-inhibitor 0.28	17	γ-40k-secalins	T	2.00	1
Tri a 19	A0A0B5J8A9	ω5-Gliadin	40	ω5-gliadins	T	8.00	8
Tri a 20	D0ES80	γ-Gliadin	34	γ-gliadins α-gliadins	T T	20.59 5.85	51 9
Tri a 21	I0IT55 P04727 I0IT62 Q41546 P04721	α-/β-Gliadin	34 36 38 36 30	α-gliadins γ-gliadins ω1,2-gliadins HMW-GS LMW-GS	T T T T T	2.27 2.71 2.00 2.00 4.38	77 10 26 7 11
Tri a 26	P10388	High molecular weight glutenin subunit Dx5	88	γ-gliadins ω5-gliadins ω1,2-gliadins HMW-GS	T T T T	3.23 16.73 6.24 41.05	6 17 24 97
	Q45R38	High molecular weight glutenin subunit Bx7	85	HMW-GS HMW-GS	T C	39.96 8.16	64 33
Tri a 28	P01085	α-Amylase-inhibitor 0.19	13	ω5-gliadins LMW-GS	T T	6.00 2.00	8 2
	Q5MD68	α-Amylase-inhibitor 0.19	13	ω1,2-gliadins	T	8.01	20
	P01084	α-Amylase-inhibitor 0.53	13	α-gliadins γ-gliadins	T T	8.78 3.94	6 4
Tri a 30	P17314	Tetrameric alpha-amylase inhibitor CM3	16	α-gliadins γ-gliadins ω1,2-gliadins HMW-GS	T T T T	13.52 10.00 8.31 2.03	18 5 4 1
Tri a 31	P46226^d^	Triosephosphate-isomerase	27	γ-75k-secalins γ-40k-secalins	T T	2.00 9.85	1 5
Tri a 32	Q6W8Q2	1-cys-peroxiredoxin		LMW-GS	T	2.47	1
Tri a 33	Q9ST57	Serpin		γ-40k-secalins	C	2.02	1
Tri a 34	C7C4X1	Glyceraldehyde-3-phosphate-dehydrogenase		γ-40k-secalins	C	2.02	1
Tri a 36	B2Y2Q4 I3XHQ1 Q9XGF0 Q8W3V1 A0A165R8I1 D6RVY4 R4JFB5 Q6PKM2	LMW glutenin subunit LMW glutenin subunit LMW-9 LMW GS group 11 type VI S-type LMW GS LMW GS LMW GS LMW GS	42	α-gliadins γ-gliadins ω5-gliadins ω1,2-gliadins LMW-GS HMW-GS ω-secalins γ-75k-secalins γ-hordeins	T T T T T T T T T	17.12 24.08 6.47 17.69 14.25 16.56 6.02 4.24 2.00	55 38 4 35 22 73 5 5 10
Tri a 37	Q9T0P1	Alpha purothionin	12	HMW-GS	T	2.00	2
Tri a 40	Q41540	Chloroform/methanol-soluble (CM) 17 protein [alpha amylase inhibitor]	16	ω1,2-gliadins HMW-GS LMW-GS	T T T	8.00 4.39 4.53	24 4 3
Tri a 44	A0A0G3F720	Endosperm transfer cell specific PR60 precursor		HMW-secalins	T	4.06	3
Sec c 38	Q9S8H2	Dimeric alpha-amylase/trypsin inhibitor	13.5	γ-75k-secalins γ-40k-secalins	T T	2.00 2.00	1 1
Hor v 15	P16968 P28041	Alpha-amylase inhibitor BMAI-1 precursor Alpha-amylase/trypsin inhibitor CMa	14.5	γ-75k-secalins γ-hordeins B-hordeins D-hordeins	T T T T	2.00 2.00 4.59 11.35	1 1 3 16
Hor v 17	P16098 U5NJ12	Beta-amylase	60 60	B-hordeins B-hordeins	T C	19.68 4.65	17 3
Hor v 20	I6TEV2 P80198	Gamma 3 hordein Gamma-hordein 3	34	C-hordeins γ-hordeins	T T	7.85 2.00	8 1

^a^Molecular weight according to UniProtKB accession, ^b^T, tryptic digest, C, chymotryptic digest, ^c^Unused ProtScore, defined as a measure of the protein confidence for a detected protein, calculated from the peptide confidence for peptides from spectra that are not already completely “used” by higher scoring winning proteins, thus reflecting the amount of total, unique peptide evidence related to a given protein, ^d^96% identity to Q9FS79 Triticum aestivum.

Beside the shown exemplary allergens, many identified proteins contained peptides with known CD-active sequences. Immunoreactive peptides carrying known, non-deamidated peptide-binding motifs of gluten-specific T-cells are shown in **Table 6**. CD-active peptides were identified in all wheat GPTs, except ω5-gliadins. The list of T-cell epitopes according to Sollid et al. (2012) contains 31 entries that are reduced to 21 different motifs after reversal of deamidation and removal of duplicates. One of these motifs is specific to oats that were not studied, leaving 20 possible motifs. Of these, five epitopes were not identified (DQ2.5-glia-α3, DQ2.5-glia-γ4a, DQ2.5-glia-γ4b, DQ2.5-glia-γ4d, DQ8-glia- α1), but 15 motifs were detected, especially in the ω1,2-gliadin-, LMW-GS-, and HMW-GS-GPTs. The findings were comparable for the rye GPTs, where similar numbers of peptides were identified in the ω- and HMW-secalin-GPTs as in the γ-75k-secalin-GPT, with the exception of the γ-40k-secalin-GPT with just two epitopes. In the γ-, B-, and D-hordein-GPTs just one peptide-binding motif was detected, but six different peptides were identified in the C-hordein-GPT. The DQ2.5-glia-γ4c peptide-binding motif QQPQQPFPQ was detected in the ω1,2-, HMW-, and LMW-GS-GPTs, in all four rye GPTs and in the C-hordein-GPT. The DQ2.5-glia-γ5 motif QQPFPQQPQ was also identified in all rye GPTs and in the HMW-GS-GPT. The most frequently detected peptide-binding motif was PFPQPQQPF (DQ2.5-glia-ω1, DQ2.5-hor-1, DQ2.5-sec-1).

**Table 6:** Celiac disease relevant T-cell epitopes (nomenclature according to Sollid et al., 2012) identified in the gluten protein types, respectively.

Epitope	Peptide-binding motif	Reference	Gluten protein type^a^
DQ2.5-glia-α1a	PFPQPQLPY	Arentz-Hansen et al., 2000	α-gliadins LMW-GS HMW-GS
DQ2.5-glia-α1b	PYPQPQLPY	Arentz-Hansen et al., 2002	LMW-GS
DQ2.5-glia-α2	PQPQLPYPQ	Arentz-Hansen et al., 2000	LMW-GS HMW-GS
DQ2.5-glia- γ1	PQQSFPQQQ	Sjöström et al., 1998	ω-secalins
DQ2.5-glia- γ2	IQPQQPAQL	Qiao et al., 2005; Vader et al., 2002	α-gliadins γ-gliadins LMW-GS
DQ2.5-glia- γ3	QQPQQPYPQ	Arentz-Hansen et al., 2002	ω-secalins γ-75k-secalins
DQ2.5-glia- γ4c	QQPQQPFPQ	Arentz-Hansen et al., 2002	ω1,2-gliadins HMW-GS LMW-GS ω-secalins HMW-secalins γ-75k-secalins γ-40k-secalins C-hordeins
DQ2.5-glia- γ5	QQPFPQQPQ	Arentz-Hansen et al., 2002	HMW-GS ω-secalins HMW-secalins γ-75k-secalins γ-40k-secalins C-hordeins
DQ2.5-glia-ω1 DQ2.5-hor-1 DQ2.5-sec-1	PFPQPQQPF	Tye-Din et al., 2010; Vader et al., 2003	ω1,2-gliadins HMW-GS ω-secalins HMW-secalins γ-75k-secalins C-hordeins γ-hordeins
DQ2.5-glia- ω2	PQPQQPFPW	Tye-Din et al., 2010	ω1,2-gliadins HMW-GS C-hordeins
DQ2.5-glut-L2	FSQQQQSPF	Vader et al., 2002; Stepniak et al., 2005	LMW-GS
DQ2.5-hor-2 DQ2.5-sec-2	PQPQQPFPQ	Vader et al., 2003	γ-75k-secalins
DQ2.5-hor-3	PIPQQPQPY	Tye-Din et al., 2010	γ-hordeins B-hordeins D-hordeins
DQ2.2-glut-L1	PFSQQQQPV	Bodd et al., 2012	α-gliadins HMW-GS
DQ8-glut-H1	QGYYPTSPQ	van de Wal et al., 1999	γ-gliadins ω1,2-gliadins HMW-GS LMW-GS HMW-secalins

^a^Gluten protein types in which the peptides were identified.

### Relative Quantitation of Proteins Within Gluten Protein Types

**Figure 2:**
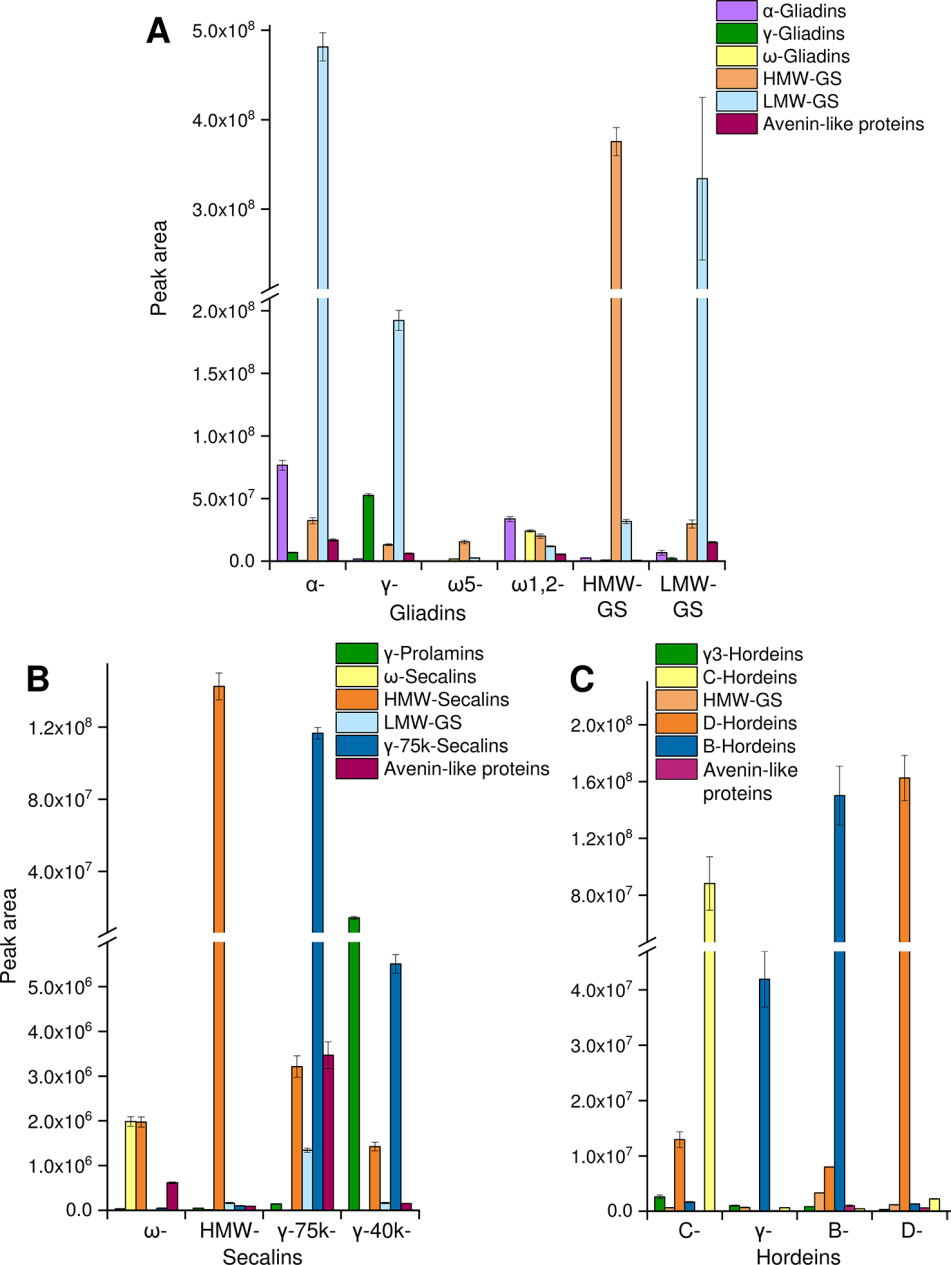
Relative protein quantification in GPTs. The summed peak areas of selected tryptic and chymotryptic peptides of the most abundant proteins representing protein groups in individual GPTs: peak areas of peptides representing α-gliadins, γ-gliadins, ω-gliadins, HMW-GS, LMW-GS, and avenin-like proteins in the GPTs of wheat **(A)**, peak areas of peptides representing γ-prolamins, ω-secalins, HMW-secalins, LMW-GS, γ-75k-secalins, and avenin-like proteins in the GPTs of rye **(B)**, peak areas of peptides representing γ3-hordeins, HMW-GS, D-hordeins, B-hordeins, C-hordeins, and avenin-like proteins in the GPTs of barley **(C)**. Data is plotted as the mean ± standard deviation (n = 3).

The tryptic and chymotryptic GPT hydrolysates were then subjected to relative quantitation to monitor the relative abundance of the peptides. Only peptides of gluten-derived proteins were selected for the MRM analysis. According to the “best-flyer method” of Ludwig et al. (2012), the peak areas of the four most intense transitions of the best flying peptide per protein (TopPep1/TopTra4) were summed. The model TopPep1/TopTra4 was selected, because only one peptide was detected for many gluten proteins in the undirected LC-MS/MS experiments and it is indicated that this model is as reasonable and robust as the others. The peak areas cannot be compared between peptides, because the MS response is dependent on the amino acid sequence, but the peak areas of the same peptide may be compared between the GPTs. The peak areas of the peptides were summed according to their categories (**Figure 2**). To estimate the enrichment of each category in every GPT the peak areas of each category were converted to a percentage relative to the summed peak area of the respective category for ease of data comparison.

#### Wheat

For the wheat GPTs, the single proteins were grouped according to their UniProtKB names into the categories LMW-GS, α-, γ-, and ω-gliadins, HMW-GS and avenin-like proteins. LMW-GS constituted the main proportion in the appropriate LMW-GS-GPT, but they were also enriched in the α- and γ-gliadin-GPTs and were present in the other wheat GPTs (**Figure 2A**). Vice versa, a large share of α-gliadins was detected in the α-gliadin- (≈42% of total α-gliadins) and HMW-GS-GPT (≈40% of total α-gliadins). The percentages always refer to 100% of total protein type summed over all wheat, rye or barley GPTs, respectively, e.g., to 100% of total α-gliadins summed over all wheat GPTs. Smaller proportions of α-gliadins were detected in the ω1,2-, γ-gliadin-, and LMW-GS-GPTs. The γ-gliadins were detected in almost all GPTs, except the ω-gliadin-GPTs, but were noticeably enriched in the γ-gliadin-GPT (≈66% of total γ-gliadins). The ω-gliadins were present almost only in the ω1,2-gliadin-GPT (≈76% of total ω-gliadins). HMW-GS accounted for a small proportion in each wheat GPT, but the HMW-GS-GPT had the highest proportion of HMW-GS (≈77% of total HMW-GS), as expected. The ω5-gliadin-GPT showed low proportions of the analyzed proteins of HMW-GS, LMW-GS and ω-gliadins. The avenin-like proteins were present in small amounts in almost all wheat GPTs, except the ω5-gliadin-GPT. The technical variation was assessed by examining the mean (combining GPTs of wheat) coefficient of variation (CV) for each peptide with an overall average of 13% for the cleavage with trypsin and 12% for the cleavage with chymotrypsin.

#### Rye

For the rye GPTs, the proteins were categorized according to their UniProtKB names into γ-75k-secalins, γ-prolamins, HMW-secalins, ω-secalins, LMW-GS, and avenin-like proteins (**Figure 2B**). The ω-secalins were almost only detected in the ω-secalin-GPT (≈99% of total ω-secalins). HMW-secalins were detected in all rye GPTs, but with a noticeable enrichment in the appropriate HMW-secalin-GPT (≈96% of total HMW-secalins). The HMW-secalin-GPT contained almost only HMW-secalins. The γ-75k-secalin-GPT contained a very high proportion of γ-75k-secalins (≈95% of total γ-75k-secalins) and lower amounts of HMW-secalins, avenin-like proteins and LMW-GS. In comparison, the γ-40k-secalin-GPT comprised mainly γ-prolamins and γ-75k-secalins with a lower proportion of HMW-secalins. The avenin-like proteins were enriched in the γ-75k-secalin-GPT. The average CV for the tryptic cleavage of the GPTs of rye was 10% and for the chymotryptic cleavage 6%.

#### Barley

The barley GPTs were grouped into the following categories: D-hordeins, B-hordeins, γ3-hordeins, C-hordeins, avenin-like proteins, and HMW-GS from *Triticum aestivum* and a similar tribe (*C*) in the family *Poaceae*. In comparison with the other barley GPTs, the C-hordein-GPT contained the highest amount of C-hordeins (≈96% of total C-hordeins) and a high proportion of D-hordeins. The D-hordeins were also detected in the B-hordein-GPT, but they accounted for the largest share of their appropriate GPT (≈90% of total D-hordeins). B- and γ-hordein-GPTs were mainly composed of B-hordeins, whereas the B-hordein-GPT showed noticeably higher proportions of the B-hordeins (≈77% of total B-hordeins) and also of proteins of the other groups analyzed (**Figure 2C**). The γ-hordein-GPT showed a clear enrichment of the B-hordeins. For the tryptic cleavage of the barley GPTs the average CV was 9% and for the chymotryptic cleavage 10%.

## Discussion

In this study, we provided novel insights into the complexity of gluten from wheat, rye, and barley by identification of the individual proteins and relative quantitation of the most abundant gluten proteins in the GPTs. A preparative strategy (Schalk et al., 2017) was used to isolate the GPTs from wheat, rye and barley flours according to solubility and hydrophobicity. The LC-MS/MS experiments confirmed an enrichment of the expected gluten proteins in their corresponding GPTs in most cases. The application of high-resolution MS allowed a much more detailed and accurate insight into the composition of the isolated GPTs compared to our earlier low-resolution MS analyses (Schalk et al., 2017). The data of the undirected LC-MS/MS experiments showed the qualitative composition of the GPTs, according to the number of peptides identified and revealed a first assumption of the total composition of each GPT. All GPTs contained gluten proteins other than those derived from the known RP-HPLC retention times as well as ATIs, enzymes or uncharacterized proteins. These findings underline the incomplete separation of prolamins and glutelins according to solubility and show that even the separation by preparative RP-HPLC is not clear-cut enough to separate individual GPTs without co-purifying other components, such as ATIs (Junker et al., 2012).

The undirected LC-MS/MS experiments revealed that the group of gluten proteins constituted the highest proportion in the wheat GPTs followed by the second largest group of ATIs, which were present especially in the ω5- and ω1,2-gliadin-GPTs. The MRM data showed that the group of gluten proteins had different compositions of α-, γ-, ω-gliadins, LMW-GS, and HMW-GS, mostly enriched in their appropriate GPTs. However, we found that the LMW-GS were detected in all wheat GPTs. Recently, the presence of LMW-GS in the gliadin fraction has been reported as well (Boukid et al., 2019). Due to their polymeric nature (Shewry, 2019), their similarity to α-gliadins in molecular weight and also to γ-gliadins in RP-HPLC retention times, it may not be possible to achieve a clear-cut separation between those GPTs. Thus, small proportions of LMW-GS were contained in all wheat GPTs.

The ω- and HMW-secalin-GPTs showed high proportions of gluten proteins in the undirected LC-MS/MS analysis. The subsequent MRM analyses revealed that the gluten protein fractions were highly enriched with the expected protein types. As described in previous studies, HMW-secalins were detected with notably high proportions in the other rye GPTs. In case of the ω-secalin-GPT this may be due to the reduction of the disulfide bonds of the HMW-secalins, which then co-eluted in the ω-secalin-GPT (Gellrich et al., 2003). When fractionating rye gluten proteins, we observed that the separation according to solubility is even less complete than in wheat. This led to a higher co-mingling of the individual GPTs even after preparative RP-HPLC. The detection of LMW-GS and avenin-like proteins beside the main group γ-75k-secalins in this GPT may give another hint for the similarity of those GPTs due to the close genetic relationship of rye and wheat (Kasarda et al., 1983). There was no reliable reference sequence available for the γ-40k-secalins (June 2019), but the group named γ-prolamins was only detected in the γ-40k-secalin-GPT. Although the molecular weight (UniProtKB database) of the γ-prolamins detected was somewhat too low compared to the generally known mass range for γ-40k-secalins, the assignment to this GPT would be possible due to amino acid sequence, organism and similarity to other rye proteins. This fact showed the incompleteness of the rye protein entries in the UniProtKB database, because these γ-prolamins were very similar to previously identified ones (Schalk et al., 2017).

The same separation issue as for the rye GPTs appeared for barley GPTs. As stated by Schalk et al. (2017), γ/B-hordeins from the prolamin fraction contained the monomeric γ-hordeins and partly the disulfide-bound B-hordeins. The B/γ-hordeins prepared from glutelin fraction showed the opposite case with the majority of oligomeric or polymeric B-hordeins. Similar results were obtained in this study, except that the γ-hordeins were detected with similar proportions in all barley GPTs. The same applied to the D-hordeins, which were clearly enriched in the D-hordein-GPT, but also identified with noticeably high amounts in the other GPTs. This may also be traced back to the customized separation technique. The identification of hordeins revealed again the challenge with incomplete or unannotated protein entries in the database (Colgrave et al., 2013). Especially the number of entries for barley and rye were low and many proteins were matched as uncharacterized proteins. Reliable protein reference sequences, especially for the *Hordeum* sp. and *Secale* sp. are urgently needed, because the proteomics results are likely to be affected by the drastically different number of protein sequences available.

One limitation of the current study is that the results are based on the analysis of GPTs isolated from one single cultivar of each grain grown in one year. Although the choice of the cultivars was done carefully to select representative samples, genetic and environmental factors and their interaction are known to influence the proteome composition of cereals (Hajas et al., 2018; Juhasz et al., 2018; Malalgoda et al., 2018; Geisslitz et al., 2019). The results obtained here thus only provide one snapshot and are expected to change depending on the flour sample. The overall procedure from milling to collecting sufficient amounts of GPTs after preparative RP-HPLC is rather time-consuming as well as cost- and labor-intensive, so that it is impossible to do this for more than a very limited number of samples. This is why the current study first focused on determining the efficiency of fractionation of the various GPTs, prior to studying the variability arising from different factors.

This study also revealed that trypsin is preferred for the identification experiments for almost all GPTs, except for ω1,2-gliadins and LMW-GS, which were better characterized using the chymotryptic hydrolysate to increase sequence coverage. This may be in part due to the fact that ω1,2-gliadins are more resistant to trypsin and have less K/R (trypsin cleavage sites), so these will be under-represented compared to “other” proteins that have higher K/R and hence more tryptic peptides, such as HMW-GS (Alves et al., 2018). However, for the identification of specific gluten proteins, chymotrypsin yielded more results, because it is shown that the enrichment is more specific and that the trypsin data for some GPTs might be misleading. In general, gluten contains few lysine and arginine residues, but it seems that trypsin was still mostly superior to chymotrypsin due to its cleavage specificity, efficiency and delivery of peptides with favorable chromatographic and MS properties in terms of ionization and fragmentation, as has been reported before (Colgrave et al., 2017b). Most peptides were tryptic, but some were also generated from aspecific cleavage sites. We also observed that the identified proteins and their ranks change depending on the cleavage enzyme used. Due to a number of confounding factors, it is hard to make an assessment which enzyme is more representative of the truth, which is why the results of both approaches were combined in **Figure 2**. Further experiments would be necessary using additional enzymes with different cleavage specificities to investigate this in more detail. The undirected LC-MS/MS analysis of the chymotryptic hydrolysates seemed to be more suitable for the detection of peptides with CD-active epitopes, because significantly more of these peptides were identified than after tryptic hydrolysis. It is known that peptides containing CD-active epitopes are typically resistant to cleavage by trypsin and may therefore be identified in a low amount (Shan et al., 2005). In total, 15 out of 20 different CD-active epitopes were detected. Of the five that were not detected, two (DQ2.5-glia-γ4a, DQ2.5-glia-γ4d) were not present either in historical and modern spring wheat cultivars (Malalgoda et al., 2018).

To conclude, the combination of discovery proteomics and relative quantitation of gluten proteins provided novel insights into the relative amounts of the individual proteins in purified GPTs. These well-defined materials are suitable for a wide range of applications and have already been used as reference materials to quantitate gluten from wheat, rye and barley using targeted LC-MS/MS (Schalk et al., 2018a; Schalk et al., 2018b), as stimulatory agents for epitope mapping (Röckendorf et al., 2017) and for recognition profiling of monoclonal antibodies (Lexhaller et al., 2017). Further potential uses are a variety of functional assays to study mechanisms of immune activation. Our findings raise awareness of the challenges of obtaining “pure” GPTs for analytical purposes and clinical studies on disease mechanisms. Especially when applying gluten or gluten fractions in studies on pathomechanisms of, e.g., CD, NCGS, or WDEIA, it is essential to know which proteins are present in the fractions of interest to establish relationships between structure, functionality and bioactivity.

## Data Availability Statement

The mass spectrometry proteomics data have been deposited to the ProteomeXchange Consortium (http://proteomecentral.proteomexchange.org) with the dataset identifier PXD016065 and are publicly available on Panorama Public (https://panoramaweb.org/nOlizr.url).

## Author Contributions

BL planned and performed the experiments, analyzed the data, designed the figures and wrote the original draft. MC provided access to the LC-MS/MS instruments, contributed to proteomics data analysis and study design. KS was responsible for study conceptualization, contributed to funding acquisition and editing of the manuscript. All authors reviewed and edited the manuscript and approved the final version.

## Funding

This research project (No. 250645717) was supported by the German Research Foundation (Deutsche Forschungsgemeinschaft, DFG, Bonn). BL received additional funding from the Technical University of Munich through the TUM Graduate School Partnership Mobility Grant, the TUM Graduate School Internationalization Grant and a travel grant from the Silesia-Clemens-Hanke Stiftung.

## Conflict of Interest

The authors declare that the research was conducted in the absence of any commercial or financial relationships that could be construed as a potential conflict of interest.
