# Supplementary material for: Characterization and Relative Quantitation of Wheat, Rye, and Barley Gluten Protein Types by Liquid Chromatography–Tandem Mass Spectrometry
Source: Front Plant Sci. 2019 Dec 13;10:1530. doi: 10.3389/fpls.2019.01530 (PMC6923249; doi:10.3389/fpls.2019.01530)
Supplement: Supplementary file 1 [file DataSheet_1.pdf]

Supplementary Material

1 Supplementary Tables

1.1 Supplementary Table S1

Contents of crude protein, albumin/globulin (ALGL), prolamin, and glutenin fractions in the flours.

Flour	Crude protein	ALGL	Prolamins	Glutelins	insoluble residue[a]
			[g/100 g of flour]		
Wheat	9.93 ± 0.14	1.61 ± 0.04	4.94 ± 0.10	2.66 ± 0.11	0.72 ± 0.10
Rye	5.81 ± 0.29	1.77 ± 0.03	2.82 ± 0.08	0.46 ± 0.07	0.76 ± 0.12
Barley	6.72 ± 0.04	1.58 ± 0.03	1.99 ± 0.12	1.66 ± 0.10	1.49 ± 0.07

Values are given as means ± standard deviation (n = 3), [a] Difference between the crude protein content and the sum of ALGL, prolamin, and glutelin contents quantified by RP-HPLC

1.2 Supplementary Table S2

Crude protein contents of the gluten protein types (GPTs) isolated from wheat, rye and barley flours and the proportions of each GPT within total gluten.

Flour	GPT	Crude protein[a]	Proportions of gluten[b]
		[%]	[%]
Wheat	α-gliadins	79.2 ± 1.1	33.5
	γ-gliadins	64.2 ± 0.8	21.6
	ω5-gliadins	63.6 ± 0.6	4.8
	ω1,2-gliadins	89.4 ± 0.6	5.9
	HMW-GS	71.0 ± 1.9	9.3
	LMW-GS	79.6 ± 0.9	24.9
Rye	ω-secalins	99.5 ± 0.4	28.4
	HMW-secalins	96.3 ± 0.5	5.8
	γ-75k-secalins	75.6 ± 1.8	39.9
	γ-40k-secalins	85.0 ± 1.0	25.9
Barley	C-hordeins	99.6 ± 0.4	19.0
	γ/B-hordeins	99.4 ± 0.5	35.5
	B/γ-hordeins	97.0 ± 0.1	39.4
	D-hordeins	50.0 ± 1.5	6.1

[a] Values are given as means ± standard deviation (n = 3), [b] Determined by RP-HPLC

1.3 Supplementary Table S3

Identified proteins (1% global FDR) in each wheat, rye and barley gluten protein type after tryptic hydrolysis with their UniProtKB accession number, species, name, rank, score, coverage and number of identified peptides. The rank of a specific protein is given relative to all other detected proteins.

Uniprot accession	Species	Name	Rank[a]	Score[b]	Cove-rage	Pep-tides
**Wheat**						
**α-gliadins**						
R9XUM8	Triticum aestivum	Alpha-gliadin	1	30.03	44.3	96
B2Y2Q4	Triticum aestivum	Low molecular weight glutenin subunit	2	17.12	36.9	55
P17314	Triticum aestivum	Alpha amylase inhibitor CM3	3	13.52	54.2	18
A0A060MZP1	Triticum aestivum	High molecular weight glutenin subunit (Fragment)	5	11.23	13.7	19
W6AX70	Triticum aestivum	High molecular weight glutenin subunit	6	10.25	18.8	18
Q2A784	Triticum aestivum	Avenin-like a1	7	10.15	35.1	16
A0A1D6DC72	Triticum aestivum	Uncharacterized protein	8	9.75	40.9	16
BLAST search: D2KFG9 Gliadin/avenin-like seed protein (identity: 89.7%)						
A5JSA9	Triticum aestivum	Alpha-gliadin	9	9.08	56.1	91
P01084	Triticum dicoccoides	Alpha-amylase inhibitor 0.53	10	8.78	54.0	6
A0A0K0KDM6	Triticum aestivum	High molecular weight glutenin subunit 1Dy3	11	8.17	11.9	13
X2KYP9	Triticum aestivum	Monomeric alpha-amylase inhibitor	12	7.98	35.8	5
I7KM78	Triticum aestivum	Gamma-gliadin	13	7.19	26.3	11
C5IFV2	Triticum aestivum	Low molecular weight protein (Fragment)	14	7.12	29.6	20
D0ES80	Triticum aestivum	Gamma-gliadin (Fragment)	15	5.85	26.4	9
C8CAI4	Triticum aestivum	Dimeric alpha-amylase inhibitor	16	5.57	54.6	7

A0A0E3UQX9	Triticum spelta	Alpha-gliadin (Fragment)	17	5.53	60.1	64
W8PU74	Brachypodium distachyon	Farinin protein	18	5.27	18.6	4
R9XV91	Triticum aestivum	LMW-GS	19	4.92	26.1	29
B9VRI3	Triticum macha	Alpha-amylase inhibitor CM16 subunit (Fragment)	20	4.84	21.8	3
A7X9X6	Triticum turgidum	Low molecular weight glutenin (Fragment)	21	4.69	34.5	22
P10386	Triticum aestivum	Glutenin, low molecular weight subunit 1D1	22	4.45	31.6	44
A0A1K0J4V2	Triticum aestivum	Alpha-gliadin	23	4.41	33.3	38
Q2A783	Triticum aestivum	Avenin-like b1	24	4.17	28.4	11
P33432	Triticum aestivum	Puroindoline-A	25	3.46	20.3	4
B6DQB2	Triticum aestivum	Gamma gliadin (Fragment)	27	3.13	12.5	9
D2KFG9	Triticum aestivum	Gliadin/avenin-like seed protein	28	2.85	28.4	16
N1QTW5	Aegilops tauschii	Trypsin inhibitor CMc	29	2.75	46.8	9
Q43723	Triticum aestivum	Trypsin/alpha-amylase inhibitor CMX1/CMX3	30	2.64	7.4	1
D2KFH0	Triticum aestivum	Gliadin/avenin-like seed protein	31	2.56	19.1	3
K7WV42	Triticum aestivum	Alpha-gliadin	32	2.45	62.1	83
I0IT55	Triticum aestivum	Alpha/beta-gliadin	34	2.27	49.1	77
Q05806	Triticum aestivum	Type-5 thionin	35	2.25	7.6	1
M8BV45	Aegilops tauschii	Alpha-amylase/trypsin inhibitor CM3	36	2.20	48.9	14
Q1ZZT4	Triticum aestivum	Low-molecular-weight glutenin subunit	37	2.06	27.8	22
V9P779	Triticum aestivum	LMW-m glutenin subunit 38	38	2.01	34.2	45

A0A1D5X2J6	Triticum aestivum	Uncharacterized protein	39	2.01	9.6	2
Q5UHH6	Triticum aestivum	0.19 dimeric alpha-amylase inhibitor (Fragment)	40	2.00	45.0	6
A0A0K2QJU7	Triticum aestivum	Alpha/beta-gliadin	41	2.00	63.2	95
A0A1Z5R8D6	Sorghum bicolor	Uncharacterized protein	42	2.00	2.9	1
A0A0A9NGF0	Arundo donax	Uncharacterized protein	43	2.00	1.7	7
**γ-gliadins**						
Q41553	Triticum aestivum	HMW glutenin subunit Ax2	1	27.66	25.4	31
I3XHQ1	Triticum aestivum	Low molecular weight glutenin subunit LMW-9	3	24.08	32.9	38
W6AX70	Triticum aestivum	High molecular weight glutenin subunit	4	20.79	28.7	25
D0ES80	Triticum aestivum	Gamma-gliadin	5	20.59	32.2	51
H8Y0P9	Australopy-rum retrofractum	Gamma prolamin (Fragment)	7	13.83	34.4	31
A0A0E3Z6U5	Triticum spelta	Alpha-gliadin (Fragment)	8	13.51	24.3	13
W0C8N8	Triticum aestivum	High-molecular-weight glutenin subunit 1Bx1	9	13.02	11.3	15
P10387	Triticum aestivum	Glutenin, high molecular weight subunit DY10	10	12.30	23.2	25
P17314	Triticum aestivum	Alpha-amylase/trypsin inhibitor CM3	13	10.00	38.7	5
X2JAE7	Triticum aestivum	Low-molecular-weight glutenin subunit Glu-A3 (Fragment)	14	9.57	20.1	23
W8PU74	Brachypodium distachyon	Farinin protein	16	9.16	27.5	6
Q8H0J4	Triticum aestivum	Low molecular weight glutenin subunit (Fragment)	18	8.13	26.3	16
P33432	Triticum aestivum	Puroindoline-A	19	7.74	26.4	5
Q4U198	Triticum aestivum	Dimeric alpha-amylase inhibitor	20	7.57	41.8	6
C4P598	Triticum	Monomeric alpha-amylase	21	7.45	29.1	4

	dicoccoides	inhibitor				
V9P785	Triticum aestivum	LMW-m glutenin subunit 50 (Fragment)	22	6.84	25.6	16
J7HUT0	Aegilops umbellulata	Gamma-gliadin (Fragment)	23	5.40	16.5	10
A0A286QSW6	Triticum aestivum	Avenin-like protein A4	24	4.97	16.6	4
I7KM78	Triticum aestivum	Gamma gliadin	25	4.82	14.4	5
Q6RS99	Triticum turgidum	Globulin 1	26	4.52	17.0	3
P01084	Triticum aestivum	Alpha-amylase inhibitor 0.53	28	3.96	47.6	5
Q84QI5	Triticum aestivum	GSP-1 Grain Softness Protein	29	3.94	22.0	4
B9VRI3	Triticum macha	Alpha-amylase inhibitor CM16 subunit (Fragment)	30	3.76	21.8	3
A0A1D6DC72	Triticum aestivum	Uncharacterized protein	32	3.48	14.8	5
BLAST search: D2KFG9 Gliadin/avenin-like seed protein (identity: 89.7%)						
A0A1D5XGF4	Triticum aestivum	Beta-amylase	33	3.46	5.4	2
P10388	Triticum aestivum	Glutenin, high molecular weight subunit DX5	34	3.23	6.1	6
M8BAK8	Aegilops tauschii	Uncharacterized protein	35	3.15	13.8	2
BLAST search: Q43723 Trypsin/alpha-amylase inhibitor CMX1/CMX3 (identity: 92.6%)						
V9P779	Triticum aestivum	LMW-m glutenin subunit 38	36	2.72	31.0	40
P04727	Triticum aestivum	Alpha/beta-gliadin clone PW8142	37	2.71	14.4	10
A8IE96	Thinopyrum ponticum x Triticum aestivum	Low molecular weight glutenin subunit	38	2.61	22.8	15
P08453	Triticum aestivum	Gamma-gliadin	39	2.27	17.1	26
Q18NR2	Triticum aestivum	LMW glutenin subunit	40	2.20	37.0	40
A0A287NA39	Hordeum	Uncharacterized protein	41	2.17	2.5	1

	vulgare subsp. vulgare					
BLAST search: P06293 Serpin-Z4 (identity: 99.0%)						
W5EFT2	Triticum aestivum	Uncharacterized protein	42	2.13	4.4	1
A0A1P8DTB3	Triticum spelta	Alpha-gliadin storage protein (Fragment)	43	2.08	18.5	10
W5AKY9	Triticum aestivum	Uncharacterized protein	44	2.03	1.9	1
BLAST search: M7ZK46 12S seed storage globulin 1 (identity: 94.5%)						
C3VWG2	Triticum dicoccoides	Dimeric alpha-amylase inhibitor	45	2.01	30.5	5
A0A1D5X2J6	Triticum aestivum	Uncharacterized protein	46	2.01	9.6	1
BLAST search: P0CZ11 Avenin-like a7 (identity: 56.3%)						
A0A1K0IT13	Triticum aestivum	Alpha-gliadin	47	2.00	20.8	11
P01084	Eremopyrum bonaepartis	Alpha-amylase inhibitor 0.53	48	2.00	47.6	5
M8B2U4	Aegilops tauschii	Uncharacterized protein	49	2.00	14.2	2
BLAST search: Q43723 Trypsin/alpha-amylase inhibitor CMX1/CMX3 (identity: 91.7%)						
A7XUM7	Aegilops triuncialis	Avenin-like protein	50	2.00	9.9	2
Q43769	Hordeum vulgare	Oleosin	51	2.00	4.9	1
M8ALF6	Aegilops tauschii	Subtilisin-like protease	52	2.00	1.4	1
K4A391	Setaria italica	Uncharacterized protein	53	2.00	2.2	1
I4EP89	Avena strigosa	Avenin	54	2.00	5.0	1
I3NV45	Triticum aestivum	Centromeric histone 3	55	2.00	6.7	2
A0A1D6AS82	Triticum aestivum	Uncharacterized protein	57	2.00	6.5	1
BLAST search: P16969 Trypsin/alpha-amylase inhibitor (identity: 84.8%)						
A0A0E0L8P7	Oryza punctata	Uncharacterized protein	58	2.00	1.2	1
A0A0E0CG60	Oryza	Uncharacterized protein	59	2.00	4.3	1

	meridionalis					
**ω5-gliadins**						
Q41553	Triticum aestivum	HMW glutenin subunit Ax2	1	52.89	29.0	56
V9TRL3	Triticum aestivum	High molecular weight glutenin subunit 1Dy protein	4	21.91	22.0	24
P10388	Triticum aestivum	Glutenin, high molecular weight subunit DX5	6	16.73	8.3	17
W6AX70	Triticum aestivum	High molecular weight glutenin subunit	7	16.04	21.4	16
S5SCQ8	Secale cereale x Triticum aestivum	High-molecular-weight glutenin subunit	10	10.78	8.9	13
C8CAI4	Triticum aestivum	Dimeric alpha-amylase inhibitor	11	9.42	46.1	9
A0A0B5J8A9	Triticum aestivum	Omega-gliadin	12	8.00	16.5	8
P16159	Triticum aestivum	Alpha-amylase/trypsin inhibitor CM16	13	8.00	33.6	5
Q9XGF0	Triticum turgidum subsp. durum	Low molecular weight glutenin subunit (Fragment)	14	6.47	10.6	4
P01085	Triticum aestivum	Alpha-amylase inhibitor 0.19	15	6.00	52.4	8
F2E8X4	Hordeum vulgare subsp. vulgare	Oleosin	17	3.96	14.4	2
W5D5L4	Triticum aestivum	Fructose-bisphosphate aldolase	18	3.15	6.7	2
Q9SC06	Triticum monococcum	Grain softness related protein (Fragment)	20	2.01	10.4	1
Q43770	Hordeum vulgare	Oleosin	21	2.01	7.6	1
Q5UHH6	Triticum aestivum	0.19 dimeric alpha-amylase inhibitor (Fragment)	22	2.00	47.6	6
Q8LPA7	Triticum aestivum	Cold shock protein-1	23	2.00	7.9	1
A0A1D5WQ92	Triticum aestivum	Uncharacterized protein	24	2.00	1.7	2
W5GUD5	Triticum	Uncharacterized protein	25	2.00	4.0	1

	aestivum					
W5G4V0	Triticum aestivum	Uncharacterized protein	26	2.00	13.9	1
Q7XYC3	Triticum aestivum	19 kDa globulin (Fragment)	27	2.00	6.6	1
Q8GZB0	Triticum aestivum	Non-specific lipid-transfer protein	28	2.00	9.5	1
Q41546	Triticum aestivum	Alpha/beta-gliadin storage protein	30	2.00	2.9	1
K4ABC8	Setaria italica	Uncharacterized protein	31	2.00	3.7	1
K4A308	Setaria italica	Uncharacterized protein	32	2.00	2.9	1
F2CR79	Hordeum vulgare subsp. vulgare	Predicted protein	33	2.00	3.7	1
**ω1,2-gliadins**						
G9I1R7	Aegilops comosa	Alpha-gliadin Gli-M2	5	24.59	36.2	29
C8CAI4	Triticum aestivum	Dimeric alpha-amylase inhibitor	7	24.10	73.0	28
B9VRI3	Triticum macha	Alpha-amylase inhibitor CM16 subunit (Fragment)	8	20.01	62.7	28
P16851	Triticum aestivum	Alpha-amylase/trypsin inhibitor CM2	9	19.55	60.7	28
A0A060MZP1	Triticum aestivum	High molecular weight glutenin subunit (Fragment)	10	19.20	25.8	36
X2KYP9	Triticum aestivum	Monomeric alpha-amylase inhibitor	11	18.01	57.0	11
Q8W3V1	Triticum aestivum	Low-molecular-weight glutenin subunit group 11 type VI	12	17.69	37.3	35
P06470	Triticum aestivum	B1-hordein	13	17.41	26.3	11
W6AX70	Triticum aestivum	High molecular weight glutenin subunit	14	16.26	31.9	36
B8XU28	Triticum monococcum	Alfa gliadin	15	14.85	22.4	13
S5SCQ8	Secale cereale x Triticum aestivum	High-molecular-weight glutenin subunit	17	12.41	18.8	24
V5RL87	Triticum aestivum	Puroindoline	18	12.22	55.4	25

V9TRL3	Triticum aestivum	High molecular weight glutenin subunit 1Dy protein	19	11.80	26.8	22
M8BZV7	Aegilops tauschii	Uncharacterized protein	20	11.34	38.7	21
BLAST search: P0CZ09 Avenin-like a5 (identity: 98.8%)						
P17314	Triticum aestivum	Alpha amylase/trypsin inhibitor CM3	22	8.31	36.3	4
A0A173DQZ7	Triticum aestivum	Type-b avenin-like protein	23	8.19	27.5	7
P16850	Triticum aestivum	Alpha-amylase/trypsin inhibitor CM1	24	8.02	47.8	25
Q5MD68	Triticum aestivum	0.19 dimeric alpha-amylase inhibitor (Fragment)	25	8.01	66.9	20
Q41540	Triticum aestivum	CM 17 protein	26	8.00	56.6	24
P10388	Triticum aestivum	Glutenin, high molecular weight subunit DX5	27	6.24	13.6	24
Q84QI5	Triticum aestivum	GSP-1 Grain Softness Protein	28	6.15	15.2	3
K7XEB0	Triticum aestivum	Alpha-gliadin	29	6.09	33.8	29
Q84LE9	Hordeum vulgare	D-Hordein	30	6.03	9.4	16
C3VWQ4	Triticum dicoccoides	Dimeric alpha-amylase inhibitor	31	6.00	44.7	14
A0A1D6DC72	Triticum aestivum	Uncharacterized protein	33	4.25	14.8	4
BLAST search: D2KFG9 Gliadin/avenin-like seed protein (identity: 89.7%)						
I6TEV5	Hordeum vulgare	B3 hordein	34	4.10	23.5	8
A0A1P8DSZ1	Triticum spelta	Alpha-gliadin storage protein (Fragment)	35	4.07	25.7	20
W8PU74	Brachypodium distachyon	Farinin protein	36	4.01	14.3	4
X2L2H1	Triticum aestivum	Alpha-gliadin protein	37	4.00	41.5	31
B7S4N5	Thinopyrum intermedium	HMW gluten subunit	39	4.00	17.6	16
P33432	Triticum	Puroindoline-A	40	4.00	55.4	8

	aestivum					
A0A1D5X2J6	Triticum aestivum	Uncharacterized protein	41	4.00	14.7	3
BLAST search: B8YG97 Avenin-like b11 (identity: 57.9%)						
Q8GZB0	Triticum aestivum	Non-specific lipid-transfer protein	42	2.92	27.6	3
A0A287NA39	Hordeum vulgare subsp. vulgare	Uncharacterized protein	43	2.83	7.6	3
BLAST search: P06293 Serpin-Z4 (identity: 99.0%)						
M8BDX3	Aegilops tauschii	Uncharacterized protein	44	2.54	5.7	2
Q7XYC3	Triticum aestivum	19 kDa globulin (Fragment)	45	2.46	4.2	1
P52589	Triticum aestivum	Protein disulfide-isomerase	46	2.30	1.9	1
Q43770	Hordeum vulgare	Oleosin	47	2.20	15.8	2
V9QFJ0	Cochliobolus miyabeanus	Elongation factor-1a (Fragment)	49	2.07	3.5	1
Q5UHH8	Triticum aestivum	0.19 dimeric alpha-amylase inhibitor (Fragment)	50	2.06	75.0	26
A0A1D5TKE5	Triticum aestivum	Uncharacterized protein	51	2.05	6.4	1
BLAST search: P29546 Elongationsfactor 1-beta (identity: 95.4%)						
W5B1E5	Triticum aestivum	Superoxide dismutase [Cu-Zn]	52	2.01	7.9	1
I0IT62	Triticum aestivum	Alpha/beta-gliadin	53	2.00	22.7	26
I6PWK7	Triticum aestivum	Dimeric alpha-amylase inhibitor (Fragment)	54	2.00	66.0	17
M7ZIU1	Triticum urartu	Glutenin, low molecular weight subunit	55	2.00	20.5	33
Q41546	Triticum aestivum	Alpha/beta-gliadin storage protein	56	2.00	21.7	19
A0A287EFH8	Hordeum vulgare subsp. vulgare	Uncharacterized protein	57	2.00	20.3	9
BLAST search: P06470 B1-hordein (identity: 93.9%)						
C3VW87	Triticum	Dimeric alpha-amylase	58	2.00	51.8	17

	dicoccoides	inhibitor				
C3VWV4	Eremopyrum bonaepartis	Dimeric alpha-amylase inhibitor	59	2.00	38.3	10
I7KM78	Triticum aestivum	Gamma-gliadin	60	2.00	8.6	2
Q53WS1	Triticum aestivum	Alpha 1 purothionin	61	2.00	6.6	1
J7HYA4	Aegilops umbellulata	Gamma-gliadin (Fragment)	62	2.00	4.4	1
D2KFH0	Triticum aestivum	Gliadin/avenin-like seed protein	63	2.00	3.8	1
W5I6P3	Triticum aestivum	Uncharacterized protein	64	2.00	2.0	1
BLAST search: M8AZH0 Enolase 1 (identity: 99.3%)						
W5HZ47	Triticum aestivum	Fructose-bisphosphate aldolase	65	2.00	3.9	1
A0A1D5ZY48	Triticum aestivum	Uncharacterized protein	66	2.00	3.0	1
J3LDJ0	Oryza brachyantha	Uncharacterized protein	67	2.00	2.8	1
I1HBQ3	Brachypodium distachyon	Uncharacterized protein	68	2.00	2.3	1
I1GWR7	Brachypodium distachyon	Uncharacterized protein	69	2.00	2.7	1
F2DRY3	Hordeum vulgare subsp. vulgare	Predicted protein (Fragment)	70	2.00	4.7	1
F2DHM1	Hordeum vulgare subsp. vulgare	Predicted protein	71	2.00	1.6	1
A2YY55	Oryza sativa subsp. indica	Uncharacterized protein	72	2.00	1.3	1
A0A287WAT6	Hordeum vulgare subsp. vulgare	Uncharacterized protein	73	2.00	3.2	1
**HMW-GS**						
W6AX70	Triticum aestivum	High molecular weight glutenin subunit	1	71.28	46.8	113
A0A060MZP1	Triticum aestivum	High molecular weight glutenin subunit (Fragment)	2	54.59	36.9	123

P10388	Triticum aestivum	Glutenin, high molecular weight subunit DX5	3	41.05	35.6	97
Q45R38	Triticum aestivum	HMW glutenin x-type subunit Bx7	4	39.96	37.2	64
Q0Q5D3	Triticum aestivum	Y-type HMW glutenin	5	35.20	54.6	104
M8CU50	Aegilops tauschii	Uncharacterized protein	7	22.88	61.8	51
BLAST search: A5A4L4 Avenin-like b5 (identity: 72.2%)						
Q0Q5D4	Triticum aestivum	Globulin 1	8	18.53	50.4	28
A0A165R8I1	Triticum aestivum	S-type low molecular weight glutenin subunit	9	14.25	28.6	22
R4JDK8	Triticum aestivum	Low-molecular-weight glutenin subunit	10	12.01	16.6	10
A0A1D6A2L5	Triticum aestivum	Uncharacterized protein	11	11.97	54.6	10
BLAST search: Q99070 Glycine-rich RNA binding protein 2 (identity: 70.6%)						
M8BDX3	Aegilops tauschii	Uncharacterized protein	12	9.64	17.5	5
X2KYP9	Triticum aestivum	Monomeric alpha-amylase inhibitor	13	9.59	34.4	5
Q03033	Triticum aestivum	Elongation factor 1-alpha	14	8.33	11.6	5
R9XV30	Triticum aestivum	Alpha-gliadin	15	8.30	13.3	9
A0A1D5Y5R8	Triticum aestivum	Uncharacterized protein	16	8.00	43.4	5
BLAST search: M4VSR0 Low temperature-responsive RNA-binding protein (identity: 97.0%)						
Q10464	Triticum aestivum	Puroindoline-B	17	8.00	30.4	4
W5B1E5	Triticum aestivum	Superoxide dismutase [Cu-Zn]	18	8.00	28.3	4
Q0PG36	Triticum aestivum	Glucose-1-phosphate adenylyltransferase	20	6.30	5.6	4
P16850	Triticum aestivum	Alpha-amylase/trypsin inhibitor CM1	21	6.26	33.8	7
W5DPI4	Triticum aestivum	Uncharacterized protein	22	6.20	5.1	3
A0A1D5S923	Triticum	Globulin 1	23	6.11	28.9	10

	aestivum					
R9XV91	Triticum aestivum	LMW-GS	24	6.09	20.1	8
Q93XQ8	Triticum aestivum	Protein disulfide isomerase (Fragment)	25	6.07	5.7	3
A0A173DQZ7	Triticum aestivum	Type-b avenin-like protein	27	6.02	13.4	3
Q9FVJ5	Triticum aestivum	GSP-A1	28	6.01	20.7	4
A0A1D5ZLT8	Triticum aestivum	Uncharacterized protein	29	6.01	25.4	3
Q8H0B8	Triticum aestivum	Cold regulated protein	30	6.00	21.8	3
W5FAY5	Triticum aestivum	Actin	31	6.00	8.5	3
W5IA32	Triticum aestivum	Formate dehydrogenase, mitochondrial	32	5.96	12.7	4
A0A1G4P1V3	Triticum aestivum	HMW glutenin x-type subunit 1Ax21	33	5.35	25.3	63
D2KFH0	Triticum aestivum	Gliadin/avenin-like seed protein	34	4.56	16.4	2
Q41540	Triticum aestivum	CM 17 protein	35	4.39	25.2	4
M8BZV7	Aegilops tauschii	Uncharacterized protein	36	4.32	20.8	6
BLAST search: P0CZ09 Avenin-like b5 (identity: 98.8%)						
M0V3U0	Hordeum vulgare subsp. vulgare	Non-specific lipid-transfer protein	37	4.08	32.5	3
Q41518	Triticum aestivum	Single-stranded nucleic acid binding protein	38	4.07	66.5	11
H6UQP6	Elymus libanoticus	High molecular weight subunit of glutenin	39	4.07	39.6	25
G8CNJ3	Psathyrostachys juncea	Puroindoline a	41	4.03	14.8	2
T1NLC6	Triticum urartu	Uncharacterized protein	42	4.00	46.1	2
BLAST search: A0A3B5ZWW6 HSP 70 (identity: 98.1%)						
I3NM41	Triticum aestivum	Oleosin	43	4.00	16.2	2

P93602	Triticum aestivum	PUP88 protein; member of trypsin/a-amylase inhibitors family from cereals	45	3.92	13.1	2
Q94F69	Triticum aestivum	Peptidyl-prolyl cis-trans isomerase (Fragment)	46	3.75	22.1	2
W5FPN2	Triticum aestivum	Uncharacterized protein	47	3.74	6.1	2
M7ZK46	Triticum urartu	12S seed storage globulin 1	48	3.31	4.6	2
B7S4N5	Thinopyrum intermedium	HMW gluten subunit	50	3.19	40.7	75
A0A1D5UXT7	Triticum aestivum	Cysteine proteinase inhibitor	51	3.10	17.9	2
Q0KIW2	Triticum aestivum	Glycine-rich RNA-binding protein	52	3.07	42.3	5
M4VSR0	Triticum aestivum	Low temperature-responsive RNA-binding protein	53	2.98	43.2	5
W5C8N6	Triticum aestivum	40S ribosomal protein S6	54	2.86	9.2	2
W5DK32	Triticum aestivum	Uncharacterized protein	55	2.85	17.5	2
BLAST search: P01085 Alpha-amylase inhibitor 0.19 (identity: 100%)						
D6RVY4	Triticum aestivum	Low molecular glutenin subunit (Fragment)	57	2.72	33.1	25
Q7XYE2	Triticum aestivum	Catalase isozyme (Fragment)	58	2.52	9.7	3
Q9FUU7	Triticum aestivum	Starch branching enzyme 2	60	2.34	1.0	1
Q0Q5E3	Triticum aestivum	Globulin 1	61	2.21	44.5	21
Q3S4I1	Triticum aestivum	Eukaryotic translation initiation factor 5A	62	2.20	6.2	1
Q53WS1	Triticum aestivum	Alpha 1 purothionin	64	2.14	15.4	2
Q43723	Triticum aestivum	Trypsin/alpha-amylase inhibitor CMX1/CMX3	66	2.09	6.8	1
Q9ZRB0	Triticum aestivum	Tubulin beta-3 chain	67	2.08	5.2	2
W5D591	Triticum aestivum	Small ubiquitin-related modifier	68	2.06	9.9	1
A0A1D5US77	Triticum	Uncharacterized protein	69	2.04	3.5	1

	aestivum					
P17314	Triticum aestivum	Alpha amylase/trypsin inhibitor CM3	70	2.03	8.9	1
W5BVA4	Triticum aestivum	Uncharacterized protein	71	2.02	8.7	3
W5E8X2	Triticum aestivum	Uncharacterized protein	72	2.02	18.5	1
A0A1D5UB33	Triticum aestivum	Uncharacterized protein	73	2.02	6.7	1
BLAST search: P16159 Alpha-amylase/trypsin inhibitor CM16 (identity: 42.3%)						
E7DVE3	Aegilops kotschyi	High molecular weight glutenin subunit Ux2.3	74	2.01	15.6	42
W5FTF6	Triticum aestivum	Uncharacterized protein	75	2.01	10.1	1
Q0GLF2	Aegilops tauschii	High molecular weight glutenin 1Dx1.6t	76	2.00	23.4	60
Q0Q5D9	Triticum aestivum	Globulin 1	77	2.00	36.0	17
A0A060N0C7	Triticum aestivum	Low molecular weight glutenin subunit (Fragment)	78	2.00	22.3	14
V9XYU2	Pseudoroegneria strigosa	High molecular weight glutenin subunit	79	2.00	42.9	31
Q41546	Triticum aestivum	Alpha/beta-gliadin storage protein	80	2.00	13.1	7
P16851	Triticum aestivum	Alpha-amylase/trypsin inhibitor CM2	81	2.00	32.4	6
B2Y2S3	Triticum aestivum	Low molecular weight glutenin subunit	82	2.00	13.4	25
A8QRK0	Triticum dicoccoides	Grain softness protein	83	2.00	15.3	3
P16159	Triticum aestivum	Alpha-amylase/trypsin inhibitor CM16	84	2.00	17.5	3
Q9T0P1	Triticum aestivum	Alpha purothionin	85	2.00	15.3	2
W5H208	Triticum aestivum	40S ribosomal protein S24	86	2.00	8.0	1
T1NAD0	Triticum urartu	Malate dehydrogenase	87	2.00	2.5	1
W5I301	Triticum aestivum	Uncharacterized protein	89	2.00	9.2	1

BLAST search: M7ZWX9 60S acidic ribosomal protein P3 (identity: 98.3%)						
W5HZ68	Triticum aestivum	40S ribosomal protein S27	90	2.00	9.3	1
W5FWF5	Triticum aestivum	Uncharacterized protein	91	2.00	7.1	1
BLAST search: M7Y6X6 40S ribosomal protein S19 (identity: 100%)						
W5FNH7	Triticum aestivum	Uncharacterized protein	92	2.00	2.6	1
W5EFT2	Triticum aestivum	Uncharacterized protein	93	2.00	4.4	1
W5E8D7	Triticum aestivum	1,2-dihydroxy-3-keto-5-methylthiopentene dioxygenase	94	2.00	5.4	1
W5DYD1	Triticum aestivum	Uncharacterized protein	95	2.00	2.7	1
W5DIU6	Triticum aestivum	Uncharacterized protein	96	2.00	4.7	1
BLAST search: P46226 triosephosphate isomerase, cytosolic (identity: 96.8%)						
W5CCL7	Triticum aestivum	DNA-(apurinic or apyrimidinic site) lyase	97	2.00	1.5	1
W5ASI4	Triticum aestivum	Uncharacterized protein	98	2.00	1.6	1
W5AKH4	Triticum aestivum	Uncharacterized protein	99	2.00	7.4	1
BLAST search: E6Y3A2 Putative translation initiation factor (identity: 89.6%)						
W5A520	Triticum aestivum	Uncharacterized protein	100	2.00	7.8	1
BLAST search: M7ZRL8 60S ribosomal protein L18-2 (identity: 98.9%)						
A0A1D5UKZ4	Triticum aestivum	40S ribosomal protein S8	101	2.00	6.2	1
A4K4Y8	Triticum aestivum	Tubulin alpha chain	102	2.00	3.3	1
W5FEU1	Triticum aestivum	Uncharacterized protein	103	2.00	5.9	1
A0A1E5W824	Dichanthelium oligosanthes	1-aminocyclopropane-1-carboxylate oxidase-like protein 3 (Fragment)	104	2.00	3.1	1
A0A0A9NGF0	Arundo donax	Uncharacterized protein	105	2.00	1.7	4
A0A077RW06	Triticum aestivum	Uncharacterized protein	106	2.00	4.8	1

**LMW-GS**						
M7ZK46	Triticum urartu	12S seed storage globulin 1	1	26.70	32.6	32
A0A060MZP1	Triticum aestivum	High molecular weight glutenin subunit (Fragment)	2	18.51	22.9	31
D6RVY4	Triticum aestivum	Low molecular glutenin subunit (Fragment)	4	16.56	38.1	73
W6AX70	Triticum aestivum	High molecular weight glutenin subunit	5	15.23	28.9	32
K7X1G8	Aegilops tauschii	Alpha-gliadin	6	12.74	27.5	20
Q93XQ7	Triticum aestivum	Protein disulfide-isomerase	7	10.81	15.5	7
X2KYP9	Triticum aestivum	Monomeric alpha-amylase inhibitor	8	10.26	45.7	8
A0A0K0KDM6	Triticum aestivum	High molecular weight glutenin subunit 1Dy3	10	9.58	16.0	24
Q93W25	Triticum aestivum	Peptidyl-prolyl cis-trans isomerase	11	9.41	36.8	8
A0A1D5YFA7	Triticum aestivum	Beta-amylase	12	8.71	17.6	8
W8PU74	Brachypodium distachyon	Farinin protein	13	8.62	26.8	10
C3VW69	Triticum dicoccoides	Dimeric alpha-amylase inhibitor	14	8.45	53.2	6
R4JB50	Triticum aestivum	Low-molecular-weight glutenin subunit (Fragment)	15	7.86	21.0	39
M8BV45	Aegilops tauschii	Alpha-amylase/trypsin inhibitor CM3	16	7.69	33.9	6
Q8LKV8	Aegilops tauschii	Seed globulin	17	7.03	27.0	6
I3XHQ1	Triticum aestivum	Low molecular weight glutenin subunit LMW-9	18	6.93	32.9	72
P08453	Triticum aestivum	Gamma-gliadin	19	6.82	19.6	9
A0A173DQZ7	Triticum aestivum	Type-b avenin-like protein	20	6.58	28.2	15
Q1ZZT4	Triticum aestivum	Low-molecular-weight glutenin subunit	22	6.17	32.0	34
A5HMG1	Triticum	HMW glutenin subunit	23	5.75	8.7	7

	aestivum	1Bx13				
W5FZ62	Triticum aestivum	Uncharacterized protein	24	5.00	5.0	2
BLAST search: P93693 Serpin-Z1B (identity: 99.5%)						
T1NAD0	Triticum urartu	Malate dehydrogenase	25	4.96	9.1	4
R4JDK8	Triticum aestivum	Low-molecular-weight glutenin subunit	27	4.71	27.8	37
Q41540	Triticum aestivum	CM 17 protein	28	4.53	18.9	3
M8BDK9	Aegilops tauschii	Alanine aminotransferase 2	29	4.15	5.5	3
R9UNY9	Triticum aestivum	Xylanase inhibitor protein	30	4.12	6.3	2
W5CWR9	Triticum aestivum	Uncharacterized protein	31	4.08	7.6	2
B6DQB2	Triticum aestivum	Gamma-gliadin (Fragment)	32	4.05	11.1	5
Q10464	Triticum aestivum	Puroindoline-B	33	4.05	23.7	3
W5EFT2	Triticum aestivum	Uncharacterized protein	34	4.04	12.2	3
V5RL87	Triticum aestivum	Puroindoline	35	4.01	26.4	5
A0A1D5XGF4	Triticum aestivum	Beta-amylase	36	4.00	15.3	6
A0A1D6SDX2	Triticum aestivum	Uncharacterized protein	37	4.00	1.8	2
Q41585	Triticum aestivum	Type V Thionin	38	3.93	21.5	3
C8CAI4	Triticum aestivum	Dimeric alpha-amylase inhibitor	39	3.85	40.4	6
Q07810	Triticum aestivum	rRNA N-glycosidase	41	3.73	6.5	2
A0A077RXH0	Triticum aestivum	Uncharacterized protein	42	3.39	11.5	2
B2BZC7	Triticum aestivum	LMW-m glutenin subunit 0154A5-M	43	3.33	31.9	34
I0IT55	Triticum aestivum	Alpha/beta-gliadin	47	2.86	19.2	13

K7X1I9	Triticum aestivum	Alpha-gliadin	48	2.82	27.7	16
W5B5R3	Triticum aestivum	Sucrose synthase	49	2.81	1.3	1
Q8LRM8	Triticum aestivum	Translationally-controlled tumor protein homolog	50	2.49	5.4	1
Q6W8Q2	Triticum aestivum	1-Cys peroxiredoxin PER1	51	2.47	5.0	1
A0A1D5RYA9	Triticum aestivum	Uncharacterized protein	52	2.45	8.5	1
BLAST search: Q8H0B8 cold regulated protein (identity: 99.4%)						
Q03033	Triticum aestivum	Elongation factor 1-alpha	53	2.36	1.8	1
A0A1D5TK48	Triticum aestivum	Uncharacterized protein	54	2.31	2.7	1
W4ZQ59	Triticum aestivum	Uncharacterized protein	55	2.30	20.4	2
BLAST search: M8A407 60S acidic ribosomal protein P2B (identity: 100%)						
I3XHQ1	Triticum aestivum	Low molecular weight glutenin subunit LMW-9	56	2.18	32.9	72
A0A1D6C9L9	Triticum aestivum	Uncharacterized protein	57	2.18	4.1	1
BLAST search: W5GYX5 Glyceraldehyde-3-phosphate dehydrogenase (identity: 96.3%)						
P04729	Triticum aestivum	Gamma-gliadin B-I	58	2.16	27.3	33
L7VFZ3	Aegilops tauschii	Delta gliadin 1	59	2.15	3.7	1
V9TRL3	Triticum aestivum	High molecular weight glutenin subunit 1Dy protein	60	2.14	26.0	24
Q43723	Triticum aestivum	Trypsin/alpha-amylase inhibitor CMX1/CMX3	61	2.08	7.4	1
W5AKY9	Triticum aestivum	Uncharacterized protein	62	2.06	27.7	28
BLAST search: M7ZK46 12S seed storage globulin 1 (identity: 94.5%)						
W5AGK9	Triticum aestivum	Nucleoside diphosphate kinase	63	2.02	6.0	1
A0A1D5X2J6	Triticum aestivum	Uncharacterized protein	64	2.01	9.6	3
BLAST search: B8YG97 Avenin-like b11 (identity: 57.9%)						

V9P779	Triticum aestivum	LMW-m glutenin subunit 38	65	2.01	34.2	73
Q93XQ6	Triticum aestivum	Peptidyl-prolyl cis-trans isomerase	66	2.00	36.8	8
W5D003	Triticum aestivum	Uncharacterized protein	67	2.00	38.3	5
BLAST search: P10846 Alpha-amylase inhibitor WDAI-3 (identity: 95.5%)						
W5EB84	Triticum aestivum	Uncharacterized protein	68	2.00	2.1	2
BLAST search: W5EB84 Ribonuclease (identity: 100%)						
D0PRB5	Triticum aestivum	Profilin	69	2.00	9.9	1
W5H4P2	Triticum aestivum	Uncharacterized protein	70	2.00	3.4	1
BLAST search: M7Z0D7 DNA damage-inducible protein 1 (identity: 99.3%)						
A0A1D6C1I5	Triticum aestivum	Uncharacterized protein	71	2.00	5.7	1
M8BDU1	Aegilops tauschii	Basic 7S globulin	72	2.00	2.4	1
C5XET8	Sorghum bicolor	Uncharacterized protein	74	2.00	2.0	2
A0A287G1Y3	Hordeum vulgare subsp. vulgare	Uncharacterized protein	75	2.00	3.9	1
**Rye**						
**ω-secalins**						
A0A159KI56	Triticum aestivum	Omega-secalin	2	23.75	39.5	79
Q7M220	Secale cereale	Trypsin inhibitor	5	16.31	55.4	22
W6AW98	Triticum aestivum	High molecular weight glutenin subunit x	6	14.17	17.2	19
W6AW92	Triticum aestivum	High molecular weight glutenin subunit y	7	13.56	31.2	31
C3VWW0	Secale cereale	Dimeric alpha-amylase inhibitor	8	12.03	48.6	10
A0EPP1	Secale cereale	Puroindoline-a	9	8.40	37.4	10
C4NFQ2	Triticum aestivum	Omega secalin	10	7.35	43.2	25
Q45FA6	Secale cereale	Cereal-type amylase	12	6.02	33.9	5

		inhibitor (Fragment)				
R4JFB5	Triticum aestivum	Low-molecular-weight glutenin subunit (Fragment)	13	6.02	13.9	5
D2KFH1	Triticum aestivum	Avenin-like a4	14	6.02	21.5	9
A0A159KI54	Triticum aestivum	Omega-secalin	16	4.14	43.7	27
C4NFN6	Secale cereale	Omega secalin	17	4.04	42.0	69
Q43639	Secale cereale	Sec-1	18	4.00	44.0	71
C3VWW1	Secale cereale	Dimeric alpha-amylase inhibitor	19	4.00	48.6	12
Q6S5B1	Triticum turgidum subsp. durum	Alpha amylase inhibitor CM3	20	4.00	19.1	2
T1WIL2	Kengyilia kaschgarica	Dimeric alpha-amylase inhibitor	21	3.70	22.5	3
Q6J0X9	Aegilops biuncialis	Puroindoline a mutant	22	2.77	18.2	15
C4NFN5	Secale cereale	Omega secalin	23	2.30	37.3	45
A0EPP1	Secale cereale	Puroindoline a	25	2.08	37.4	10
A0A161E6H2	Triticum aestivum	Omega-secalin	26	2.00	40.3	44
C3VWW1	Secale cereale	Dimeric alpha-amylase inhibitor	27	2.00	40.9	11
W5AK86	Triticum aestivum	Uncharacterized protein	28	2.00	7.6	1
A0A1W5RMH3	Triticum aestivum	Elongation factor-1a (Fragment)	29	2.00	2.5	1
R7W8H0	Aegilops tauschii	Two-component response regulator ARR2	30	2.00	3.6	1
R7W7X6	Aegilops tauschii	Cytochrome P450 94A1	31	2.00	2.6	1
Q9S8H2	Secale cereale	Major BAKER'S ASTHMA allergen SEC C 1 (Fragment)	32	2.00	42.3	1
Q38794	Avena sativa	A.sativa seed storage protein	33	2.00	4.8	1
M0ZDF5	Hordeum vulgare subsp. vulgare	Uncharacterized protein	34	2.00	1.8	1
K4ABC8	Setaria italica	Uncharacterized protein	35	2.00	3.7	1

J3NDY0	Oryza brachyantha	Uncharacterized protein	36	2.00	6.6	1
B9FVQ0	Oryza sativa subsp. japonica	Uncharacterized protein	37	2.00	3.9	1
B8ABK4	Oryza sativa subsp. indica	Uncharacterized protein	38	2.00	1.2	1
BLAST search: Q8GU92 ABC transporter G family member 35 (identity: 93.8%)						
A0A287JQN1	Hordeum vulgare subsp. vulgare	Uncharacterized protein	39	2.00	8.4	1
A0A1Z5RAA3	Sorghum bicolor	Uncharacterized protein	40	2.00	30.3	1
A0A1D5S983	Triticum aestivum	Uncharacterized protein	42	2.00	10.4	1
BLAST search: E9NX12 Glutamate dehydrogenase (identity: 90.7%)						
A0A0E0L6A0	Oryza punctata	Uncharacterized protein	43	2.00	9.0	1
A0A0E0L4F6	Oryza punctata	Uncharacterized protein	44	2.00	2.9	1
A0A0E0GRU7	Oryza nivara	Uncharacterized protein	45	2.00	4.5	1
A0A0E0DCJ4	Oryza meridionalis	Uncharacterized protein	46	2.00	2.0	1
A0A0E0D3J0	Oryza meridionalis	Uncharacterized protein	47	2.00	3.3	1
**HMW-secalins**						
W6AW92	Triticum aestivum	High molecular weight glutenin subunit y	1	39.73	59.2	221
Q93WF0	Secale cereale	High molecular weight glutenin subunit x	2	30.31	41.5	109
W8NKZ9	Aegilops umbellulata	B-type farinin protein	4	10.70	39.3	27
W5G1U9	Triticum aestivum	Uncharacterized protein	5	8.08	38.4	5
Q6UJY8	Triticum turgidum	Globulin	6	7.30	28.0	11
W5D591	Triticum aestivum	Small ubiquitin-related modifier	8	6.92	40.6	4
Q40052	Hordeum vulgare	Glycine rich protein, RNA binding protein	9	6.88	38.7	5

ACT1	Sorghum bicolor	Actin-1	10	6.59	8.0	3
Q45FA6	Secale cereale	Cereal-type amylase inhibitor (Fragment)	11	6.33	43.2	4
Q6S5B1	Triticum turgidum subsp. durum	Alpha amylase inhibitor CM3	12	6.09	29.2	4
G8CN40	Secale cereale	Uncharacterized protein	14	5.06	6.3	3
BLAST search: M7YWL9 Selenium-binding protein 1 (identity: 99.4%)						
S4VIB2	Secale cereale x Triticum aestivum	Secaloindoline b	15	4.84	36.9	14
W5DPI4	Triticum aestivum	Uncharacterized protein	16	4.42	7.5	3
BLAST search: M7ZIE1 Calreticulin (identity: 86.8%)						
W5FMJ1	Triticum aestivum	Uncharacterized protein	17	4.36	32.3	5
C5WZL1	Sorghum bicolor	Peptide-methionin (R)-S-oxide reductase	18	4.24	23.9	4
W4ZRH9	Triticum aestivum	Glucose-1-phosphate adenylyltransferase	19	4.24	3.4	2
Q5DVL6	Hordeum vulgare subsp. vulgare	C2 domain-containing protein	20	4.23	13.8	4
W5IA32	Triticum aestivum	Formate dehydrogenase, mitochondrial	21	4.09	10.1	3
A0A0G3F720	Triticum aestivum	Endosperm transfer cell specific PR60	22	4.06	30.8	3
A0A2D0UFQ0	Triticum aestivum	X-type HMW-GS 1Slx2.2	23	4.00	8.2	42
I1H5K3	Brachypodium distachyon	40S ribosomal protein S21	24	4.00	31.7	5
C0PA67	Zea mays	Tubulin beta chain	25	3.81	4.9	2
R9XWG9	Triticum aestivum	LMW-GS	26	3.29	5.8	2
Q43639	Secale cereale	Sec1	27	2.92	14.3	7
A0A1E5W0T3	Dichanthelium oligosanthes	Elongation factor 1-alpha	28	2.75	3.1	1
Q2XSP8	Aegilops	Puroindoline a (Fragment)	29	2.73	20.8	2

	sharonensis					
Q9FEG4	Triticum turgidum subsp. durum	Protein disulfide isomerase (Fragment)	32	2.35	7.7	3
Q94IL5	Secale cereale	High molecular weight glutenin subunit x	33	2.32	13.8	34
T1WIL2	Kengyilia kaschgarica	Dimeric alpha-amylase inhibitor	34	2.23	7.2	1
W5HXX0	Triticum aestivum	Uncharacterized protein	35	2.22	5.1	1
BLAST search: M8A775 Elongation factor 1-gamma 2 (identity: 99.3%)						
R7WFE0	Aegilops tauschii	1,4-alpha-glucan-branching enzyme 2, chloroplastic/amyloplastic	36	2.19	1.0	1
Q9FR41	Secale cereale	Secalin	37	2.17	4.4	2
W5FTF6	Triticum aestivum	Uncharacterized protein	38	2.15	29.0	4
A0A3B5YYT3	Triticum aestivum	Peptide-methionine (R)-S-oxide reductase	39	2.12	11.4	2
A0A287E848	Hordeum vulgare subsp. vulgare	Uncharacterized protein	40	2.11	1.4	1
Q7M220	Secale cereale	Trypsin inhibitor	41	2.08	15.7	1
W5ETW5	Triticum aestivum	UDP-glucose 6-dehydrogenase	43	2.07	3.1	1
H6UQP6	Elymus libanoticus	High molecular weight subunit of glutenin	44	2.06	13.6	21
R7W9Z9	Aegilops tauschii	Uncharacterized protein	45	2.06	7.9	1
W5ERI9	Triticum aestivum	Non-specific lipid-transfer protein	46	2.04	9.7	1
M8A1S2	Triticum urartu	Trypsin/alpha-amylase inhibitor CMX1/CMX3	47	2.04	6.2	1
W5B5R3	Triticum aestivum	Sucrose synthase	48	2.03	1.3	1
W4ZR59	Triticum aestivum	Mitochondrial fission 1 protein	49	2.03	5.5	1
A5HMG1	Triticum aestivum	HMW glutenin subunit 1Bx13	50	2.01	3.1	11
AVLA4	Triticum aestivum	Avenin-like a4	52	2.01	14.0	2

Q8L808	Triticum aestivum	Putative cytochrome c oxidase subunit	53	2.01	6.4	1
E7DVE3	Aegilops kotschyi	High molecular weight glutenin subunit Ux2.3	54	2.00	8.0	28
A0A1D5S923	Triticum aestivum	Globulin 1	55	2.00	22.8	8
B6VCM5	Secale cereale	Chloroplast putative glucose-1-phosphate adenylyltransferase large subunit 1 (Fragment)	56	2.00	19.6	2
A0A287JQN1	Hordeum vulgare subsp. vulgare	Uncharacterized protein	57	2.00	8.4	1
W5G3X9	Triticum aestivum	Uncharacterized protein	59	2.00	9.4	1
BLAST search: M7ZI24 40S ribosomal protein S20 (identity: 97.5%)						
W5FEZ3	Triticum aestivum	40S ribosomal protein S12	60	2.00	6.9	1
W5E8X2	Triticum aestivum	Uncharacterized protein	61	2.00	18.5	1
BLAST search: M7ZP32 40S ribosomal protein S28 (identity: 100%)						
W5DYD1	Triticum aestivum	Uncharacterized protein	62	2.00	2.7	1
W5AMQ8	Triticum aestivum	Uncharacterized protein	63	2.00	2.1	1
W4ZTE4	Triticum aestivum	Uncharacterized protein	64	2.00	3.8	1
M8CU71	Aegilops tauschii	Vesicle transport v-SNARE 13	65	2.00	8.1	1
M8C8R9	Aegilops tauschii	Uncharacterized protein	66	2.00	5.6	1
M7ZAR5	Triticum urartu	Uncharacterized protein	67	2.00	4.0	1
**γ-75k-secalins**						
E5KZQ2	Secale cereale	75k gamma secalin	1	53.31	43.7	165
B9A8E2	Triticum aestivum	Protein disulfide-isomerase	2	32.01	41.0	30
R7W8L3	Aegilops tauschii	Uncharacterized protein	4	18.00	37.0	12

BLAST search: Q9ZSR6 Heat shock protein HSP26 (identity: 100%)						
A0A1D6RH21	Triticum aestivum	Uncharacterized protein	5	16.35	28.2	10
Q7M220	Secale cereale	Trypsin inhibitor	6	15.01	61.2	23
Q7M1Z3	Secale cereale	rRNA N-glycosidase	7	14.81	23.2	9
W6AW92	Triticum aestivum	High molecular weight glutenin subunit y	8	14.17	27.3	39
A0A1D5U769	Triticum aestivum	Sucrose synthase	9	13.81	13.2	10
M7ZK46	Triticum urartu	12S seed storage globulin 1	10	13.58	15.2	16
M8C1W9	Aegilops tauschii	L-ascorbate peroxidase 2, cytosolic	11	13.35	39.4	9
W5E2W7	Triticum aestivum	40S ribosomal protein SA	12	12.83	28.9	8
R7W001	Aegilops tauschii	1-Cys peroxiredoxin PER1	14	11.70	33.0	6
E5KZQ6	Secale cereale	75k gamma secalin	15	11.52	74.8	153
C3VWW0	Secale cereale	Dimeric alpha-amylase inhibitor	16	11.03	54.9	15
CALM	Triticum aestivum	Calmodulin	17	10.13	54.4	10
Q96185	Triticum aestivum	Superoxide dismutase	18	10.10	26.4	5
Q93XQ6	Triticum aestivum	Peptidyl-prolyl cis-trans isomerase	19	9.61	46.2	14
Q8VX48	Triticum aestivum	Phosphoglucomutase (Fragment)	20	9.34	9.1	5
Q6KE43	Secale cereale	Xylanase inhibitor (Fragment)	23	8.68	20.3	6
W5I4U0	Triticum aestivum	Uncharacterized protein	24	8.54	35.1	5
BLAST search: M7ZNI1 Putative Calcium-binding protein CML7 (identity: 98.7%)						
Q6BCK0	Secale cereale	Puroindoline b	25	8.52	43.9	14
M8BDK9	Aegilops tauschii	Alanine aminotransferase 2	26	8.08	9.6	6
Q94IK9	Secale cereale	High molecular weight glutenin subunit x	27	7.95	10.2	15
W8NKZ9	Aegilops umbellulata	B-type farinin protein	28	7.76	29.6	12

M8BDX3	Aegilops tauschii	Uncharacterized protein	29	7.28	14.9	5
M8B1Z5	Aegilops tauschii	Lactoylglutathione lyase	30	7.16	15.1	4
Q9SAU8	Triticum aestivum	HSP70	31	7.15	5.7	3
C1J958	Secale cereale	Fructose-bisphosphate aldolase	32	7.06	12.9	4
I1ITE4	Brachypodium distachyon	Uncharacterized protein	33	6.84	20.3	4
A0A1D5TFS7	Triticum aestivum	Uncharacterized protein	34	6.67	26.8	4
Q45FA6	Secale cereale	Cereal-type amylase inhibitor (Fragment)	35	6.66	30.5	4
W5FZ62	Triticum aestivum	Uncharacterized protein	36	6.56	15.0	5
BLAST search: P93693 Serpin Z1B (identity: 100%)						
W4ZRH9	Triticum aestivum	Glucose-1-phosphate adenylyltransferase	37	6.44	7.9	3
A0A1D6SB15	Triticum aestivum	Uncharacterized protein	38	6.40	7.9	3
A0EPP1	Secale cereale	Puroindoline a	39	6.29	36.7	11
W5CY88	Triticum aestivum	Peroxiredoxin	40	6.28	19.1	4
W5FEQ2	Triticum aestivum	Uncharacterized protein	42	6.23	11.8	3
BLAST search: M7YC85 Prohibitin-2 (identity: 92.5%)						
F2D1G5	Hordeum vulgare subsp. vulgare	Predicted protein	43	6.12	8.3	3
Q6E5A5	Hordeum vulgare subsp. vulgare	Plastidial ADP-glucose transporter	44	6.10	6.4	3
Q6UJY8	Triticum turgidum	Globulin	45	6.06	17.3	4
AMYB	Secale cereale	Beta-amylase (Fragment)	46	6.04	18.0	4
W5EF79	Triticum aestivum	Uncharacterized protein	47	6.04	23.0	3
I1HBS1	Brachypodium	Uncharacterized protein	48	6.00	24.2	4

	distachyon					
BLAST search: P12810 16.9 kDa class I heat shock protein 1 (identity: 85.7%)						
W5HG94	Triticum aestivum	Uncharacterized protein	49	6.00	8.9	3
A0A1D5UB33	Triticum aestivum	Uncharacterized protein	50	6.00	14.9	3
M8CBB2	Aegilops tauschii	Peptidylprolyl isomerase	51	5.97	4.5	3
Q3S4I2	Triticum monococcum	Pathogenesis-related 5	52	5.65	36.9	7
W5ENP1	Triticum aestivum	Uncharacterized protein	53	5.60	19.2	3
Q9FSI8	Hordeum vulgare	Cold-regulated protein	54	5.60	21.3	3
F2EKR6	Hordeum vulgare subsp. vulgare	Predicted protein	55	5.55	4.3	4
M0Z0Z6	Hordeum vulgare subsp. vulgare	Uncharacterized protein	56	5.49	15.3	4
A0A1D6B171	Triticum aestivum	Uncharacterized protein	57	5.38	33.8	10
BLAST search: P01083 Alpha-amylase inhibitor 0.28 (identity: 70.1%)						
M8A7N9	Triticum urartu	Universal stress protein A-like protein	58	5.20	18.2	3
W5G3G1	Triticum aestivum	Uncharacterized protein	59	5.13	14.4	4
BLAST search: M8ATI4 Homocysteine S-methyltransferase 3 (identity: 98.7%)						
A0A1D5WIE9	Triticum aestivum	Uncharacterized protein	60	5.05	5.6	3
BLAST search: M7YUQ6 Aspartic proteinase oryzasin-1 (identity: 91.4%)						
M0Z9S4	Hordeum vulgare subsp. vulgare	Uncharacterized protein	61	4.96	7.2	3
R9W924	Triticum aestivum	ER molecular chaperone	62	4.81	3.3	3
M7Z0L4	Triticum urartu	Glutelin type-A 1	63	4.64	7.1	2
A0A1D6APE7	Triticum aestivum	Uncharacterized protein	64	4.49	18.9	4

BLAST search: M8B1U5 Putative invertase inhibitor (identity: 92.2%)

Q308L7	Secale cereale	Eukaryotic translation initiation factor 5A	65	4.32	13.7	2
R7W8V8	Aegilops tauschii	Tubulin alpha chain	66	4.29	8.0	3
W5EKI0	Triticum aestivum	Beta-amylase (Fragment)	67	4.29	9.1	5
R4JFA4	Triticum aestivum	Low-molecular-weight glutenin subunit (Fragment)	68	4.24	10.5	5
W5EB84	Triticum aestivum	Ribonuclease	69	4.23	2.0	2
M0USC9	Hordeum vulgare subsp. vulgare	Aspartate aminotransferase	70	4.22	8.1	3
AVLA4	Triticum aestivum	Avenin-like a4	71	4.22	21.5	20
A0A287E848	Hordeum vulgare subsp. vulgare	Uncharacterized protein	72	4.20	2.5	2

BLAST search: M7ZQ95 1,4-alpha-glucan-branching enzyme (identity: 94.6%)

M8A2J7	Triticum urartu	Ubiquilin-1	73	4.14	5.3	3
Q6S5B1	Triticum turgidum subsp. durum	Alpha amylase inhibitor CM3	74	4.10	19.1	6
I1I6D9	Brachypodium distachyon	Uncharacterized protein	75	4.08	10.9	2
R7W3W0	Aegilops tauschii	Cytochrome b-c1 complex subunit 7	76	4.06	15.1	2
B6UE15	Zea mays	Soluble inorganic pyrophosphatase	77	4.04	12.2	2
W5B2J4	Triticum aestivum	Uncharacterized protein	78	4.02	12.4	2
W5I4Y2	Triticum aestivum	Glucose-1-phosphate adenylyltransferase	79	4.01	3.7	2
W5EMF3	Triticum aestivum	Ubiquitin-fold modifier-conjugating enzyme 1	80	4.01	20.1	3
M0YHS1	Hordeum vulgare subsp. vulgare	Uncharacterized protein	81	4.00	5.5	2

W5HXX0	Triticum aestivum	Uncharacterized protein	82	4.00	4.8	2
BLAST search: M8A775 Elongation factor 1-gamma 2 (identity: 98.8%)						
W5CQE4	Triticum aestivum	Uncharacterized protein	83	4.00	10.0	2
M8BAK8	Aegilops tauschii	Uncharacterized protein	84	4.00	13.8	2
BLAST search: Q43723 trypsin/alpha-amylase inhibitor CMX1/CMX3 (identity: 92.6%)						
Q9AXR9	Secale cereale	31.7 kDa class I endochitinase-antifreeze protein	85	4.00	3.4	1
R7WAR0	Aegilops tauschii	Isocitrate dehydrogenase [NADP]	86	3.88	4.6	2
F4ZL26	Secale strictum subsp. africanum	Alpha-gliadin storage protein	87	3.83	17.6	4
Q9SDK4	Oryza sativa subsp. japonica	GTP-binding protein	88	3.70	10.9	2
W5BVL3	Triticum aestivum	Uncharacterized protein	89	3.64	7.5	2
BLAST search: M7YGC2 Putative quinone-oxidoreductase-like protein (identity*: 91.2%)*						
W5GVY4	Triticum aestivum	Uncharacterized protein	90	3.49	18.8	2
U5S8M6	Dendrocalamus latiflorus	Elongation factor 1-alpha	91	3.48	5.1	2
F2DIQ9	Hordeum vulgare subsp. vulgare	Predicted protein	92	3.42	4.5	2
M8AJF7	Triticum urartu	Uncharacterized protein	93	3.39	30.8	4
BLAST search: Q05806 Type-5 thionin (identity: 95.4%)						
T1NAD0	Triticum urartu	Malate dehydrogenase	94	3.38	8.8	2
F2E5Q1	Hordeum vulgare subsp. vulgare	Profilin	95	3.37	35.1	5
Q6J0X9	Aegilops biuncialis	Puroindoline a mutant	96	3.35	18.2	14
W5GYX5	Triticum	Glyceraldehyde-3-phosphate	97	3.28	11.0	3

	aestivum	dehydrogenase				
W4ZR59	Triticum aestivum	Mitochondrial fission 1 protein	98	3.28	13.4	2
M8BTX4	Aegilops tauschii	12-oxophytodienoate reductase 2	99	3.23	10.3	2
M7YW45	Triticum urartu	Vacuolar-sorting receptor 1	100	3.17	2.7	2
S4ULQ4	Secale cereale	Dimeric alpha-amylase inhibitor (Fragment)	101	3.15	45.7	9
A0A287WJQ6	Hordeum vulgare subsp. vulgare	Uncharacterized protein	102	3.08	20.4	3
BLAST search: M7ZWD9 Nuclear transport factor 2 (identity: 99.0%)						
W5AXT4	Triticum aestivum	Uncharacterized protein	103	3.05	4.6	2
BLAST search: M8B153 Coproporphyrinogen-III oxidase (identity: 99.3%)						
A0A0E3T7B1	Secale cereale	Putative elongation factor 1	104	2.99	1.2	1
W5EF42	Triticum aestivum	Uncharacterized protein	105	2.99	5.1	2
BLAST search: M8A214 Vicilin-like antimicrobial peptides 2-2 (identity: 93.2%)						
F2D1U7	Hordeum vulgare subsp. vulgare	UMP-CMP kinase	106	2.93	8.5	2
A0EPP1	Secale cereale	Puroindoline-a	107	2.85	36.7	11
F2CS51	Hordeum vulgare subsp. vulgare	Pyruvate kinase	108	2.76	1.9	1
Q8H530	Oryza sativa subsp. japonica	Os07g0485100 protein	109	2.72	6.1	2
W5G6L7	Triticum aestivum	Uncharacterized protein	110	2.71	2.2	1
BLAST search: M8ATW1 Betaine aldehyde dehydrogenase 1 (identity: 97.3%)						
Q6KE41	Secale cereale	Xylanase inhibitor	111	2.64	13.9	6
M8A392	Triticum urartu	Uncharacterized protein	112	2.62	4.0	1
BLAST search: M7ZAQ9 Oxalate oxidase 2 (identity: 100%)						
W5DQS5	Triticum aestivum	Uncharacterized protein	113	2.62	4.0	1

H6ULI8	Secale strictum subsp. africanum	75k gamma secalin	114	2.60	47.7	160
F2D284	Hordeum vulgare subsp. vulgare	Protein disulfide-isomerase	115	2.55	37.2	27
W5I139	Triticum aestivum	Uncharacterized protein	116	2.40	3.0	1
C4P5P7	Triticum dicoccoides	Monomeric alpha-amylase inhibitor	117	2.32	13.9	3
W5DLH0	Triticum aestivum	Uncharacterized protein	118	2.29	3.9	1
W5DYD1	Triticum aestivum	Uncharacterized protein	119	2.28	6.5	2
W4ZQ59	Triticum aestivum	Uncharacterized protein	120	2.26	8.9	1
BLAST search: M8A407 60S acidic ribosomal protein P2B (identity: 100%)						
Q43660	Triticum aestivum	Heat shock protein 17.3	121	2.25	9.6	1
M7ZGK1	Triticum urartu	Uncharacterized protein	122	2.21	7.5	1
M0WZI8	Hordeum vulgare subsp. vulgare	Uncharacterized protein	123	2.20	2.4	1
BLAST search: M8A2G0 ADP,ATP carrier protein, mitochondrial (identity: 97.9%)						
M8BT11	Aegilops tauschii	Stromal 70 kDa heat shock-related protein, chloroplastic	124	2.18	1.7	1
M8CA41	Aegilops tauschii	Adenylate kinase A	125	2.16	16.6	3
I1HCH9	Brachypodium distachyon	NADH-cytochrome b5 reductase	126	2.16	3.9	1
A0A1D6BSI1	Triticum aestivum	Uncharacterized protein	127	2.15	12.1	3
M7Z7R6	Triticum urartu	Uncharacterized protein	128	2.15	4.5	1
F2E8C1	Hordeum vulgare subsp. vulgare	Predicted protein	129	2.13	9.1	1
F2E9N0	Hordeum vulgare subsp.	Predicted protein	130	2.08	11.9	15

	vulgare					
BLAST search: M7ZK46 12S seed storge globulin 1 (identity: 86.6%)						
M7YF70	Triticum urartu	Uncharacterized protein	131	2.08	5.4	1
BLAST search: Q8LRM8 Translationally-controlledtumor protein homolog (identity: 99.4%)						
W5G4R7	Triticum aestivum	Pyrophosphate--fructose 6-phosphate 1-phosphotransferase subunit alpha	132	2.08	1.5	1
Q8LK23	Triticum aestivum	Peroxidase	133	2.08	3.4	1
A0A1D5XGF4	Triticum aestivum	Beta-amylase	135	2.07	9.1	5
F2DSB7	Hordeum vulgare subsp. vulgare	Predicted protein	136	2.06	4.7	2
BLAST search: M7ZRF8 Acid phosphatase 1 (identity: 88.5%)						
W5BGN8	Triticum aestivum	Uncharacterized protein	137	2.05	2.1	1
Q0J4K2	Oryza sativa subsp. japonica	Lipoxygenase	138	2.05	0.8	1
W5FPN2	Triticum aestivum	Uncharacterized protein	139	2.04	2.0	1
W5E8D7	Triticum aestivum	1,2-dihydroxy-3-keto-5-methylthiopentene dioxygenase	140	2.04	5.4	1
W5CKU7	Triticum aestivum	Uncharacterized protein	141	2.03	5.1	1
M8CZ52	Aegilops tauschii	Farnesyl pyrophosphate synthetase	142	2.03	3.0	1
Q9LKM0	Lolium perenne	Nucleoside diphosphate kinase	143	2.02	6.0	1
M8CGQ1	Aegilops tauschii	Uncharacterized protein	144	2.02	8.9	1
M8BYD7	Aegilops tauschii	Purple acid phosphatase	145	2.02	2.2	1
W5EFT2	Triticum aestivum	Uncharacterized protein	146	2.01	3.9	1
T1N6W0	Triticum	Uncharacterized protein	147	2.01	10.4	1

	urartu					
BLAST search: A0A3B6HWM1 Alanine--tRNA ligase (identity: 100%)						
B6SGY5	Zea mays	RNA binding protein	148	2.01	9.5	2
K7WM32	Secale cereale	75k gamma secalin	149	2	38.4	161
E5KZQ0	Secale cereale	75k gamma secalin	150	2	34.8	162
Q9AXH7	Triticum turgidum subsp. durum	1-Cys peroxiredoxin	151	2	33.5	6
I1H7J7	Brachypodium distachyon	Uncharacterized protein	152	2	21.3	6
BLAST search: Q00445 Small heat shock protein (identity: 86.0%)						
W5A619	Triticum aestivum	Uncharacterized protein	153	2	54.4	10
BLAST search: P62162 Calmodulin (identity: 99.3%)						
C3VWW1	Secale cereale	Dimeric alpha-amylase inhibitor	154	2	54.9	13
H8Y0P2	Secale cereale subsp. tetraploidum	Gamma prolamin (Fragment)	155	2	12.8	15
J7I265	Aegilops speltoides	Gamma-gliadin (Fragment)	157	2	6.7	3
M0UGW6	Hordeum vulgare subsp. vulgare	Uncharacterized protein	158	2	6.5	1
W5D6S5	Triticum aestivum	S-formylglutathione hydrolase	160	2	5.9	1
T1N7I1	Triticum urartu	Uncharacterized protein	161	2	5.5	1
P83207	Triticum aestivum	Chymotrypsin inhibitor WCI	162	2	16.8	1
P16968	Hordeum vulgare	Alpha-amylase inhibitor BMAI-1 (Fragment)	163	2	7.5	1
K3XLS0	Setaria italica	Uncharacterized protein	164	2	5.4	1
W5IC49	Triticum aestivum	Uncharacterized protein	165	2	3.3	1
W5I0B0	Triticum aestivum	Uncharacterized protein	166	2	2.4	1
W5GBF3	Triticum aestivum	Uncharacterized protein	168	2	3.1	1
W5FP48	Triticum	Uncharacterized protein	169	2	4.3	1

	aestivum					
W5EX16	Triticum aestivum	Uncharacterized protein	170	2	11.1	1
P46226	Secale cereale	Triosephosphate isomerase, cytosolic	172	2	4.7	1
W5AL85	Triticum aestivum	Phosphoinositide phospholipase C	174	2	1.5	1
W4ZU05	Triticum aestivum	Uncharacterized protein	175	2	3.5	1
R7WDL2	Aegilops tauschii	Isopentenyl-diphosphate Delta-isomerase I	176	2	6.2	1
R7W2G4	Aegilops tauschii	Formate--tetrahydrofolate ligase	177	2	1.6	1
Q9S8H2	Secale cereale	Major BAKER'S ASTHMA allergen SEC C 1 (Fragment)	178	2	42.3	1
A2SXR9	Triticum aestivum	Uricase	179	2	3.3	1
D2KZ09	Triticum aestivum	Seed protein B32E	180	2	2.0	1
F6MIX5	Secale cereale	Purple acid phosphatase	181	2	1.9	1
A0A1D6RYC2	Triticum aestivum	Uncharacterized protein	182	2	3.0	1
Q69Y17	Oryza sativa subsp. japonica	Putative PrMC3	183	2	4.4	1
N1QP80	Aegilops tauschii	Translocon-associated protein subunit beta	184	2	6.2	1
A0A1D5ZWI3	Triticum aestivum	Uncharacterized protein	185	2	5.1	1
A0A1D5SY85	Triticum aestivum	Uncharacterized protein	187	2	4.2	1
M7YA95	Triticum urartu	Putative 6-phosphogluconolactonase 2	188	2	3.3	1
K3Y8W0	Setaria italica	Uncharacterized protein	189	2	4.5	1
B8BMJ7	Oryza sativa subsp. indica	Uncharacterized protein	190	2	4.9	1
A0A1W0VU20	Sorghum bicolor	Uncharacterized protein	191	2	3.2	1
A0A1D5YMB2	Triticum aestivum	Uncharacterized protein	192	2	2.7	1

**γ-40k-secalins**						
A0A1D5U769	Triticum aestivum	Sucrose synthase	1	28.53	23.0	25
W5AH12	Triticum aestivum	Uncharacterized protein	2	27.76	48.8	23
BLAST search: M8ASF1 Actin-2 (identity: 100%)						
H8Y0K4	Secale cereale	Gamma prolamin	4	24.32	54.8	83
B9A8E2	Triticum aestivum	Protein disulfide-isomerase	5	21.67	25.6	13
A0A287HMM0	Hordeum vulgare subsp. vulgare	Uncharacterized protein	7	19.77	38.4	18
BLAST search: M7ZLT6 Alpha-1,4-glucan-protein synthase (identity: 98.3%)						
Q41593	Triticum aestivum	Serpin-Z1A	8	17.61	29.9	13
A0A0E3T7B1	Secale cereale	Putative elongation factor 1	10	16.20	6.9	7
W6AW92	Triticum aestivum	High molecular weight glutenin subunit y	11	15.88	23.3	18
A0A1D6C5Y4	Triticum aestivum	Uncharacterized protein	12	15.59	29.4	9
W5AFW9	Triticum aestivum	Uncharacterized protein	13	15.00	43.3	19
BLAST search: M8AMK8 Actin-7 (identity: 100%)						
M8BW46	Aegilops tauschii	RNA-binding protein Nova-1	14	14.69	22.2	6
O04074	Triticum aestivum	Starch branching enzyme 1	15	13.70	9.9	7
W5AUU7	Triticum aestivum	Uncharacterized protein	16	13.61	30.9	10
M8BDX3	Aegilops tauschii	Uncharacterized protein	17	13.31	26.6	8
K7WZB8	Secale cereale	75k gamma secalin	18	13.24	23.8	28
E0WC53	Triticum aestivum	WALI7	19	12.75	34.1	11
W5HXV0	Triticum aestivum	Uncharacterized protein	20	12.62	19.1	5
W5IA32	Triticum aestivum	Formate dehydrogenase, mitochondrial	22	12.17	18.6	7
N1QXI7	Aegilops tauschii	Stromal 70 kDa heat shock-related protein, chloroplastic	23	12.15	10.4	6

N1QTF4	Aegilops tauschii	DEAD-box ATP-dependent RNA helicase 37	24	12.11	6.0	6
R7W8L9	Aegilops tauschii	Glutamate-1-semialdehyde 2,1-aminomutase, chloroplastic	25	12.09	17.2	7
M8A4T0	Triticum urartu	V-type proton ATPase subunit C	26	12.00	15.5	6
A0A287X6Z0	Hordeum vulgare subsp. vulgare	Uncharacterized protein	28	11.46	24.7	8
F2DSA1	Hordeum vulgare subsp. vulgare	Predicted protein	29	11.42	52.7	11
M8BB60	Aegilops tauschii	Prohibitin-1, mitochondrial	30	11.02	44.5	9
Q84UC6	Hordeum vulgare subsp. vulgare	Pyrroline-5-carboxylate reductase	32	10.89	28.7	6
Q9M5G3	Hordeum vulgare subsp. vulgare	Translationally-controlled tumor protein homolog	33	10.87	25.6	5
W5EKI0	Triticum aestivum	Beta-amylase	34	10.81	15.3	9
B2ZES2	Triticum aestivum	Small heat shock protein 16.9 kDa	35	10.56	43.1	7
S4VIB2	Secale cereale x Triticum aestivum	Secaloindoline b	36	10.35	43.6	16
Q7M1Z3	Secale cereale	rRNA N-glycosidase	37	10.18	23.9	6
H9AXB3	Triticum aestivum	Serpin-n3:2	38	10.00	17.1	7
P46226	Secale cereale	Triosephosphate isomerase, cytosolic	39	9.85	26.5	5
Q7M220	Secale cereale	Trypsin inhibitor	40	9.29	54.5	8
W5AK05	Triticum aestivum	Glucose-6-phosphate isomerase	41	9.12	9.2	5
M0V255	Hordeum vulgare subsp. vulgare	Uncharacterized protein	42	9.04	20.9	5
W5H4V7	Triticum aestivum	Phosphoglycerate kinase	43	9.01	11.2	4

W5E031	Triticum aestivum	Uncharacterized protein	44	8.69	16.7	5
W8NKZ9	AEGUM	B-type farinin protein	45	8.50	26.1	7
M8A7A7	Triticum urartu	26S proteasome non-ATPase regulatory subunit RPN12A	46	8.35	16.5	5
A0A1D5WIE9	Triticum aestivum	Uncharacterized protein	47	8.29	12.3	8
P30271	Secale cereale	Beta-amylase (Fragment)	48	8.26	21.6	5
M0UP66	Hordeum vulgare subsp. vulgare	Uncharacterized protein	49	8.16	13.8	4
Q43661	Triticum aestivum	Wali7 protein (Fragment)	50	8.09	18.5	4
C3VWW0	Secale cereale	Dimeric alpha-amylase inhibitor	51	8.00	40.8	5
A0A1D5X277	Triticum aestivum	Uncharacterized protein	52	7.88	10.9	4
M5ET52	Triticum aestivum	GT47_2D	53	7.84	12:4	5
W5GBF3	Triticum aestivum	Uncharacterized protein	54	7.83	9.6	4
M8AMF7	Aegilops tauschii	Universal stress protein A-like protein	55	7.48	39.2	7
Q8LK23	Triticum aestivum	Peroxidase	56	7.47	12.3	4
R9W924	Triticum aestivum	ER molecular chaperone	58	7.35	6.8	4
Q94IK9	Secale cereale	High molecular weight glutenin subunit x	59	7.18	10.9	8
M0WXC5	Hordeum vulgare subsp. vulgare	Uncharacterized protein	60	7.14	16.2	3
F2E9N0	Hordeum vulgare subsp. vulgare	Predicted protein	61	7.09	11.4	11
BLAST search: M7ZK46 12S seed storage globulin 1 (identity: 86.6%)						
N1QZ20	Aegilops tauschii	Methylmalonate-semialdehyde dehydrogenase (Acylating), mitochondrial	62	7.06	6.7	3
A0A1D5WF54	Triticum aestivum	Uncharacterized protein	63	6.94	6.0	3

Q93XQ6	Triticum aestivum	Peptidyl-prolyl cis-trans isomerase	64	6.90	28.7	7
W5BKC3	Triticum aestivum	Uncharacterized protein	65	6.77	12.3	6
F2EA18	Hordeum vulgare subsp. vulgare	Predicted protein (Fragment)	66	6.75	9.1	3
A0A1D5RTS1	Triticum aestivum	Malate dehydrogenase	67	6.69	6.0	3
D4N8D8	Secale cereale x Triticum turgidum subsp. durum	Carbonic anhydrase	68	6.66	15.8	5
W5DLH0	Triticum aestivum	Uncharacterized protein	69	6.58	10.5	5
W5AVS5	Triticum aestivum	Uncharacterized protein	70	6.35	11.3	3
BLAST search: M8A0Z1 Putative 26S proteasome non-ATPase regulatory subunit 7 (identity: 96.3%)						
A0A1D6S9Y1	Triticum aestivum	Uncharacterized protein	71	6.30	16.8	5
W5D0E3	Triticum aestivum	Fructose-bisphosphate aldolase	72	6.24	8.7	3
Q6UJY8	Triticum turgidum	Globulin	73	6.17	17.3	5
A0A1E5WJH2	Dichanthelium oligosanthes	Ubiquitin-conjugating enzyme E2 28	74	6.13	33.8	11
M7ZL68	Triticum urartu	Universal stress protein A-like protein	75	6.02	19.3	3
U3N1P5	Triticum turgidum	Glutamine synthetase	76	6.00	9.1	3
Q5KSN7	Leymus chinensis	Betaine aldehyde dehydrogenase	77	5.94	11.2	5
Q3S4I1	Triticum aestivum	Eukaryotic translation initiation factor 5A	78	5.85	21.1	3
Q8VX48	Triticum aestivum	Phosphoglucomutase (Fragment)	79	5.81	7.4	4
A0A287S5K9	Hordeum vulgare subsp. vulgare	UTP--glucose-1-phosphate uridylyltransferase	80	5.68	4.7	2

W5AQ87	Triticum aestivum	Uncharacterized protein	81	5.67	31.0	4
BLAST search: A0A287ITX4 Isocitrate dehydrogenase [NADP] (identity: 90.2%)						
W5CAC3	Triticum aestivum	Uncharacterized protein	82	5.56	12.4	2
G4XH71	Secale cereale x Triticum turgidum subsp. Durum	Peptidyl-prolyl cis-trans isomerase	83	5.56	28:7	7
W4ZSW9	Triticum aestivum	Serine/threonine-protein phosphatase	84	5.56	7.5	3
A0A1D6BQZ0	Triticum aestivum	Tubulin beta chain	85	5.54	9.9	3
W5I774	Triticum aestivum	Sucrose synthase	86	5.42	7.2	8
Q75RZ2	Triticum aestivum	Putative caffeoyl CoA O-methyltransferase (Fragment)	87	5.32	8.4	3
Q6J0X9	Aegilops biuncialis	Puroindoline a mutant	88	5.21	16.9	5
T1NAK2	Triticum urartu	Uncharacterized protein	89	5.20	12.2	4
BLAST search: A0A140KPU9 GA3ox-A3 protein (identity: 97.0%)						
W5G919	Triticum aestivum	Uncharacterized protein	90	5.18	8.8	3
W5G208	Triticum aestivum	Malate dehydrogenase	91	5.17	6.4	3
W5BVL3	Triticum aestivum	Uncharacterized protein	92	5.02	10.1	2
Q6E5A5	Hordeum vulgare subsp. vulgare	Plastidial ADP-glucose transporter	93	4.99	4.2	2
W5BNI1	Triticum aestivum	Aldose 1-epimerase	94	4.95	9.3	3
A0A287SV63	Hordeum vulgare subsp. vulgare	Uncharacterized protein	95	4.82	5.5	3
M7ZUX7	Triticum urartu	Cytochrome b-c1 complex subunit Rieske, mitochondrial	96	4.80	8.3	2
Q5EWZ1	Triticum	Actin	97	4.78	46.2	19

	aestivum					
A0A1D5YA21	Triticum aestivum	Uncharacterized protein	98	4.74	13.5	4
W5FSY8	Triticum aestivum	Homoserine dehydrogenase	99	4.69	7.8	3
F4ZL28	Secale strictum subsp. africanum	Alpha-gliadin storage protein	100	4.63	16.7	3
W5ERM8	Triticum aestivum	Proteasome subunit beta type	101	4.62	18.5	3
W5CPG2	Triticum aestivum	Uncharacterized protein	102	4.60	2.6	2
BLAST search: M7Z099 Glutaminyl-tRNA synthetase (identity: 99.1%)						
W5ELU8	Triticum aestivum	Uncharacterized protein	103	4.58	20.2	4
A0A1D5WE06	Triticum aestivum	Uncharacterized protein	104	4.48	6.3	2
A0A1D6ABF1	Triticum aestivum	Uncharacterized protein	105	4.37	10.1	5
W5CY88	Triticum aestivum	Uncharacterized protein	106	4.36	12.3	2
W5GVY4	Triticum aestivum	Uncharacterized protein	107	4.30	18.8	3
W5DQS5	Triticum aestivum	Uncharacterized protein	108	4.20	11.0	2
W5I4R6	Triticum aestivum	Uncharacterized protein	109	4.17	7.1	2
Q1EMA2	Secale cereale	Glucan endo-1,3-beta-D-glucosidase	110	4.14	7.1	2
M7ZPJ4	Triticum urartu	26S proteasome non-ATPase regulatory subunit 14	111	4.14	13.2	3
M8CZ52	Aegilops tauschii	Farnesyl pyrophosphate synthetase	112	4.13	5.2	2
Q9SAU8	Triticum aestivum	HSP70	113	4.07	3.7	2
M8C8G6	Aegilops tauschii	Glyceraldehyde-3-phosphate dehydrogenase	114	4.06	13.3	4
P36183	Hordeum vulgare	Endoplasmin homolog	115	4.06	2.5	2

A0A1D5RU62	Triticum aestivum	Uncharacterized protein	116	4.05	12.8	2
Q7XYE1	Triticum aestivum	Tat binding protein (Fragment)	117	4.04	11.8	2
M8B7Y1	Aegilops tauschii	Putative 6-phosphogluconolactonase 4, chloroplastic	118	4.04	6.5	2
W5AC28	Triticum aestivum	Uncharacterized protein	119	4.04	7.7	2
W5B7W5	Triticum aestivum	Caleosin	120	4.02	14.3	3
A0A1D5XGF4	Triticum aestivum	Beta-amylase	121	4.00	14.3	6
Q41602	Triticum turgidum subsp. durum	Gamma gliadin (Fragment)	122	4.00	16.4	10
B9VRH9	Triticum macha	Gamma-gliadin	123	4.00	15.4	10
M8AXM7	Aegilops tauschii	Pectin acetylesterase	124	4.00	6.0	2
M8BI24	Aegilops tauschii	E3 ubiquitin-protein ligase RGLG2	125	4.00	7.0	2
M7ZGI6	Triticum urartu	Oxalate oxidase 2	126	4.00	11.6	2
F2CSC4	Hordeum vulgare subsp. vulgare	Predicted protein	127	4.00	7.5	2
A0A1D5X6C4	Triticum aestivum	Uncharacterized protein	128	4.00	11.3	2
W5G4R7	Triticum aestivum	Pyrophosphate--fructose 6-phosphate 1-phosphotransferase subunit alpha	129	4.00	3.3	2
W5G3G1	Triticum aestivum	Uncharacterized protein	130	4.00	7.9	2
Q9AXH7	Triticum turgidum subsp. durum	1-Cys peroxiredoxin	131	4.00	8.7	2
Q10MF7	Oryza sativa subsp. japonica	Expressed protein	132	4.00	2.5	2
M7ZGK1	Triticum	Uncharacterized protein	133	4.00	8.0	2

	urartu					
W5GHT5	Triticum aestivum	Isocitrate dehydrogenase [NAD] subunit, mitochondrial	134	3.89	6.4	2
A0A1D6B171	Triticum aestivum	Uncharacterized protein	135	3.88	24.1	3
W5ES41	Triticum aestivum	Uncharacterized protein	136	3.74	31.8	17
W5AQ78	Triticum aestivum	Caleosin	137	3.62	6.0	2
F2DZ50	Hordeum vulgare subsp. vulgare	Predicted protein	138	3.61	6.1	2
R7W0Y8	Aegilops tauschii	Uncharacterized protein	139	3.60	4.1	2
A0A1D5RYQ7	Triticum aestivum	Uncharacterized protein	140	3.55	6.0	2
A0A1D5W9Y8	Triticum aestivum	Uncharacterized protein	141	3.50	14.9	3
M8ASR4	Aegilops tauschii	Peroxidase	142	3.49	9.2	2
A0A287EJZ6	Hordeum vulgare subsp. vulgare	Uncharacterized protein	143	3.42	1.2	1
W5AZ11	Triticum aestivum	Uncharacterized protein	144	3.40	32.9	5
BLAST search: M7YGB0 Ubiquitin-conjugating enzyme E2 2 (identity: 100%)						
Q6XEB8	Triticum aestivum	Malate dehydrogenase (Fragment)	145	3.26	3.5	1
E5KZQ6	Secale cereale	75k gamma secalin	146	3.24	42.1	27
Q9LKM0	Lolium perenne	Nucleoside diphosphate kinase	147	3.23	14.7	2
W5FAY5	Triticum aestivum	Actin	148	3.09	48.8	23
A0A1D5XQJ1	Triticum aestivum	Uncharacterized protein	149	3.02	12.8	4
W5H232	Triticum aestivum	Uncharacterized protein	150	3.01	5.7	2
W5B985	Triticum aestivum	Proliferating cell nuclear antigen	151	2.98	6.5	2

Q41584	Triticum aestivum	Thaumatin-like protein	152	2.91	15.3	2
A0A0C4BJJ3	Triticum aestivum	Uncharacterized protein	153	2.86	23.6	4
A0A287HID0	Hordeum vulgare subsp. vulgare	4-alpha-glucanotransferase	154	2.86	2.0	1
A0A1D6RF95	Triticum aestivum	Uncharacterized protein	155	2.85	7.8	2
M4GQ75	Bambusa emeiensis	Caffeoyl-CoA O-methyltransferase	156	2.85	10.1	2
W5I5D8	Triticum aestivum	Uncharacterized protein	157	2.82	8.9	1
F2DZF0	Hordeum vulgare subsp. vulgare	Mitochondrial fission 1 protein	158	2.82	12.4	2
W4ZR59	Triticum aestivum	Mitochondrial fission 1 protein	159	2.78	4.9	1
I1GLA9	Brachypodium distachyon	Uncharacterized protein	160	2.74	4.2	1
W5HPC1	Triticum aestivum	60S acidic ribosomal protein P0	161	2.70	6.3	1
F2EFA2	Hordeum vulgare subsp. vulgare	Predicted protein	162	2.70	7.7	2
W5ENP1	Triticum aestivum	Uncharacterized protein	163	2.66	6.9	1
Q41585	Triticum aestivum	Type V Thionin	164	2.63	21.5	3
M8BLL3	Aegilops tauschii	Pyruvate, phosphate dikinase 1, chloroplastic	165	2.57	0.6	1
M8A9J7	Triticum urartu	Catalase	166	2.57	1.6	1
A0A287NA39	Hordeum vulgare subsp. vulgare	Uncharacterized protein	167	2.56	11.6	5
BLAST search: P06293 Serpin-Z4 (identity: 99.0%)						
W5DWP8	Triticum aestivum	Uncharacterized protein	168	2.55	7.5	2
W5D2J1	Triticum aestivum	Importin subunit alpha	169	2.54	1.9	1

W5I3B1	Triticum aestivum	Uncharacterized protein	170	2.52	2.1	1
W5DBH3	Triticum aestivum	Aspartate aminotransferase	171	2.52	3.6	1
A0A1D5UZD7	Triticum aestivum	Uncharacterized protein	172	2.50	7.6	2
W5DR73	Triticum aestivum	Proteasome subunit alpha type	173	2.50	4.2	1
M8BHE6	Aegilops tauschii	26S protease regulatory subunit S10B	174	2.48	2.1	1
F8THZ7	Triticum aestivum	Protein disulfide-isomerase	175	2.42	22.1	12
W5E2X4	Triticum aestivum	Uncharacterized protein	176	2.41	5.4	1
W5HXF2	Triticum aestivum	Uncharacterized protein	177	2.41	3.5	1
R7W8L3	Aegilops tauschii	Uncharacterized protein	178	2.40	5.0	1
M8BX24	Aegilops tauschii	Uncharacterized protein	180	2.35	9.4	1
M8BZC4	Aegilops tauschii	Coatomer subunit alpha	181	2.33	1.6	2
W5AMP7	Triticum aestivum	Uncharacterized protein	182	2.33	2.8	1
W5GCA3	Triticum aestivum	Eukaryotic translation initiation factor 3 subunit F	183	2.31	3.1	1
D2KFH1	Triticum aestivum	Avenin-like a4	184	2.30	18.6	5
Q43660	Triticum aestivum	Heat shock protein 17.3	185	2.28	15.9	2
L7R664	Triticum aestivum	Gamma-gliadin	186	2.27	17.3	12
W5CRJ0	Triticum aestivum	Uncharacterized protein	187	2.25	1.6	1
A0A1E5VLY4	Dichanthelium oligosanthes	Protein IN2-1-like protein B	188	2.24	7.1	4
W5EF42	Triticum aestivum	Uncharacterized protein	189	2.23	3.2	1
W5C8D1	Triticum aestivum	Uncharacterized protein	190	2.22	14.7	2

A0A1D6D1Q3	Triticum aestivum	Pyrophosphate--fructose 6-phosphate 1-phosphotransferase subunit beta	191	2.20	2.1	1
R7W3W0	Aegilops tauschii	Cytochrome b-c1 complex subunit 7	192	2.20	7.1	1
M8BYD7	Aegilops tauschii	Purple acid phosphatase	193	2.19	2.2	1
M8CZB3	Aegilops tauschii	Tocopherol O-methyltransferase, chloroplastic	194	2.18	7.9	2
E2GJB9	Triticum aestivum	Profilin	195	2.17	24.4	2
W5D4G1	Triticum aestivum	Uncharacterized protein	196	2.17	6.9	2
C5X6V7	Sorghum bicolor	Uncharacterized protein	197	2.16	5.3	2
W5I956	Triticum aestivum	Uncharacterized protein	198	2.15	3.6	1
M7ZJD8	Triticum urartu	Palmitoyl-protein thioesterase 1	199	2.15	2.9	1
A0A0E0BBD6	Oryza glumipatula	Uncharacterized protein	200	2.14	1.0	1
W5C8P4	Triticum aestivum	Uncharacterized protein	201	2.14	4.8	2
W5FBQ2	Triticum aestivum	Uncharacterized protein	202	2.12	2.0	1
A0A1D5SR02	Triticum aestivum	Carboxypeptidase	203	2.12	2.4	1
F2ECH4	Hordeum vulgare subsp. vulgare	Predicted protein	204	2.12	3.6	1
W5B347	Triticum aestivum	Uncharacterized protein	205	2.07	3.1	1
R7WGB2	Aegilops tauschii	Signal recognition particle receptor subunit alpha	206	2.06	1.4	1
W5FIN3	Triticum aestivum	Uncharacterized protein	207	2.06	2.7	1
W5C4B9	Triticum aestivum	Uncharacterized protein	208	2.06	1.3	1
W5FB69	Triticum	Lysine--tRNA ligase	209	2.03	1.5	1

	aestivum					
W5CP75	Triticum aestivum	Vacuolar protein sorting-associated protein 28 homolog	211	2.03	6.1	1
M8C9S8	Aegilops tauschii	UPF0676 protein	212	2.03	4.7	1
W5FEU1	Triticum aestivum	Uncharacterized protein	213	2.01	5.9	1
F6LAX4	Triticum aestivum	Protein phosphatase 2A structural subunit	214	2.01	1.9	1
Q9ST58	Triticum aestivum	Serpin-Z1C	215	2	22.4	8
W5B757	Triticum aestivum	Uncharacterized protein	216	2	28.8	9
BLAST search: E0WC53 WALI7 (identity: 97.3%)						
SPZ2A	Triticum aestivum	Serpin-Z2A	217	2	13.8	6
W4ZSH7	Triticum aestivum	Uncharacterized protein	218	2	42.6	10
BLAST search: M7YRJ0 Ubiquitin-conjugating enzyme E2 7 (identity: 100%)						
A0A194YU99	Sorghum bicolor	Uncharacterized protein	219	2	5.3	4
B6T2A6	Zea mays	Stem-specific protein TSJT1	220	2	14.3	6
M8B621	Aegilops tauschii	Uncharacterized protein	221	2	20.6	4
C3VWV8	Secale cereale	Dimeric alpha-amylase inhibitor	222	2	40.9	6
W5DVU9	Triticum aestivum	Uncharacterized protein	223	2	3.7	2
V5RM09	Triticum aestivum	Puroindoline	224	2	15.0	3
M8CA11	Aegilops tauschii	DEAD-box ATP-dependent RNA helicase 40	225	2	2.7	2
P01083	Triticum aestivum	Alpha-amylase inhibitor 0.28	226	2	7.2	1
T1MDG3	Triticum urartu	Uncharacterized protein	227	2	10.3	2
I1H7J7	Brachypodium distachyon	Uncharacterized protein	228	2	5.0	1
M8BDJ4	Aegilops	Uncharacterized protein	229	2	2.9	1

	tauschii					
Q45FA6	Secale cereale	Cereal-type amylase inhibitor (Fragment)	230	2	7.6	1
W5I127	Triticum aestivum	Uncharacterized protein	231	2	9.8	1
W5D4W6	Triticum aestivum	Uncharacterized protein	232	2	2.0	1
A0A1D5WRV9	Triticum aestivum	Uncharacterized protein	233	2	9.3	1
W5FXK6	Triticum aestivum	Uncharacterized protein	234	2	1.6	1
A0A1D5RPN8	Triticum aestivum	Uncharacterized protein	235	2	7.4	1
X4YJY3	Triticum aestivum	Caleosin	236	2	5.6	1
W5HA38	Triticum aestivum	Uncharacterized protein	237	2	1.9	1
W5I868	Triticum aestivum	Uncharacterized protein	238	2	2.4	1
W5I6C5	Triticum aestivum	Uncharacterized protein	239	2	2.6	1
W5I2I7	Triticum aestivum	Uncharacterized protein	240	2	2.7	1
W5H1H4	Triticum aestivum	Uncharacterized protein	241	2	6.5	1
W5FNH7	Triticum aestivum	Uncharacterized protein	242	2	2.6	1
W5FI19	Triticum aestivum	Uncharacterized protein	243	2	2.2	1
W5F7H1	Triticum aestivum	Uncharacterized protein	244	2	2.1	1
W5D2A3	Triticum aestivum	Uncharacterized protein	245	2	8.1	1
W5BMW7	Triticum aestivum	Uncharacterized protein	246	2	5.3	1
W5AP46	Triticum aestivum	Uncharacterized protein	247	2	8.9	1
W5AKZ7	Triticum aestivum	Uncharacterized protein	248	2	1.9	1
W5A2C0	Triticum aestivum	Uncharacterized protein	249	2	4.7	1

W4ZU05	Triticum aestivum	Uncharacterized protein	250	2	3.5	1
W4ZPS8	Triticum aestivum	Uncharacterized protein	251	2	7.3	1
V5UPD1	Saccharum hybrid cultivar	B12D-like protein	252	2	10.3	1
T1N7I1	Triticum urartu	Uncharacterized protein	253	2	5.5	1
T1MVX3	Triticum urartu	Uncharacterized protein	254	2	4.6	1
R7W5Z8	Aegilops tauschii	Adenylate kinase A	255	2	2.9	1
Q9S8H2	Secale cereale	Major BAKER'S ASTHMA allergen SEC C 1 (Fragment)	256	2	42.3	1
Q9FXT2	Zea mays	Ethylene receptor	257	2	0.9	1
Q8VXC4	Oryza sativa	Glycine rich RNA binding protein	258	2	5.2	1
Q84VC8	Oryza sativa subsp. japonica	Gamma hydroxybutyrate dehydrogenase-like protein	259	2	5.5	1
A0A1D6CYN6	Triticum aestivum	Defective in cullin neddylation protein	260	2	3.2	1
Q5H9Y5	Oryza sativa subsp. japonica	p0650D04.17 protein	261	2	1.0	1
O82688	Hordeum vulgare	Amino acid selective channel protein	262	2	9.7	1
O24396	Triticum aestivum	Adenylosuccinate synthetase, chloroplastic (Fragment)	263	2	2.3	1
A0A1D6CKW3	Triticum aestivum	Uncharacterized protein	264	2	4.4	1
M8BVL4	Aegilops tauschii	Cytochrome b5	265	2	11.9	1
M8BUR6	Aegilops tauschii	Uncharacterized protein	266	2	4.4	1
M8BRA2	Aegilops tauschii	Lipase	267	2	2.1	1
M0UYA9	Hordeum vulgare subsp. vulgare	Uncharacterized protein	268	2	6.7	1

M0UGW6	Hordeum vulgare subsp. vulgare	Uncharacterized protein	269	2	6.5	1
K3ZUU8	Setaria italica	Uncharacterized protein	270	2	3.8	1
K3ZPT5	Setaria italica	Uncharacterized protein	271	2	10.5	1
K3Z3K1	Setaria italica	Uncharacterized protein	272	2	1.2	2
K3YK61	Setaria italica	Uncharacterized protein	273	2	6.7	1
I1PVE7	ORYGL	Kinesin-like protein	274	2	1.0	1
I1HLL5	Brachypodium distachyon	Uncharacterized protein	275	2	2.4	1
F2DJM5	Hordeum vulgare subsp. vulgare	Predicted protein	276	2	4.5	1
F2D9S4	Hordeum vulgare subsp. vulgare	Predicted protein	277	2	7.3	1
C5Z3P7	Sorghum bicolor	Uncharacterized protein	278	2	2.3	1
C5XJM0	Sorghum bicolor	Uncharacterized protein	279	2	2.8	1
B6TRI3	Zea mays	Leucine-rich repeat-containing protein 40	280	2	1.9	1
A0A1D6M5J0	Zea mays	Alpha/beta-Hydrolases superfamily protein	281	2	2.2	1
A0A1D6B277	Triticum aestivum	Uncharacterized protein	282	2	1.9	1
A0A1D5XTA3	Triticum aestivum	Uncharacterized protein	283	2	3.9	1
A0A0E0J5N3	Oryza nivara	ATP-dependent DNA helicase	284	2	1.4	1
A0A0E0CXH1	Oryza meridionalis	Uncharacterized protein	285	2	7.0	1
G1JSL4	Avena sativa	Peroxygenase 1	286	2	5.2	1
**Barley**						
**C-hordeins**						
Q84LE9	Hordeum vulgare	D-Hordein	2	17.38	40.7	32
Q5IUH1	Hordeum vulgare subsp. vulgare	Hordoindoline-B1	3	10.09	44.9	6

F2CR90	Hordeum vulgare subsp. vulgare	Predicted protein	4	8.14	39.4	7
BLAST search: Q41518 Single-stranded nucleic acid binding protein (identity: 90.4%)						
I6TEV2	Hordeum vulgare	Gamma 3 hordein	5	7.85	19.0	8
A0A287JQN1	Hordeum vulgare subsp. vulgare	Uncharacterized protein	6	7.47	33.8	4
BLAST search: M0UY52 Trypsin inhibitor CMe (identity: 37.5%)						
A0A287PVL8	Hordeum vulgare subsp. vulgare	Uncharacterized protein	7	6.86	22.1	4
BLAST search: P32936 Alpha-amylase/trypsin inhibitor CMb (identity: 100%)						
I6TMW4	Hordeum vulgare	B3 hordein	8	6.10	27.1	11
A0A060MZP1	Triticum aestivum	High molecular weight glutenin subunit (Fragment)	9	5.69	8.7	9
F2DZW3	Hordeum vulgare subsp. vulgare	Predicted protein	10	5.66	14.8	3
W6AX70	Triticum aestivum	High molecular weight glutenin subunit	11	5.08	8.9	7
Q5URW7	Hordeum vulgare subsp. vulgare	Hordoindoline b-2	13	4.24	38.1	5
F2CXF8	Hordeum vulgare subsp. vulgare	Predicted protein	14	4.22	22.4	2
BLAST search: P43472 Glycine-rich RNA-binding protein blt801 (identity: 97.5%)						
M0V3U0	Hordeum vulgare subsp. vulgare	Non-specific lipid-transfer protein	16	4.17	37.6	4
F2EJS0	Hordeum vulgare subsp. vulgare	Predicted protein (Fragment)	17	4.06	22.3	4
BLAST search: P21742 Beta-Hordothionin (identity: 98.8%)						
P80284	Hordeum vulgare	Protein disulfide isomerase	18	3.53	4.1	2
Q2V8X0	Hordeum	Limit dextrinase inhibitor	19	3.49	22.5	2

	vulgare					
M0W9B7	Hordeum vulgare subsp. vulgare	Uncharacterized protein	20	3.47	55.4	3
BLAST search: P16062 Subtilisin-chymotrypsin inhibitor CI-1A (identity: 100%)						
P28041	Hordeum vulgare	Alpha-amylase/trypsin inhibitor CMa	22	3.11	30.3	6
Q40055	Hordeum vulgare	C hordein	23	2.88	8.9	7
P06470	Hordeum vulgare	B1-hordein	24	2.15	22.5	10
P11643	Hordeum vulgare	Alpha-amylase/trypsin inhibitor CMd	25	2.09	9.9	1
M0UFI7	Hordeum vulgare subsp. vulgare	Uncharacterized protein	26	2.04	11.5	1
BLAST search: P34937 Triosephosphate isomerase, cytosolic (identity: 99.1%)						
P01086	Hordeum vulgare	Trypsin inhibitor CMe	27	2.02	8.8	1
W4ZMU6	Triticum aestivum	Uncharacterized protein	28	2	3.6	1
Q96458	Hordeum vulgare	17 kDa class I small heat shock protein	29	2	10.0	1
N1QWT9	Aegilops tauschii	Hydroxymethylglutaryl-CoA synthase	30	2	2.2	1
I3NM41	Triticum aestivum	Oleosin	31	2	10.4	1
F2E8C1	Hordeum vulgare subsp. vulgare	Predicted protein	32	2	9.1	1
B6SV48	Zea mays	4-coumarate--CoA ligase	33	2	2.1	1
A0A1Z5RPZ1	Sorghum bicolor	Uncharacterized protein	34	2	5.7	1
A0A0D9WKR1	Leersia perrieri	Uncharacterized protein	35	2	7.2	2
**γ-hordeins**						
I6TMW4	Hordeum vulgare	B3 hordein	2	33.9	51.3	60
P06470	Hordeum vulgare	B1-hordein	3	16.3	45.1	64

P80198	Hordeum vulgare	Gamma-hordein-3	4	13.2	26.6	16
Q4G3S6	Hordeum chilense	B3-hordein	5	8.0	15.7	16
P34951	Hordeum vulgare	Trypsin inhibitor CMc	7	5.2	31.5	6
P01086	Hordeum vulgare	Trypsin inhibitor CMe	8	4.2	29.1	3
P11643	Hordeum vulgare	Alpha-amylase/trypsin inhibitor CMd	9	4.0	18.7	2
AVLA4	Triticum aestivum	Avenin-like a4	10	4.0	16.3	2
Q84LE9	Hordeum vulgare	D-Hordein	12	3.1	6.2	4
Q2XQF1	Hordeum vulgare subsp. vulgare	B hordein	13	2.4	48.7	52
B9VRI3	Triticum macha	Alpha-amylase inhibitor CM16 subunit (Fragment)	14	2.2	11.3	1
I6R4A7	Hordeum vulgare subsp. vulgare	B-hordein	15	2	30.3	37
Q6PKM2	Elymus elongatus	Low molecular weight glutenin subunit	16	2	10.2	10
P16968	Hordeum vulgare subsp. vulgare	Alpha-amylase inhibitor BMAI-1 (Fragment)	17	2	9.6	1
F2E6J8	Hordeum vulgare subsp. vulgare	Predicted protein (Fragment)	18	2	4.5	1
J3MJG3	Oryza brachyantha	Uncharacterized protein	19	2	1.8	1
A0A0D9W175	Leersia perrieri	Phospholipase D	20	2	2.6	1
A0A0B4SVD8	Triticum aestivum	Receptor kinase-like protein	21	2	2.8	1
A0A0A9MI62	Arundo donax	Uncharacterized protein	22	2	21.7	1
A0A0A8Y0G5	Arundo donax	Uncharacterized protein	23	2	15.9	1
**B-hordeins**						
F2D284	Hordeum	Protein disulfide-isomerase	1	31.05	43.5	22

	vulgare subsp. vulgare					
F2E9N0	Hordeum vulgare subsp. vulgare	Predicted protein	2	22.68	28.4	17
BLAST search: M7ZK46 12S seed storage globulin 1 (identity: 86.6%)						
I6TMW4	Hordeum vulgare	B3 hordein	3	21.90	49.1	102
P16098	Hordeum vulgare	Beta-amylase	4	19.68	30.8	17
I6SW23	Hordeum vulgare	D hordein	5	17.52	43.5	42
M0Z0D3	Hordeum vulgare subsp. vulgare	Malate dehydrogenase	6	15.75	30.4	12
P08477	Hordeum vulgare	Glyceraldehyde-3-phosphate dehydrogenase 2, cytosolic (Fragment)	8	10.71	28.9	7
Q6E5A5	Hordeum vulgare subsp. vulgare	Plastidial ADP-glucose transporter	9	10.35	15.1	6
Q9T2L4	Hordeum vulgare	26 kDa heat shock protein	10	8.48	21.3	5
M0WX05	Hordeum vulgare subsp. vulgare	Uncharacterized protein	11	8.12	9.8	4
BLAST search: M7YWL9 Selenium-binding protein (identity: 99.0%)						
Q945R5	Hordeum vulgare	Ascorbate peroxidase	12	8.11	28.5	6
P52572	Hordeum vulgare	1-Cys peroxiredoxin PER1	13	8.03	20.6	4
F2E8C1	Hordeum vulgare subsp. vulgare	Predicted protein	14	8.00	38.0	4
A0A287SRS7	Hordeum vulgare subsp. vulgare	rRNA N-glycosidase	15	7.77	19.6	5
A0A287NA39	Hordeum vulgare subsp. vulgare	Uncharacterized protein	16	7.22	16.8	6
BLAST search: P06293 Serpin-Z4 (identity: 98.2%)						
I6TEV2	Hordeum	Gamma 3 hordein	17	6.98	12.5	13

	vulgare					
A0A287TCL5	Hordeum vulgare subsp. vulgare	Peptidyl-prolyl cis-trans isomerase	18	6.84	38.6	10
P01086	Hordeum vulgare.	Trypsin inhibitor CMe	19	6.58	33.8	6
A0A287P659	Hordeum vulgare subsp. vulgare	Uncharacterized protein	20	6.46	27.9	6
A0A287K1P5	Hordeum vulgare subsp. vulgare	Uncharacterized protein	21	6.40	19.9	3
M8BDX3	Aegilops tauschii	Uncharacterized protein	22	6.28	13.2	4
P52894	Hordeum vulgare	Alanine aminotransferase 2	23	5.93	7.1	3
P13691	Hordeum vulgare	Alpha-amylase inhibitor BDAI-1	24	5.90	38.2	6
P06470	Hordeum vulgare	B1-hordein	25	5.64	45.7	122
A0A287HKD4	Hordeum vulgare subsp. vulgare	Sucrose synthase	26	5.30	6.1	4
F2D6B1	Hordeum vulgare subsp. vulgare	Predicted protein	27	5.28	18.4	3
F2EEV5	Hordeum vulgare subsp. vulgare	Peroxidase	29	5.10	10.6	4
A0A287N132	Hordeum vulgare subsp. vulgare	Uncharacterized protein	30	4.86	17.8	3
BLAST search: MP45851Oxalate oxidase 2 (identity: 83.6%)						
P16968	Hordeum vulgare	Alpha-amylase inhibitor BMAI-1 (Fragment)	31	4.59	26.7	3
F2CUW7	Hordeum vulgare subsp. vulgare	Predicted protein	32	4.56	18.6	2
A0A287V373	Hordeum vulgare subsp. vulgare	Uncharacterized protein	33	4.55	6.5	4

F2DTH9	Hordeum vulgare subsp. vulgare	Predicted protein	34	4.51	6.7	2
A0A287EFG2	Hordeum vulgare subsp. vulgare	Uncharacterized protein	35	4.35	45.0	57
BLAST search: P06470 B1-hordein (identity: 94.6%)						
M0V3U0	Hordeum vulgare subsp. vulgare	Non-specific lipid-transfer protein	36	4.32	46.1	6
Q40052	Hordeum vulgare	Glycine rich protein, RNA binding protein	37	4.30	37.0	4
W5CWR9	Triticum aestivum	Uncharacterized protein	38	4.28	6.5	2
Q40026	Hordeum vulgare	B hordein	39	4.19	39.0	56
F2DD34	Hordeum vulgare subsp. vulgare	Predicted protein	40	4.12	24.2	3
M8CGD2	Aegilops tauschii	Elongation factor 2	41	4.06	5.2	2
M0USC9	Hordeum vulgare subsp. vulgare	Aspartate aminotransferase	42	4.05	4.4	2
W5E2W7	Triticum aestivum	40S ribosomal protein SA	43	4.05	6.2	2
W5CAC3	Triticum aestivum	Uncharacterized protein	44	4.04	12.4	2
BLAST search: Q9FZ48 Ubiquitin-conjugating enzyme E2 36 (identity: 99.3%)						
M0V6E2	Hordeum vulgare subsp. vulgare	Uncharacterized protein	46	4.02	7.1	2
M0X060	Hordeum vulgare subsp. vulgare	Uncharacterized protein	47	4.00	18.8	2
P11643	Hordeum vulgare	Alpha-amylase/trypsin inhibitor CMd	48	4.00	30.4	4
J9V7J5	Hordeum vulgare subsp. vulgare	Isa (Fragment)	49	4.00	15.3	2
F2CXF8	Hordeum vulgare subsp.	Predicted protein	50	4.00	15.5	2

	vulgare					
Q4L1B2	Hordeum vulgare	Glucose-1-phosphate adenylyltransferase	51	3.82	5.3	2
F2CYL7	Hordeum vulgare subsp. vulgare	Predicted protein	52	3.72	5.2	2
BLAST search: Q43472 Glycine-rich RNA-binding protein blt801 (identity: 97.5%)						
D2KFH1	Triticum aestivum	Avenin-like a4	53	3.71	14.0	4
F2CXK6	Hordeum vulgare subsp. vulgare	Predicted protein	55	3.41	7.8	2
A0A287WCP3	Hordeum vulgare subsp. vulgare	Uncharacterized protein	56	3.31	32.6	5
BLAST search: A0S6X4 FT-like protein (identity: 50.9%)						
M0XQ24	Hordeum vulgare subsp. vulgare	Uncharacterized protein	57	3.28	1.7	1
P32936	Hordeum vulgare	Alpha-amylase/trypsin inhibitor CMb	58	3.12	16.8	2
W5I774	Triticum aestivum	Sucrose synthase	59	2.94	1.1	1
P34951	Hordeum vulgare	Trypsin inhibitor CMc	60	2.85	27.3	4
P01545	Hordeum vulgare	Alpha-hordothionin	61	2.81	15.0	2
U6A1T2	Hordeum vulgare	Glucose-1-phosphate adenylyltransferase	62	2.77	4.0	2
V9SHG4	Hordeum vulgare subsp. vulgare	Starch synthase, chloroplastic/amyloplastic	63	2.72	3.5	2
Q5URW6	Hordeum vulgare subsp. vulgare	Hordoindoline b-1	64	2.63	9.5	1
P28041	Hordeum vulgare	Alpha-amylase/trypsin inhibitor CMa	65	2.58	30.3	4
F2CXT7	Hordeum vulgare	Fructose-bisphosphate aldolase	66	2.46	5.9	2
Q40025	Hordeum vulgare	Beta-glucosidase	67	2.42	1.8	1

Q7XYE2	Triticum aestivum	Catalase isozyme (Fragment)	68	2.40	3.0	1
F2EH52	Hordeum vulgare subsp. vulgare	Predicted protein	70	2.36	24.1	3
F2D1G5	Hordeum vulgare subsp. vulgare	Predicted protein	71	2.35	4.4	2
Q8H1L9	Hordeum vulgare	Actin	72	2.28	2.7	1
F2DCE3	Hordeum vulgare subsp. vulgare	Pyruvate, phosphate dikinase	73	2.22	1.6	1
A0A287M898	Hordeum vulgare subsp. vulgare	Uncharacterized protein	74	2.19	6.7	2
BLAST search: A0A3B6TUD9 Thiamine thiazole synthase (identity: 78.1%)						
T1LDI8	Triticum urartu	Uncharacterized protein	75	2.14	1.7	1
Q7XZK6	Hordeum vulgare	Starch branching enzyme I (Fragment)	76	2.13	1.3	1
F2DZW3	Hordeum vulgare subsp. vulgare	Predicted protein	77	2.08	4.8	1
F2ECH4	Hordeum vulgare subsp. vulgare	Predicted protein	78	2.08	4.1	1
W5EH96	Triticum aestivum	Uncharacterized protein	79	2.07	14.7	2
BLAST search: M7Z528 14kDa zinc-binding protein (identity: 99.2%)						
F2DCS7	Hordeum vulgare subsp. vulgare	Ketol-acid reductoisomerase	80	2.07	4.3	2
A0A287RUV4	Hordeum vulgare subsp. vulgare	Uncharacterized protein	81	2.05	5.2	1
A0A287WXA5	Hordeum vulgare subsp. vulgare	Glyceraldehyde-3-phosphate dehydrogenase	82	2.04	12.4	3
Q9FSI8	Hordeum vulgare	Cold-regulated protein	83	2.03	6.1	1
F2D4L0	Hordeum	Predicted protein	84	2.03	11.7	2

	vulgare subsp. vulgare					
C5XN41	Sorghum bicolor	Uncharacterized protein	85	2.03	8.7	1
A8V498	Hordeum vulgare subsp. vulgare	Chymotrypsin inhibitor-2	86	2.03	14.3	1
W5EMF3	Triticum aestivum	Ubiquitin-fold modifier-conjugating enzyme 1	87	2.02	5.4	1
W5CPG2	Triticum aestivum	Uncharacterized protein	88	2.02	1.0	1
W5I4U0	Triticum aestivum	Uncharacterized protein	89	2.01	8.1	1
W5B1E5	Triticum aestivum	Superoxide dismutase [Cu-Zn]	90	2.01	8.6	1
F2DGA8	Hordeum vulgare subsp. vulgare	Predicted protein	91	2	28.5	3
P42210	Hordeum vulgare	Phytepsin	92	2	4.5	3
A0A0D9X308	Leersia perrieri	Uncharacterized protein	93	2	7.4	2
Q40055	Hordeum vulgare	C hordein	94	2	2.6	2
M0VYC1	Hordeum vulgare subsp. vulgare	Uncharacterized protein	95	2	2.5	1
W5FPN2	Triticum aestivum	Uncharacterized protein	96	2	2.0	1
A0A287WJQ6	Hordeum vulgare subsp. vulgare	Uncharacterized protein	97	2	6.2	1
Q2V8X0	Hordeum vulgare	Limit dextrinase inhibitor	98	2	6.8	1
F2CV01	Hordeum vulgare subsp. vulgare	Predicted protein	99	2	6.2	1
J3ML64	Oryza brachyantha	Uncharacterized protein	100	2	1.2	1
M0YMY2	Hordeum vulgare subsp.	UDP-glucose 6-dehydrogenase	101	2	1.8	1

	vulgare					
A0A287QLB7	Hordeum vulgare subsp. vulgare	Uncharacterized protein	102	2	4.8	1
W5FZ62	Triticum aestivum	Uncharacterized protein	103	2	2.5	1
W5FZ59	Triticum aestivum	Uncharacterized protein	104	2	7.2	1
W5FS88	Triticum aestivum	Uncharacterized protein	105	2	4.2	1
W5FJT8	Triticum aestivum	Tubulin beta chain	106	2	4.0	1
W5E416	Triticum aestivum	Uncharacterized protein	107	2	2.4	1
W5D322	Triticum aestivum	Uncharacterized protein	108	2	1.6	1
W5BV35	Triticum aestivum	Uncharacterized protein	109	2	7.9	1
W5AR77	Triticum aestivum	Uncharacterized protein	110	2	5.6	1
W5AQ87	Triticum aestivum	Uncharacterized protein	111	2	10.1	1
M0X743	Hordeum vulgare subsp. vulgare	Cytochrome b-c1 complex subunit 7	112	2	7.1	1
M0YB08	Hordeum vulgare subsp. vulgare	4-hydroxy-4-methyl-2-oxoglutarate aldolase	113	2	5.4	1
F2CXF2	Hordeum vulgare subsp. vulgare	Predicted protein	114	2	3.1	1
M0YZF1	Hordeum vulgare subsp. vulgare	Ubiquitin-fold modifier 1	115	2	1.4	1
F2EG29	Hordeum vulgare subsp. vulgare	Predicted protein	116	2	9.0	1
M0Y7M3	Hordeum vulgare subsp. vulgare	Uncharacterized protein	117	2	3.3	1
F2DEF0	Hordeum vulgare subsp.	Predicted protein	118	2	1.0	1

	vulgare					
F2EL44	Hordeum vulgare subsp. vulgare	Predicted protein	119	2	1.6	1
M0XBS5	Hordeum vulgare subsp. vulgare	Uncharacterized protein	120	2	18.1	1
M0VT27	Hordeum vulgare subsp. vulgare	Uncharacterized protein	121	2	14.3	1
F2EJW3	Hordeum vulgare subsp. vulgare	Predicted protein	122	2	14.5	1
F2E8B4	Hordeum vulgare subsp. vulgare	Predicted protein	123	2	3.0	1
F2E6K4	Hordeum vulgare subsp. vulgare	Predicted protein	124	2	6.1	1
F2DA79	Hordeum vulgare subsp. vulgare	Predicted protein	125	2	2.4	1
F2D8P4	Hordeum vulgare subsp. vulgare	Predicted protein	126	2	3.9	1
F2CQP8	Hordeum vulgare subsp. vulgare	Predicted protein	127	2	3.8	1
B8BJI1	Oryza sativa subsp. indica	Uncharacterized protein	128	2	0.7	1
B8AYX1	Oryza sativa subsp. indica	Uncharacterized protein	129	2	5.8	1
A0A287K2A4	Hordeum vulgare subsp. vulgare	Uncharacterized protein	130	2	2.2	1
A0A1D5VUQ0	Triticum aestivum	Uncharacterized protein	131	2	1.5	1
**D-hordeins**						
I6TRS8	Hordeum vulgare	D hordein	1	35.20	54.6	209
F2DZW3	Hordeum	Predicted protein	3	13.57	34.0	8

	vulgare subsp. vulgare					
BLAST search: Q41350 Osmotin-like protein (identity: 50.0%)						
F2CR90	Hordeum vulgare subsp. vulgare	Predicted protein	4	11.63	58.9	10
BLAST search: Q41518 Single-stranded nucleic acid binding protein (identity: 90.4%)						
P28041	Hordeum vulgare	Alpha-amylase/trypsin inhibitor CMa	5	11.35	55.9	16
P01545	Hordeum vulgare	Alpha-hordothionin	6	10.32	36.2	10
P06470	Hordeum vulgare	B1-hordein	7	10.25	20.8	8
Q03678	Hordeum vulgare	Embryo globulin	8	9.58	8.6	7
U6A1T2	Hordeum vulgare	Glucose-1-phosphate adenylyltransferase	9	9.28	13.2	8
F2EJF0	Hordeum vulgare subsp. vulgare	Predicted protein	10	9.07	28.1	11
BLAST search: Q0Q5D4 Globulin 1 (identity: 83.0%)						
F2CXF8	Hordeum vulgare subsp. vulgare	Predicted protein	11	8.86	44.7	5
BLAST search: Q43472 Glycine-rich RNA-binding protein blt801 (identity: 97.5%)						
Q5IU17	Hordeum vulgare subsp. vulgare	Hordoindoline-B2	12	8.35	33.3	5
F2EC88	Hordeum vulgare subsp. vulgare	Predicted protein	13	8.25	27.0	7
F2DD34	Hordeum vulgare subsp. vulgare	Predicted protein	14	8.20	40.3	8
F2ECH4	Hordeum vulgare subsp. vulgare	Predicted protein	15	7.45	14.1	4
F2D224	Hordeum vulgare subsp. vulgare	Predicted protein	16	7.18	12.7	3
A0A287TMY0	Hordeum vulgare subsp.	Uncharacterized protein	17	7.13	48.9	4

	vulgare					
BLAST search: P0CG83 Polyubiquitin (identity: 99.3%)						
F2E9N0	Hordeum vulgare subsp. vulgare	Predicted protein	18	7.02	9.6	4
BLAST search: M7ZK46 12S seed storage globulin (identity: 86.6%)						
M0Z0D3	Hordeum vulgare subsp. vulgare	Malate dehydrogenase	19	6.87	14.2	5
A0A287SV63	Hordeum vulgare subsp. vulgare	Uncharacterized protein	20	6.78	9.0	5
BLAST search: M7ZB42 Calreticulin (identity: 78.5%)						
F2DJM5	Hordeum vulgare subsp. vulgare	Predicted protein	21	6.63	17.8	4
F2CW55	Hordeum vulgare subsp. vulgare	Small ubiquitin-related modifier	22	6.60	40.6	4
F2D284	Hordeum vulgare subsp. vulgare	Protein disulfide-isomerase	23	6.57	6.2	3
F2DHH7	Hordeum vulgare subsp. vulgare	Superoxide dismutase [Cu-Zn]	24	6.40	43.4	6
Q6LAA4	Hordeum vulgare	Elongation factor 1-alpha	25	6.24	10.3	4
A0A060MZP1	Triticum aestivum	High molecular weight glutenin subunit (Fragment)	26	6.07	6.8	20
Q8H1L9	Hordeum vulgare	Actin	27	6.05	12.7	4
Q5UNP2	Hordeum vulgare subsp. vulgare	Non-specific lipid-transfer protein	28	6.00	20.2	3
F2EB17	Hordeum vulgare	Predicted protein	29	6.00	5.0	3
F2EL01	Hordeum vulgare subsp. vulgare	Predicted protein	30	5.74	18.8	3
M0WF40	Hordeum vulgare subsp.	Uncharacterized protein	31	5.37	7.0	3

	vulgare					
BLAST search: M8A775 Elongation factor 1-gamma 2 (identity: 97.3%)						
Q9SAU8	Triticum aestivum	HSP70	32	5.30	5.6	4
M0V3U0	Hordeum vulgare subsp. vulgare	Non-specific lipid-transfer protein	33	5.10	52.1	15
M0WX05	Hordeum vulgare subsp. vulgare	Uncharacterized protein	34	4.84	9.4	4
BLAST search: M7YWL9 Selenium-binding protein (identity: 98.2%)						
I6TEV2	Hordeum vulgare	Gamma 3 hordein	35	4.61	15.9	4
Q7FPQ5	Hordeum vulgare	Ascorbate peroxidase (Fragment)	36	4.34	22.2	3
F2D326	Hordeum vulgare subsp. vulgare	Predicted protein	37	4.22	25.0	3
BLAST search: P14928 ABA-inducible protein PHV A1 (identity: 99.2%)						
C3W8M1	Hordeum vulgare subsp. vulgare	Starch branching enzyme (Fragment)	39	4.16	4.5	2
M0XJ70	Hordeum vulgare	Uncharacterized protein	40	4.11	21.6	2
P01086	Hordeum vulgare	Trypsin inhibitor CMe	41	4.09	29.1	3
Q2V8X0	Hordeum vulgare	Limit dextrinase inhibitor	42	4.02	36.1	5
Q93W25	Triticum aestivum	Peptidyl-prolyl cis-trans isomerase	43	4.00	17.0	2
Q05191	Hordeum vulgare	Late embryogenesis abundant protein B19.4	44	4.00	16.3	2
F2CS22	Hordeum vulgare	Predicted protein	45	4.00	8.5	2
F2CR08	Hordeum vulgare	Predicted protein	46	3.78	5.6	3
M0Y075	Hordeum vulgare subsp. vulgare	Bowman-Birk type trypsin inhibitor	47	3.72	12.3	3
P06471	Hordeum vulgare	B3-hordein (Fragment)	48	3.62	20.8	7

W6AX70	Triticum aestivum	High molecular weight glutenin subunit	49	3.56	7.1	9
F2D6Y8	Hordeum vulgare subsp. vulgare	Peptide-methionine (R)-S-oxide reductase	50	3.48	25.3	3
A0A287JQN1	Hordeum vulgare subsp. vulgare	Uncharacterized protein	51	3.37	17.5	4
BLAST search: Q7X7E6 17kDa alpha-amylase/trypsin inhibitor 1 (identity: 50.0%)						
F2CTJ8	Hordeum vulgare subsp. vulgare	Predicted protein	53	3.29	3.6	2
F2DY31	Hordeum vulgare subsp. vulgare	Predicted protein	54	3.26	17.4	2
P32936	Hordeum vulgare	Alpha-amylase/trypsin inhibitor CMb	55	3.15	13.4	2
A5CFY4	Hordeum vulgare subsp. vulgare	Tubulin beta chain	56	3.04	6.0	2
Q5DVL6	Hordeum vulgare subsp. vulgare	C2 domain-containing protein	57	2.99	7.7	2
F2DIF8	Hordeum vulgare subsp. vulgare	Pyrophosphate--fructose 6-phosphate 1-phosphotransferase subunit alpha	58	2.86	4.2	3
A0A287Q3Y5	Hordeum vulgare subsp. vulgare	Uncharacterized protein	59	2.77	17.3	2
A5CFY6	Hordeum vulgare subsp. vulgare	Tubulin beta chain	60	2.70	5.8	2
F2D6N2	Hordeum vulgare subsp. vulgare	Uncharacterized protein	61	2.67	2.9	2
BLAST search: M7ZQL9 Heat shock 70kDa protein 4L (identity: 94.6%)						
F2CX17	Hordeum vulgare subsp. vulgare	Predicted protein	62	2.66	44.8	7
BLAST search: A0A3B5YZ25 cold shock domain protein 2 (identity: 88.3%)						

Q40058	Hordeum vulgare	HSP70	63	2.64	4.1	3
F2EI14	Hordeum vulgare subsp. vulgare	Uncharacterized protein	64	2.58	15.4	2
BLAST search: Q94KM0 HSP17 (identity: 90.1%)						
A0A287KNT7	Hordeum vulgare subsp. vulgare	Uncharacterized protein	65	2.54	19.8	2
Q4L1B2	Hordeum vulgare	Glucose-1-phosphate adenylyltransferase	66	2.53	5.5	2
F2CPQ3	Hordeum vulgare subsp. vulgare	Predicted protein	67	2.30	7.9	1
E5AXU6	Hordeum vulgare	Vacuolar-processing enzyme 2c	68	2.27	2.0	1
Q94B09	Hordeum vulgare	Calcium-binding protein 1	69	2.26	8.2	1
THNB	Hordeum vulgare	Beta-hordothionin	70	2.23	27.9	8
Q6E5A5	Hordeum vulgare subsp. vulgare	Plastidial ADP-glucose transporter	71	2.23	2.1	1
M0WPC3	Hordeum vulgare subsp. vulgare	Probable non-specific lipid-transfer protein	72	2.22	27.0	3
M0UFI7	Hordeum vulgare subsp. vulgare	Uncharacterized protein	73	2.16	23.9	2
BLAST search: P34937 Triosephosphate isomerase (identity: 99.1%)						
Q42849	Hordeum vulgare	Non-specific lipid-transfer protein	74	2.16	18.3	2
G4Y3Y0	Triticum aestivum	High-molecular-weight glutenin subunit Bx7.1	75	2.14	4.0	19
F2D4Y3	Hordeum vulgare subsp. vulgare	Predicted protein (Fragment)	76	2.10	12.0	1
F2D0G5	Hordeum vulgare subsp. vulgare	Cytochrome c oxidase subunit	77	2.08	10.4	1
M0UTD1	Hordeum vulgare subsp.	Fructose-bisphosphate aldolase	78	2.07	3.9	1

	vulgare					
F2DF10	Hordeum vulgare subsp. vulgare	Predicted protein	79	2.04	1.6	1
Q43769	Hordeum vulgare	Oleosin	80	2.03	6.0	1
P07596	Hordeum vulgare	Alpha-amylase/subtilisin inhibitor	81	2.03	6.9	2
H6UQP6	Elymus libanoticus	High molecular weight subunit of glutenin	84	2	5.5	21
A0A287X6Z0	Hordeum vulgare subsp. vulgare	Uncharacterized protein	85	2	7.0	3
BLAST search: M8A775 Elongation factor 1-gamma 2 (identity: 96.9%)						
M0WBH4	Hordeum vulgare subsp. vulgare	Uncharacterized protein	86	2	8.2	1
Q39999	Hordeum vulgare	Gamma-thionin	87	2	26.8	3
F2CS23	Hordeum vulgare subsp. vulgare	40S ribosomal protein S21	88	2	13.4	1
F2DVM8	Hordeum vulgare subsp. vulgare	Predicted protein	89	2	4.9	2
F2EH52	Hordeum vulgare subsp. vulgare	Predicted protein	90	2	9.5	1
M0XHQ5	Hordeum vulgare subsp. vulgare	Uncharacterized protein	91	2	14.9	1
A0A287THI2	Hordeum vulgare subsp. vulgare	Uncharacterized protein	92	2	9.9	1
W5FWF5	Triticum aestivum	Uncharacterized protein	93	2	5.8	1
Q9T2L5	Hordeum vulgare	26 kDa heat shock protein	94	2	4.6	1
F2E325	Hordeum vulgare subsp. vulgare	Predicted protein	95	2	10.0	1

F2DNB2	Hordeum vulgare subsp. vulgare	Mitochondrial fission 1 protein	96	2	6.7	1
T1M2Y3	Triticum urartu	Uncharacterized protein	97	2	6.3	1
P11643	Hordeum vulgare	Alpha-amylase/trypsin inhibitor CMd	98	2	9.9	1
F2DB07	Hordeum vulgare subsp. vulgare	Uncharacterized protein	99	2	9.0	1
A0A287XGW7	Hordeum vulgare subsp. vulgare	Uncharacterized protein	100	2	6.5	1
A0A287V267	Hordeum vulgare subsp. vulgare	Uncharacterized protein	101	2	4.0	1
A0A287RSZ8	Hordeum vulgare subsp. vulgare	Uncharacterized protein	102	2	5.6	1
A0A287LNA3	Hordeum vulgare subsp. vulgare	Uncharacterized protein	103	2	12.0	1
A0A0A8XXT2	Arundo donax	Uncharacterized protein	104	2	14.7	1

[a] missing rank numbers corresponded to contaminants

[b] A measure of the protein confidence for a detected protein, calculated from the peptide confidence for peptides from spectra that are not already completely “used” by higher scoring winning proteins

1.4 Supplementary Table S4

Identified proteins (1% global FDR) in each wheat, rye and barley gluten protein type after chymotryptic hydrolysis with their UniProtKB accession number, species, name, rank, score, coverage and number of identified peptides. The rank of a specific protein is given relative to all other detected proteins.

Uniprot accession	Species	Name	Rank[a]	Score[b]	Cove-rage	Pep-tides
**Wheat**						
**α-gliadins**						
J7I026	Triticum aestivum	Alpha-gliadin	1	19.29	46.4	34
A0A0U2P410	Aegilops longissima	Low molecular weight glutenin subunit	2	14.37	28.0	19
I3XHQ1	Triticum aestivum	Low molecular weight glutenin subunit LMW-9	3	8.51	22.8	7
R4JBJ8	Triticum aestivum	Low-molecular-weight glutenin subunit (Fragment)	4	7.73	21.9	7
A0A2D2CI59	Triticum spelta	HMW glutenin y-type subunit By22.1	5	7.56	17.6	7
W0C8N8	Triticum aestivum	High-molecular-weight glutenin subunit 1Bx14	6	6.77	10.0	5
Q03872	Triticum aestivum	High molecular weight glutenin subunit 1Ax1	7	6.52	8.7	5
I0IT51	Triticum aestivum	Alpha/beta-gliadin	8	6.18	26.9	9
A7X9X7	Triticum turgidum	Low molecular weight glutenin (Fragment)	9	6.00	17.0	5
A0A0S2GJT4	Triticum aestivum	Low-molecular-weight glutenin subunit	10	5.70	19.7	7
L7VFZ3	Aegilops tauschii	Delta gliadin 1	11	4.93	10.5	3
**γ-gliadins**						
Q9XGF0	Triticum turgidum subsp. durum	Low molecular weight glutenin subunit (Fragment)	2	4.97	9.5	4
B6UKM7	Triticum aestivum	Gamma-gliadin	3	2.97	3.6	1
P94021	Triticum aestivum	LMM glutenin 2 (Fragment)	4	2.92	3.5	1

B6UKM4	Triticum aestivum	Gamma-gliadin	5	2.83	22.5	7
Q5XZE4	Triticum polonicum	High molecular weight glutenin subunit Bx7.1 (Fragment)	6	2.42	4.4	3
R4JDK8	Triticum aestivum	Low-molecular-weight glutenin subunit	7	2.36	4.6	1
T2HRF3	Triticum aestivum	High-molecular-weight glutenin subunit	8	2	1.6	1
W5FZ62	Triticum aestivum	Uncharacterized protein	9	2	3.0	1
BLAST search: P93693 Serpin-Z1B (identity: 99.5%)						
A0A0A9QR17	Arundo donax	Uncharacterized protein	10	2	92.9	1
**ω5-gliadins**						
D6RVY4	Triticum aestivum	Low molecular glutenin subunit (Fragment)	1	6.66	14.0	10
P10387	Triticum aestivum	Glutenin, high molecular weight subunit Dy10	2	6.45	20.8	9
Q41553	Triticum aestivum	HMW glutenin subunit Ax2	4	2.49	5.6	4
**ω1,2-gliadins**						
A0A060N0S6	Triticum aestivum	Omega-gliadin (Fragment)	1	17.11	73.5	89
P10388	Triticum aestivum	Glutenin, high molecular weight subunit DX5	2	4.84	9.2	6
T1LG74	Triticum urartu	Uncharacterized protein	3	4.53	8.2	3
BLAST search: P10385 Glutenin, low molecular weight subunit (identity: 83.6%)						
A0A060MZP1	Triticum aestivum	High molecular weight glutenin subunit (Fragment)	5	4.08	9.1	5
P10387	Triticum aestivum	Glutenin, high molecular weight subunit DY10	6	3.91	18.5	10
Q571R2	Triticum aestivum	Putative omega-gliadin (Fragment)	7	2.85	24.0	12
D6QY47	Triticum monococcum	Omega-gliadin (Fragment)	8	2.23	37.7	27
**HMW-GS**						
C0SUC3	Triticum aestivum	High-molecular-weight glutenin subunit x5	1	27.39	35.6	39
P10387	Triticum	Glutenin, high molecular	2	13.58	36.4	44

	aestivum	weight subunit DY10				
Q03872	Triticum aestivum	High molecular weight glutenin subunit 1Ax1	3	12.04	31.8	30
D6RVY4	Triticum aestivum	Low molecular glutenin subunit (Fragment)	4	8.49	17.8	14
Q45R38	Triticum aestivum	HMW glutenin x-type subunit Bx7	5	8.16	32.8	33
B6ETR9	Triticum aestivum	D-type LMW glutenin subunit (Fragment)	6	6.16	36.8	21
R4JFK3	Triticum aestivum	Low-molecular-weight glutenin subunit (Fragment)	7	5.71	11.8	4
D0F0C5	Aegilops umbellulata	Low molecular weight glutenin subunit m3	8	5.47	14.9	4
Q00M56	Triticum aestivum	LMW-GS	10	3.85	11.9	4
Q8LKV7	Aegilops tauschii	HMW-glutenin	11	2.41	31.6	40
K7XR61	Triticum aestivum	Alpha-gliadin	12	2.13	4.6	1
Q8W3V4	Triticum aestivum	LMW-glutenin P3-6	13	2.08	4.7	2
A0A0U3AIU6	Aegilops searsii	Low molecular weight glutenin subunit	14	2.01	12.2	4
Q7Y074	Triticum aestivum	Low molecular weight glutenin	15	2.01	4.7	2
Q6R510	Triticum aestivum	HMW glutenin subunit (Fragment)	16	2	38.3	16
A5JJ50	Aegilops geniculata	LMW-glutenin LMW-i1	17	2	5.1	1
W5G4V0	Triticum aestivum	Uncharacterized protein	18	2	17.7	1
W5EL62	Triticum aestivum	40S ribosomal protein S21	19	2	18.3	1
Q8H0B8	Triticum aestivum	Cold regulated protein	20	2	9.1	1
J3LDT4	Oryza brachyantha	Uncharacterized protein	21	2	2.5	1
C5XEJ2	Sorghum bicolor	Glycosyltransferase	22	2	3.5	1
A0A0A9GYX4	Arundo donax	Uncharacterized protein	23	2	36.7	1

**LMW-GS**						
D6RVY4	Triticum aestivum	Low molecular glutenin subunit (Fragment)	1	13.32	34.8	41
I3XHQ1	Triticum aestivum	Low molecular weight glutenin subunit LMW-9	2	12.3	32.6	19
A0A0S2GJT4	Triticum aestivum	Low-molecular-weight glutenin subunit	3	9.11	31.5	24
R4JB44	Triticum aestivum	Low-molecular-weight glutenin subunit (Fragment)	4	8.93	28.3	11
V9P766	Triticum aestivum	LMW-m glutenin subunit 46 (Fragment)	5	8.86	21.2	13
A0A1D8V7C6	Triticum aestivum	High molecular weight glutenin subunit	6	8.03	10.1	5
W5AKY9	Triticum aestivum	Uncharacterized protein	7	6.44	4.9	6
BLAST search: M7KZ46 12S seed storage globulin (identity: 94.5%)						
A0A0U2JFU1	Aegilops speltoides	Low molecular weight glutenin subunit	8	6.14	21.1	10
B2BZD0	Triticum aestivum	LMW-s glutenin subunit 0359D24-S	9	5.82	33.1	47
Q93XQ8	Triticum aestivum	Protein disulfide-isomerase	10	4.59	2.7	2
P04721	Triticum aestivum	Alpha/beta-gliadin A-I	11	4.38	22.5	11
R4JD42	Triticum aestivum	Low molecular weight glutenin subunit (Fragment)	13	4.02	30.2	8
A0A1K0ITH3	Triticum aestivum	Alpha-gliadin	14	3.48	29.3	15
Q03872	Triticum aestivum	High molecular weight glutenin subunit 1Ax1	15	3.47	5.5	4
Q0GNG1	Triticum aestivum	Low molecular weight glutenin subunit	16	3.38	27.6	17
Q5MGR4	Secale sylvestre	Low molecular weight glutenin subunit	17	2.51	26.1	15
I3QPG3	Aegilops crassa	Low molecular weight glutenin subunit t128	18	2.08	16.7	16
Q5XM79	Triticum dicoccoides	Low-molecular-weight glutenin	19	2.05	9.5	4
B5ANT6	Triticum aestivum	LMM glutenin	20	2.01	32.6	27

F1C943	Brachypodium distachyon	Low molecular weight glutenin subunit	21	2.01	16.8	6
R4JDM1	Triticum aestivum	Low-molecular-weight glutenin subunit (Fragment)	22	2	39.1	41
Q8W3X1	Triticum aestivum	Low-molecular-weight glutenin subunit group 3 type II (Fragment)	23	2	31.0	24
R4JBL2	Triticum aestivum	Low-molecular-weight glutenin subunit	24	2	25.1	10
Q5MFH0	Triticum aestivum	Low molecular weight glutenin	25	2	13.9	4
V9P6N2	Triticum aestivum	LMW-i glutenin subunit 1	26	2	20.5	5
V5NDJ9	Triticum dicoccoides	Low-molecular-weight glutenin subunit emmer-3	27	2	13.8	4
B2LWZ2	Aegilops comosa	Low molecular weight glutenin subunit L1	28	2	18.0	12
A5JJ50	Aegilops geniculata	LMW-glutenin LMW-i1	29	2	10.1	4
K4PC84	Aegilops markgrafii	Alpha gliadin	30	2	17.8	4
P01085	Triticum aestivum	Alpha-amylase inhibitor 0.19	31	2	20.2	2
A0A1D5VST3	Triticum aestivum	Uncharacterized protein	32	2	5.4	1
W5FTQ0	Triticum aestivum	Uncharacterized protein	33	2	4.1	1
T1MNX1	Triticum urartu	Uncharacterized protein	34	2	1.3	1
Q8H0B8	Triticum aestivum	Cold regulated protein	35	2	9.1	1
P83207	Triticum aestivum	Chymotrypsin inhibitor WCI	36	2	12.6	1
M7Z0K6	Triticum urartu	Peptidyl-prolyl cis-trans isomerase	38	2	5.5	1
K3ZH02	Setaria italica	Uncharacterized protein	39	2	1.6	1
B6U440	Zea mays	Uncharacterized protein	40	2	8.3	1
B6TY90	Zea mays	Gibberellin receptor GID1L2	41	2	3.7	1
A0A0A9R3C9	Arundo donax	Uncharacterized protein	42	2	16.7	1

**Rye**						
**ω-secalins**						
A0A159KI90	Triticum aestivum	Omega-secalin	1	10.57	64.2	68
W6AW98	Triticum aestivum	High molecular weight glutenin subunit x	2	4.45	10.1	8
**HMW-secalins**						
W6AW92	Triticum aestivum	High molecular weight glutenin subunit y	1	17.33	28.7	38
Q93WF0	Secale cereale	HMW glutenin subunit x	2	14.61	51.2	58
Q43639	Secale cereale	Sec1	3	3.96	22.4	12
V9XX55	Pseudoroegneria strigosa	High molecular weight glutenin subunit	5	2.38	21.2	19
A0A1G4P1T5	Triticum aestivum	HMW glutenin y-type subunit 1By7	6	2.03	4.4	6
E5KZQ0	Secale cereale	75k gamma secalin	7	2	2.1	1
R7W9Z9	Aegilops tauschii	Uncharacterized protein	8	2	8.6	1
K7TU65	Zea mays	Transglutaminase15	9	2	4.4	1
**γ-75k-secalins**						
P52589	Triticum aestivum	Protein disulfide-isomerase	1	8.38	5.6	4
Q94IL2	Secale cereale	High molecular weight glutenin subunit x	2	5.47	7.7	3
E5KZQ1	Secale cereale	75k gamma secalin	3	4.68	53.5	49
Q7M1Z3	Secale cereale	rRNA N-glycosidase	4	4.12	12.9	3
W5GXD6	Triticum aestivum	Uncharacterized protein	6	2.58	14.4	2
A0A287WJQ6	Hordeum vulgare subsp. vulgare	Uncharacterized protein	7	2.5	14.8	2
W5I4U0	Triticum aestivum	Uncharacterized protein	8	2.29	8.8	1
I6QF63	Triticum aestivum	Small heat shock protein	9	2.26	16.6	2
C3VWW0	Secale cereale	Dimeric alpha-amylase inhibitor	10	2.18	5.6	1
A0EPP1	Secale cereale	Puroindoline-a	11	2.05	10.9	2
W5EIR1	Triticum	Uncharacterized protein	12	2.03	9.4	1

	aestivum					
A0A1D8V7C6	Triticum aestivum	High molecular weight glutenin subunit	13	2.02	5.9	4
M8CGY6	Aegilops tauschii	Tubulin alpha chain	14	2	3.6	1
I3RXT5	Secale cereale	Glyceraldehyde-3-phosphate dehydrogenase (Fragment)	15	2	6.0	1
W5BPN7	Triticum aestivum	Uncharacterized protein	16	2	2.2	1
W5AR77	Triticum aestivum	Uncharacterized protein	17	2	6.0	1
A0A1D5ZX82	Triticum aestivum	Uncharacterized protein	18	2	4.2	1
**γ-40k-secalins**						
W5IA32	Triticum aestivum	Formate dehydrogenase, mitochondrial	1	7.41	12.2	3
K3ZAI0	Setaria italica	Uncharacterized protein	2	7.31	35.1	8
W4ZSH7	Triticum aestivum	Uncharacterized protein	3	5.98	40.2	5
Q43223	Triticum aestivum	Sucrose synthase	4	5.62	4.2	3
M0WF40	Hordeum vulgare subsp. vulgare	Uncharacterized protein	5	4.82	6.3	2
F2DB00	Hordeum vulgare subsp. vulgare	Predicted protein	6	4.75	9.5	2
F2DWT1	Hordeum vulgare subsp. vulgare	Predicted protein	7	4.33	17.3	2
M8A1Q4	Triticum urartu	Uncharacterized protein	8	4.05	24.5	2
Q9FR41	Secale cereale	Secalin	10	3.76	8.4	3
W5FRU7	Triticum aestivum	Uncharacterized protein	11	3.67	4.4	2
Q94IL2	Secale cereale	High molecular weight glutenin subunit x	12	3.64	6.1	5
Q9FE55	Triticum turgidum subsp. durum	Protein disulfide-isomerase	13	3.44	2.5	1

I1HGA8	Brachypodium distachyon	Uncharacterized protein	14	3.35	6.3	3
H8Y0K4	Secale cereale	Gamma prolamin	15	3.08	13.8	8
W5C539	Triticum aestivum	GT75-3	16	2.18	6.9	3
C7C4X1	Triticum aestivum	Glyceraldehyde-3-phosphate dehydrogenase	17	2.02	3.6	1
F2DC21	Hordeum vulgare subsp. vulgare	Predicted protein	18	2.02	4.2	1
Q9ST57	Triticum aestivum	Serpin-Z2A	19	2.02	3.8	1
F2DGA8	Hordeum vulgare subsp. vulgare	Predicted protein	20	2	24.5	2
R7WFW5	Aegilops tauschii	Oligopeptidase A	21	2	1.4	1
Q1PBI3	Triticum aestivum	Glucose-6-phosphate isomerase (Fragment)	22	2	3.9	2
A0A1D5UWB4	Triticum aestivum	Uncharacterized protein	23	2	1.8	1
W5I956	Triticum aestivum	Uncharacterized protein	24	2	3.9	1
W5I4R6	Triticum aestivum	Uncharacterized protein	25	2	2.8	1
W5H4V7	Triticum aestivum	Phosphoglycerate kinase	26	2	3.0	1
W5BUF4	Triticum aestivum	Caleosin	27	2	3.7	1
**Barley**						
**C-hordeins**						
A0A287EIM7	Hordeum vulgare subsp. vulgare	Uncharacterized protein	1	7.44	17.1	19
BLAST search: P06472 C-hordein (identity: 99.0%)						
**γ-hordeins**						
A0A287EFG2	Hordeum vulgare subsp. vulgare	Uncharacterized protein	2	3.95	22.2	9
BLAST search: P06470 B1-hordein (identity: 94.6%)						

A0A287Q402	Hordeum vulgare subsp. vulgare	Uncharacterized protein	4	2	9.8	1
**B-hordeins**						
Q84LE9	Hordeum vulgare	D-Hordein	1	13.59	36.3	21
P06470	Hordeum vulgare	B1-hordein	2	11.49	45.7	38
F2D284	Hordeum vulgare subsp. vulgare	Protein disulfide-isomerase	3	9.79	14.8	7
U5NJ12	Hordeum vulgare	Beta-amylase	5	4.65	7.7	3
A0A287NA39	Hordeum vulgare subsp. vulgare	Uncharacterized protein	6	3.99	3.4	2
BLAST search: P06293 Serpin-Z4 (identity: 99.0%)						
A0A287EFF7	Hordeum vulgare subsp. vulgare	Uncharacterized protein	7	2.44	18.0	12
BLAST search: P06470 B1-hordein (identity: 87.5%)						
M0WX05	Hordeum vulgare subsp. vulgare	Uncharacterized protein	8	2.36	2.2	1
BLAST search: M7WY9 Selenium-binding protein (identity: 98.2%)						
F2DGA8	Hordeum vulgare subsp. vulgare	Predicted protein	9	2.17	24.5	3
BLAST search: Q41560 16.9 kDa heat shock protein 2 (identity: 96.7%)						
A0A287WJQ6	Hordeum vulgare subsp. vulgare	Uncharacterized protein	10	2.11	8.6	1
F2E9N0	Hordeum vulgare subsp. vulgare	Predicted protein	11	2.04	2.3	1
BLAST search: M7ZK46 12S seed storage protein (identity: 86.6%)						
A0A287K1V4	Hordeum vulgare subsp. vulgare	Uncharacterized protein	12	2	18.2	3
BLAST search: Q21810 16.9 kDa heat shock protein 1 (identity: 95.4%)						

F2EDK4	Hordeum vulgare subsp. vulgare	Uncharacterized protein	13	2	6.1	1
Q9SME6	Hordeum vulgare	Glutathione peroxidase	14	2	5.1	1
M7Z0K6	Triticum urartu	Peptidyl-prolyl cis-trans isomerase	15	2	5.5	1
P28041	Hordeum vulgare	Alpha-amylase/trypsin inhibitor CMa	16	2	9:7	1
**D-hordeins**						
I6SW34	Hordeum vulgare subsp. vulgare	D hordein	1	33.73	62.1	99
P07597	Hordeum vulgare	Non-specific lipid-transfer protein	2	5.07	25.6	4
A0A287EEX5	Hordeum vulgare subsp. vulgare	Uncharacterized protein	3	4.30	5.8	2
BLAST search: P21292 C-hordein (identity: 50.0%)						
Q2V8X0	Hordeum vulgare	Limit dextrinase inhibitor	4	4.07	20.4	3
P01545	Hordeum vulgare	Alpha-hordothionin	6	2.83	19.7	2
F2DD34	Hordeum vulgare subsp. vulgare	Predicted protein	7	2.21	4.8	1
F2E931	Hordeum vulgare subsp. vulgare	40S ribosomal protein S21	8	2.01	33.3	2
A0A1D8V7C6	Triticum aestivum	High molecular weight glutenin subunit	9	2	11.1	3
A0A287JQN1	Hordeum vulgare subsp. vulgare	Uncharacterized protein	10	2	8.4	1
BLAST search: Q7X8H9 17kDa alpha-amylase/trypsin inhibitor 2 (identity: 49.4%)						
M0Y075	Hordeum vulgare subsp. vulgare	Bowman-Birk type trypsin inhibitor	11	2	6.1	1
K3ZQC5	Setaria italica	Uncharacterized protein	12	2	1.4	1
F2DJM5	Hordeum vulgare subsp.	Uncharacterized protein	13	2	4.3	1

	vulgare					
I1HGA5	Brachypodium distachyon	Putative rRNA methyltransferase	14	2	1.3	1
P15312	Hordeum vulgare	Root-specific lectin	15	2	5.2	1
A0A1W0VS91	Sorghum bicolor	Uncharacterized protein (Fragment)	16	2	2.8	1
P82938	Hordeum vulgare	Unknown endosperm protein C (Fragment)	17	2	100.0	1

[a] Missing rank numbers corresponded to contaminants, [b] unused ProtScore, defined as a measure of the protein confidence for a detected protein, calculated from the peptide confidence for peptides from spectra that are not already completely "used" by higher scoring winning proteins, thus reflecting the amount of total, unique peptide evidence related to a given protein
